# LncCCAT1 Promotes Breast Cancer Stem Cell Function through Activating WNT/β-catenin Signaling

**DOI:** 10.7150/thno.37892

**Published:** 2019-10-01

**Authors:** Tingting Tang, Changying Guo, Tiansong Xia, Rui Zhang, Ke Zen, Yi Pan, Liang Jin

**Affiliations:** 1 State Key Laboratory of Natural Medicines, Jiangsu Key Laboratory of Druggability of Biopharmaceuticals, School of Life Science and Technology, China Pharmaceutical University. 24 Tongjiaxiang Avenue, Nanjing, Jiangsu, China.; 2 Jiangsu Engineering Research Center for microRNA Biology and Biotechnology, State Key Laboratory of Pharmaceutical Biotechnology, School of Life Sciences, Nanjing University, 22 Hankou Road, Nanjing, Jiangsu province, China.; 3 Department of Breast Surgery, Breast Disease Center of Jiangsu Province, First Affiliated Hospital of Nanjing Medical University. 300 Guangzhou Road, Nanjing, Jiangsu province, China.; 4 Department of Biochemistry and Molecular Biology and Key Laboratory of Breast Cancer Prevention and Treatment, Ministry of Education, National Clinical Research Center of Cancer, Tianjin Medical University Cancer Institute and Hospital, Tianjin, China.

**Keywords:** Long noncoding RNA, breast cancer stem cells, self-renew, metastasis, WNT Signaling

## Abstract

**Background:** Breast cancer stem cells (BCSCs) play an essential role in facilitating breast cancer relapse and metastasis. The underlying mechanism, however, remains incompletely understood. In the current study, we investigated the clinical significance, biological function and mechanism of a long noncoding RNA CCAT1 (LncCCAT1) in BCSCs.

**Methods:** Firstly, lncRNAs expression in poorly differentiated breast cancer tissues and BCSCs were measured by lncRNA microarray and confirmed in breast cancer tissues and cell lines. The functional roles and mechanisms of LncCCAT1 were further investigated by gain and loss of function assays *in vitro* and *in vivo*.

**Results:** LncCCAT1 is markedly upregulated in breast cancer tissues BCSCs and is correlated with poor outcomes in breast cancer patients. Overexpression of LncCCAT1 contributes to the proliferation, stemness, migration and invasion capacities of BCSCs. Mechanistic investigation suggests that LncCCAT1 can interact with miR-204/211, miR-148a/152 and Annexin A2(ANXA2), then upregulate T-cell factor 4 (TCF4) or promote translocation of β-catenin to the nucleus where it activates TCF4, leading to the activation of wingless/integrated (Wnt) signaling. Furthermore, TCF4 can also bind to the promoter of LncCCAT1 to promote LncCCAT1 transcription, thus forming a positive feedback regulatory circuit of LncCCAT1-TCF4-LncCCAT1 in BCSCs.

**Conclusions:** LncCCAT1 plays an important role in breast cancer progression and may serve as a novel target for breast cancer diagnosis and therapy.

## Introduction

Breast cancer is the most commonly diagnosed cancer and the leading cause of cancer death among females. Incidence rates of breast cancer have been rising for most countries in transition over the last decades^1^. Actually, the majority of deaths from breast cancer are not due to the primary tumor itself but result from tumor metastasis to other organs in the body; thus, effective methods to prevent relapses or control breast cancer metastases are urgently needed 2. Accumulating evidence indicates that recurrent and distant metastatic tumors are related to a small fraction of breast cancer cells termed breast cancer stem cells (BCSCs) with the surface markers CD44+CD24-/low^3^, ^4^. BCSCs exhibit unique growth abilities, including self-renewal, differentiation potential, and drug resistance, all of which are believed to contribute to the development and aggressiveness of recurrent or metastatic lesions. Therefore, targeting BCSCs might be a novel approach to achieve a breakthrough in the prevention of breast cancer metastasis.

Growing evidence has indicated that aberrant activation of Wnt/β-catenin signaling plays an important role in the initiation and maintenance of cancer stem cells (CSCs), followed by persistent growth in the primary regions, epithelial-to-mesenchymal transition (EMT) activation and CSC re-initiation, which leads to tumor growth and metastasis 5, 6. Hence, inhibition of Wnt signaling through genetic modifications or small molecule inhibitors targeting CSCs has been used as a novel therapeutic approach for cancer treatment 7.

Long noncoding RNAs (lncRNAs) are a heterogeneous class of transcripts with a minimum length of 200 bases and limited protein-coding potential that modulate biological processes via diverse mechanisms, including mobilizing transcriptional coregulators or chromatin-modifying complexes at the transcriptional level, and interacting with RNAs and protein complexes or modifying signal proteins at the post-transcriptional level 8, 9. Recently, several studies found that lncRNAs contribute to the progression of several human cancers and CSC properties 10, 11. Nevertheless, the clinical significance and biological mechanisms of lncRNAs in the regulation of BCSCs remain largely unknown.

In the present study, we first demonstrated that colon cancer-associated transcript-1 (CCAT1), a 2795-bp lncRNA, is highly expressed in both breast cancer tissues and BCSCs and predicts poor prognosis. Next, we revealed that LncCCAT1 promotes the proliferation, stemness, migration and invasion of BCSCs. Further mechanistic investigations *in vitro* and *in vivo* indicated that cytoplasmic LncCCTA1 upregulates TCF4 expression by competitive binding to miR-204/211 and promotes β-catenin translocation into the nucleus through interaction with miR-148a/152 and ANXA2, which finally leads to the activation of Wnt/β-catenin signaling, EMT progress, and LncCCAT1 transcription. Therefore, our results provide the first evidence that LncCCAT1 plays a crucial role in breast cancer progression and metastasis by modulating BCSC functions and may serve as a novel target for breast cancer diagnosis and therapy.

## Materials and Methods

### Cell lines and sphere formation assay

Breast cancer cell lines MCF-7 and MDA-MB-231 cells were purchased from the Institute of Biochemistry and Cell Biology of the Chinese Academy of Sciences (Shanghai, China). Cells were maintained in DMEM (Gibco, CA, USA) or L-15 (Gibco) medium supplemented with 10% fetal bovine serum (FBS), 100U/ml penicillin (Invitrogen, CA, USA) and 100 ng/ml streptomycin (Invitrogen) in humidified air at 37℃ with 5% CO_2_. For sphere formation assay, cells were seeded into the 24-well ultra-low attachment plate (Corning, NY, USA) in serum-free DMEM/F12 (Invitrogen), supplemented with B27 (1:50, Invitrogen), 20 ng/ml EGF (Peprotech, NJ, USA), 20 ng/ml bFGF (Invitrogen), 100U/ml penicillin and 100 ng/ml streptomycin, 4 µg/ml insulin (Sigma, MO, USA) and 20% methylcellulose (Sigma). Cells were incubated in a CO_2_ incubator for two weeks, and numbers of spheroid cells were counted under a stereomicroscope (Olympus, Tokyo, Japan). All of the cells were authenticated by short tandem repeat (STR) profiling (Cobioer Biosciences).

### Patients and clinical specimens

All patient samples were collected from the Breast Disease Center of Jiangsu Province, First Affiliated Hospital of Nanjing Medical University (Nanjing, China) with written informed consent. The ethical approval was granted from Committees for Ethical Review in China Pharmaceutical University (Nanjing, China). Pathological diagnosis was made according to the histology of tumor specimens or biopsy and examined by experienced pathologists. The clinicopathological characteristics are shown in Table S1. Breast cancer tissues and adjacent normal tissues were stored in liquid nitrogen. The study is compliant with all relevant ethical regulations for human research participants, and all participants provided written informed consent.

### LncRNA microarray analysis

Total RNAs were isolated with Trizol from CD44+CD24- and non-CD44+CD24- cells derived from human breast cancer cell line MCF-7; and isolated from 3 paired poorly differentiated breast cancer tissues (tumor grade III) and adjacent normal tissues. The microarray profiling was carried out in the lab of Shanghai OE Biotech Company. Briefly, total RNAs were transcribed to double strand cDNA by using The Ambion® WT Expression Kit, then synthesized into cRNA and labeled with Cyanine-3-CTP by using WT Terminal Labeling and Controls Kit. Then the labeled cRNAs were hybridized, washed and stained in GeneChip® Hybridization, Wash, and Stain Kit. Next, GeneChips were scanned by using Affymetrix® GeneChip Command Console (AGCC) that installed in GeneChip® Scanner 3000. At last, Robust Multichip Analysis (RMA) normalization for gene level analyses was completed by Expression Console (version 1.3.1, Affymetrix) software. GeneSpring software (version 13.1, Agilent Technologies) was employed to identify aberrant gene expression analyses through fold change as well as a P-value calculated using Student's *t*-test. The threshold set for aberrantly regulated genes was a fold change ≥2.0.

### RNA immunoprecipitation (RIP) and chromatin immunoprecipitation (ChIP)

For MS2-based RIP assays, MCF-7 cells were co-transfected with pcDNA3.1-MS2, pcDNA3.1-MS2-CCAT1, pcDNA3.1-MS2-CCAT1-MUT and pMS2-GFP (Addgene). After 48h, cells were used to perform RNA immunoprecipitation (RIP) experiments using a GFP antibody and the Magna RIP™ RNA-Binding Protein Immunoprecipitation Kit (Millipore, Bedford, MA) according to the manufacturer's instructions. The RNA fraction isolated by RIP was quantified using a Nano-Photometer spectrophotometer in the UV and visible spectra (Implen, Munich, Germany). qRT-PCR was used to evaluate the expression levels of miRNAs. For Ago2-based RIP assays, MCF-7 and MDA-MB-231 cells were transfected with pcDNA3.1-CCAT1 or pcDNA3.1. After 48 h, cells were used to perform RIP experiments using an anti-Ago2 antibody and the Magna RIP™ RNA-Binding Protein Immunoprecipitation Kit (Millipore) according to the manufacturer's instructions. qRT-PCR was performed to examine the expression levels of LncCCAT1 and miR-148a, miR-152, miR-204 and miR-211.ChIP assays were performed using EZ-ChIP Kit (Millipore). Chromatin was immunoprecipitated with indicated antibodies and analyzed by PCR. Sequences of primers used for qRT-PCR, plasmid construction and ChIP-qPCR were listed in Table S2.

### Immunofluorescence and Immunohistochemistry assay

For immunofluorescence analysis, cells were fixed with 4% formaldehyde followed by incubation with anti-β-catenin, anti-ANXA2 or anti-GSK3β followed by Alexa Fluor 488 anti-rabbit secondary antibody and Alexa Fluor 555 anti-mouse secondary antibody (Invitrogen) incubation. The nuclear was stained with DAPI (Invitrogen). Images were obtained using confocal laser microscope (Carl Zeiss) with a ×63/1.40NA objective. For immunohistochemistry assay Paraffin-embedded sections were deparaffinized and rehydrated, followed by antigen retrieval. After primary and secondary antibody incubation, the slides were incubated with diaminobenzidine (DAB) and counterstained with hematoxylin (Sigma). Immunohistochemistry of TCF4 and Ki67, after incubating with horseradish peroxidase-conjugated IgG (1:500; Invitrogen) the proteins *in situ* were visualized with 3, 3-diaminobenzidine. Antibodies used for western blotting (WB), immunoprecipitation (IP), immunofluorescence (IF) and flow cytometry (FC) are provided in Table S3.

### LncCCAT1 knockout by CRISPR

To obtain stable cell lines with downregulation of LncCCAT1, GenScript^TM^ Cas9 nuclease (GenScript, Nanjing, China) was used. 4 pairs of sgRNAs were designed and synthesized, after screening the cutting efficiency of sgRNAs *in vitro*, the most productive sgRNAs and Cas9 protein were co-transfected into MCF-7 and MDA-MB-231 cells. After 48h, cells were sorted by GFP using flow cytometry and single clone cell was selected and culture to verify the knock out efficiency of LncCCAT1 by qRT-PCR and PCR. Sequences of siRNAs and sgRNAs against specific targets are listed in Table S4.

### Animal experiments

Female Balb/c nude mice were cared according to Provisions and General Recommendation of Chinese Experimental Animals Administration Legislation. The procedure of all animal experiments complied with IACUC (Institutional Animal Care and Use Committee) regulations. MCF-7 cells were stably transfected with empty vector, LncCCAT1 recombinant lentiviruses, LncCCAT1 lentivirus together with pre-miR-148a/152 recombinant lentiviruses, pre-miR-204/211 recombinant lentiviruses, or ANXA2 shRNA lentiviruses. To determine the tumor formation ability, *in vivo* limiting dilution assay of indicated MCF-7 cells was performed. A series of 1×10^4^, 1×10^5^, 1×10^6^ cells were injected into five-week-old female BALB/c nude mice (n=8 per group), and the tumor-initiating frequency was calculated. Additionally, the subcutaneous xenograft mouse model was used to assess tumor growth, 2 × 10^6^ indicated different infected MCF-7 cells in 0.2 ml PBS were subcutaneously injected into the right armpit region of five-week-old female BALB/c nude mice which were randomly divided into five groups (n=6 per group). Xenograft volumes were evaluated by caliper measurements of two perpendicular diameters and calculated individually as formula: Volume = a × b^2^/2 (a represent length and b represent width). 24 days after injection, the mice were sacrificed and the subcutaneous tumors were isolated and measured. For metastasis experiments, MDA-MB-231 cells were stably transfected with empty vector or MDA-MB-231-CCAT1-KO cells, or MDA-MB-231-CCAT1-KO cells transfected with miR-148a/152 inhibitors recombinant lentiviruses, miR-204/211 inhibitors recombinant lentiviruses, or ANXA2 overexpression lentiviruses. 1 × 10^6^ above cells in 0.1 ml PBS were injected into the tail vein of five-week-old female BALB/c nude mice which were randomly divided into six groups (n=6 for each group). After injection, metastases were examined by bioluminescence imaging using an IVIS Spectrum Xenogen Imaging System (Xenogen, CA, USA) on day 0, 20 and 40. After scanning, intact lungs and livers were isolated from the mice and photographed. The tissue sections were stained with hematoxylin and eosin. For all animal experiments, the operators and investigators were blinded to the group allocation. All animal experiments were approved by the Ethics Committee of China Pharmaceutical University. Permit Number: 2162326.

### Statistical analysis

All statistical analyses were performed using SPSS 20.0 software (IBM, SPSS, USA). Data are listed as mean value ± s.d. Student's *t*-test or χ^2^ test was used when the variance between groups are similar, and the Wilcoxon signed rank test was used when the variance between groups are not similar The Kaplan-Meier method with log-rank test, Spearman's correlation analysis, and Cox multivariate analysis were used as mentioned above. Variables with a value of *P* < 0.05 in univariate analysis were used in subsequent multivariate analysis on the basis of Cox regression analyses. Two-sided *P*-values were calculated, and a probability level of 0.05 was chosen for statistical significance.

### Accession numbers

The lncRNA microarray data were deposited in the NCBI's Gene Expression Omnibus (GEO) database (GSE125677 and GSE125678).

## Results

### LncCCAT1 is upregulated in both breast cancer tissues and BCSCs

**Figure 1:**
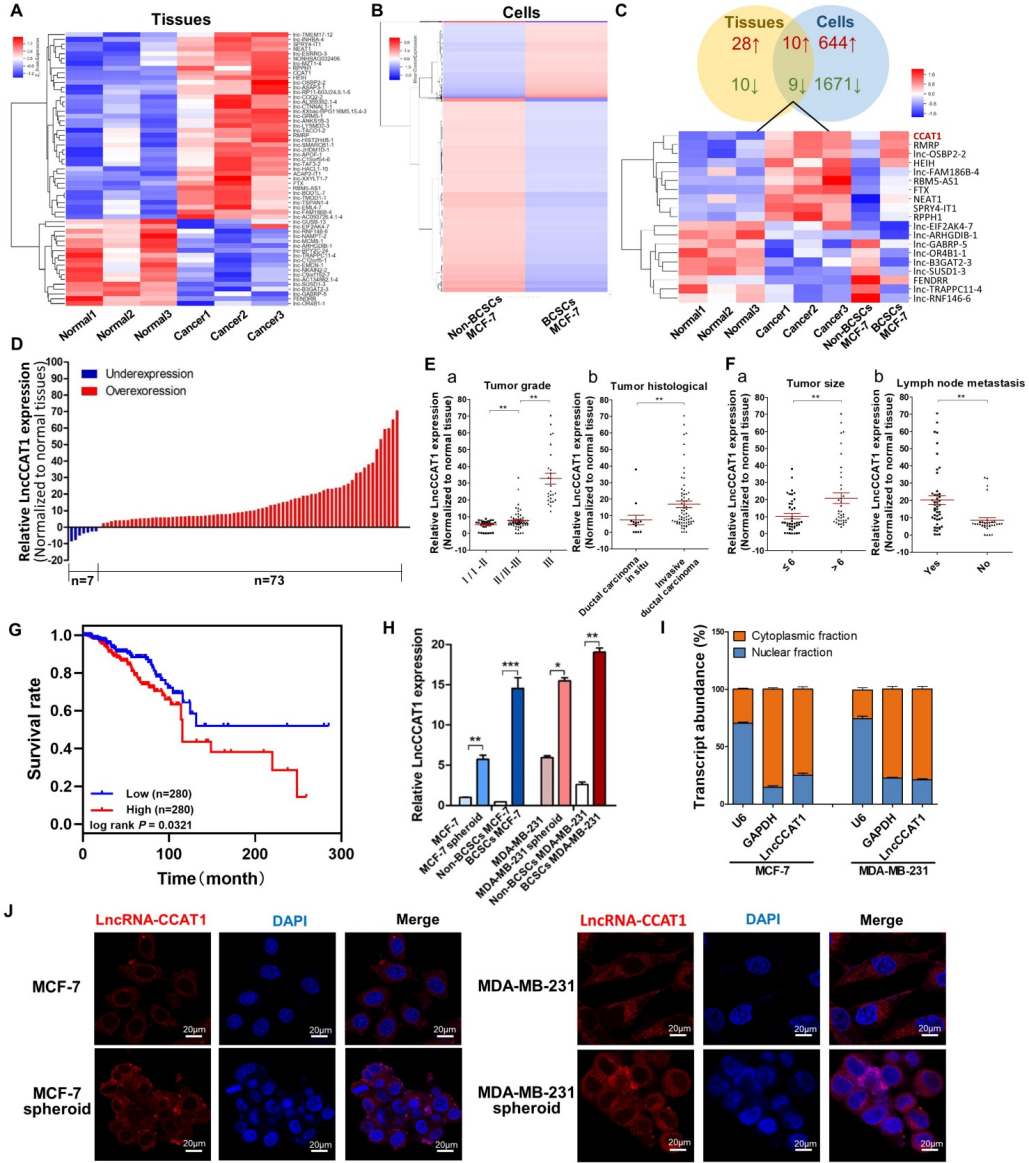
** LncCCAT1 is upregulated in breast cancer tissues and BCSCs.** (**A** and **B**) Hierarchical clustering analysis of all the significantly (>2.0-fold) upregulated or downregulated lncRNAs in breast cancer tissues compared to adjacent normal tissues (A) and in CD44+CD24- MCF-7 cells (BCSCs) compared to non-CD44+CD24- MCF-7 cells (non-BCSCs) (B). Expression values are represented in shades of red and blue, indicating the expression above and below the median expression value, respectively. (**C**) Graphical representation (upper) and hierarchical clustering analysis (lower) of the overlapped regulated genes (19 genes) between the tissues and cells lncRNA microarrays. (**D**) LncCCAT1 levels detected in 80 paired breast cancer tissues by qRT-PCR. (**E, F**) LncCCAT1 levels in breast cancer patients with different tumor grades (E, a) and histological (E, b), and in patients with different tumor size (F, a), with (indicated with 'yes') or without (indicated with 'no') lymph node metastasis (F, b). (**G**) TCGA database indicated that LncCCAT1 was correlated with a poor survival rate by Kaplan-Meier survival analysis (*P*<0.05). (**H**) LncCCAT1 levels were detected in different cells as indicated by qRT-PCR. (**I**) Distribution of LncCCAT1 in breast cancer cells detected by fractionation of MCF-7 and MDA-MB-231 cells followed by qRT-PCR. U6 and GAPDH RNAs served as the positive control for nuclear gene expression and cytoplasmic gene expression, respectively. (**J**) FISH analysis of LncCCAT1 in MCF-7 cells, MDA-MB-231 cells and their spheroid cells. The nuclei were stained with DAPI. Scale bar, 20 μm. All data are shown as the mean±SD. *P<0.05, **P <0.01, and ***P<0.001 by two-tailed Student's *t*-test.

To identify crucial lncRNAs involved in breast cancer progression driven by BCSCs, we utilized microarrays to compare lncRNA expression profiles between 3 paired poorly differentiated (tumor grade III) breast cancer tissues and adjacent normal tissues and between flow cytometry-sorted non-CD44+CD24-MCF-7 cells (non-BCSCs) and CD44+CD24- MCF-7 cells (BCSCs).The above 3 tissues and 1 cell line belongs to 4 subtypes of breast cancer: Luminal A (MCF-7), Luminal B, HER2-enriched, and Triple-negative/basal-like, respectively. As shown in Figure 1A and B, heatmaps identified systematic variations in lncRNA transcript levels in the tissues and cells. From 63542 human lncRNAs, 38 upregulated and 19 downregulated lncRNAs in the 3 paired breast cancer tissues (Figure 1A) and 654 upregulated and 1680 downregulated lncRNAs in the BCSCs MCF-7 cells were identified by the microarray analysis (Figure 1B). Among the 19 genes regulated in both breast cancer tissues and BCSCSs MCF-7 cells (Figure 1C), LncCCTA1, which was highly ranked, particularly drew our attention.

Next, the upregulation of LncCCAT1 was validated in 80 paired breast cancer tissues and adjacent normal tissues. As shown in Figure 1D, LncCCAT1 expression was significantly upregulated in 91.25% (73 of 80 paired) of the breast cancer tissues. We further divided the samples into high (above the median, n=40) and low (below the median, n=40) LncCCAT1 expression groups according to the median value of LncCCAT1 levels and then explored the correlation between LncCCAT1 expressions and the clinicopathological factors of breast cancer patients. As shown in Table S1 and Figure 1E and F, LncCCAT1 level was positively associated with tumor grade, tumor histological, tumor size and lymph node metastasis in this study by χ^2^ tests. Meanwhile, we evaluated the correlation between LncCCAT1 expression and clinical outcomes from TCGA database using Kaplan-Meier analysis and log-rank tests. As shown in Figure 1G, LncCCAT1 overexpression predicts a poor prognosis in patients with breast cancer (n=560, *P*<0.05). We also analyzed the different clinical outcomes associated with LncCCAT1 according to the subtypes of breast cancer. As shown in Figure S1A, LncCCAT1 overexpression predicts a poor prognosis in patients with Luminal A breast cancer (n=256, *P*<0.05) while has no significant relationship with prognosis in patients with Luminal B (n=63), Triple-negative/basal-like (n=64) or HER2-enriched (n=22) breast cancer (data not shown), which might be due to the small sample sizes of Luminal B, Triple-negative/basal-like and HER2-enriched breast cancer patients.

Furthermore, we cultured BCSC-enriched MCF-7 and MDA-MB-231 spheroid cells using a 3D semi-solid system 12, sorted CD44+CD24- MCF-7 cells and CD44+CD24-ESA+ MDA-MB-231 cells as BCSCs, and then detected LncCCAT1 levels in these cells. Consistent with the microarray results, LncCCAT1 expression levels were increased in spheroid cells and BCSCs compared with adherent cells and non-BCSCs, respectively (Figure 1H). Through cellular fractionation assays (Figure 1I) and RNA fluorescence *in situ* hybridization (FISH) (Figure 1J), we found that LncCCAT1 was mainly localized in the cytoplasm of breast cancer cells, and LncCCAT1 levels were higher in spheroid cells than in adherent cells. Taken together, these data confirmed that LncCCAT1 is highly expressed in breast cancer tissues and BCSCs and may serve as an independent predictor for overall survival in breast cancer.

### LncCCAT1 is required for maintaining BCSC stemness and promoting BCSC proliferation, migration and invasion

**Figure 2:**
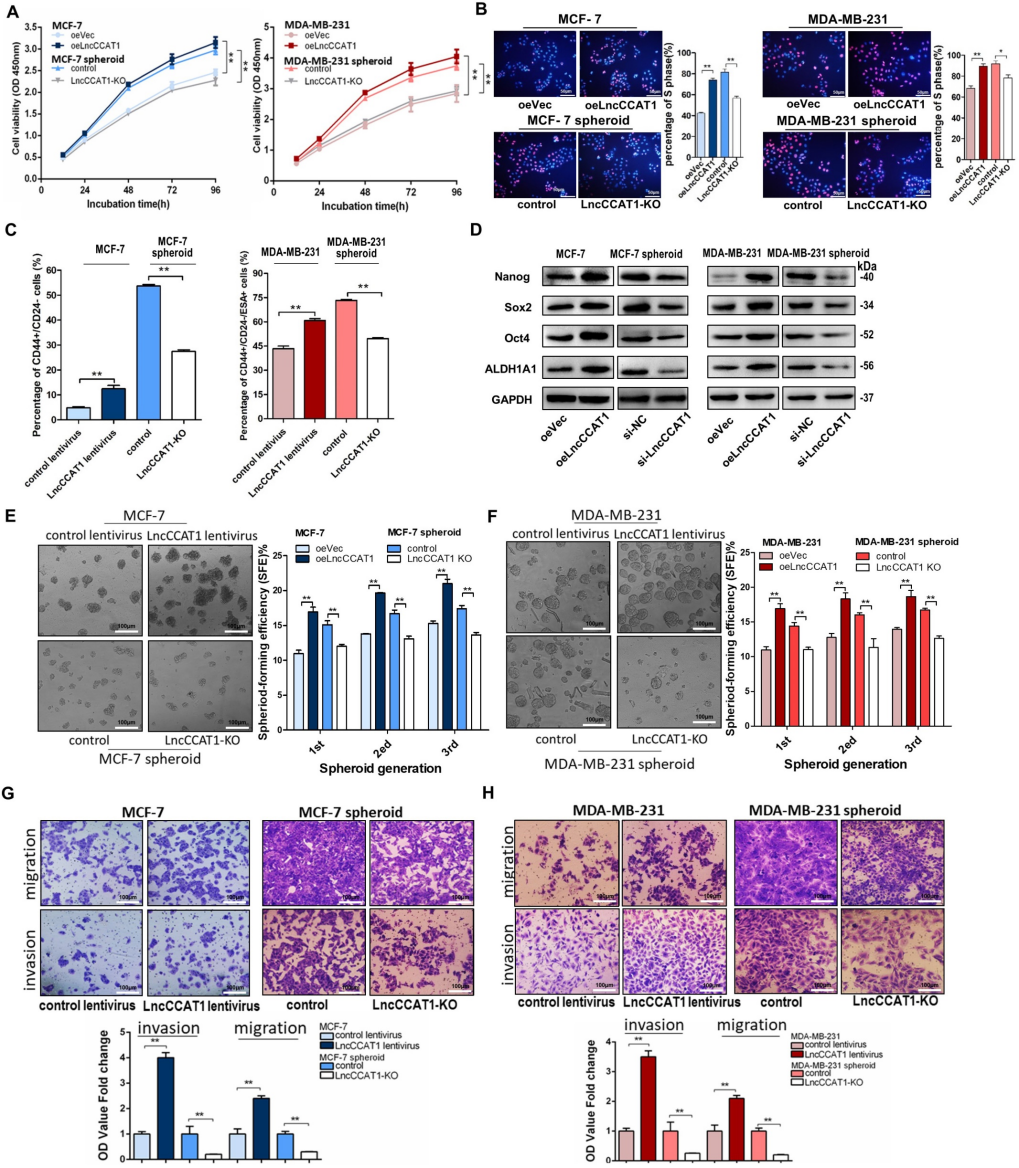
**LncCCAT1 promotes breast cancer cell proliferation, stemness, migration, and invasion *in vitro*.** (**A, B**) Proliferative abilities of LncCCAT1-overexpressing adherent cells and LncCCAT1-knockdown spheroid cells of the MCF-7 cell line or MDA-MB-231 cell line detected by CCK-8 assays (A) and EdU assays (B). (**C**) Percentage of CD44+CD24- or CD44+CD24-ESA+ cells in LncCCAT1-overexpressing adherent cells and LncCCAT1-KO spheroid cells of MCF-7 cells or MDA-MB-231 cells, respectively, detected by flow cytometric analysis. (**D**) Protein levels of pluripotent transcription factors in LncCCAT1-overexpressing MCF-7 and MDA-MB-231 cells and LncCCAT1-knockdown MCF-7 and MDA-MB-231 spheroid cells. (**E, F**) Sphere-formation abilities of LncCCAT1-overexpressing adherent cells and LncCCAT1-KO spheroid cells of MCF-7 cells (E) or MDA-MB-231 cells (F). The sphere-formation efficiencies (SFEs) are shown in the right panels. Scale bar, 100 μm. (**G, H**) Migration and invasion of LncCCAT1-overexpressing adherent cells and LncCCAT1-KO spheroid cells of MCF-7 cells (G) or MDA-MB-231 cells (H) detected by transwell assays. Scale bar, 100 μm. All data are shown as the mean±SD. *P <0.05, ** P<0.01, and ***P<0.001 by two-tailed Student's *t*-test.

To explore the potential role of BCSC LncCCAT1 in promoting breast cancer progression, we used siRNA or a dual CRISPR/Cas9 system to knock down or knock out (KO) the exogenous expression of LncCCAT1 in MCF-7 and MDA-MB-231 spheroid cells and used an overexpression plasmid (oeLncCCAT1) or lentivirus to upregulate LncCCAT1 in MCF-7 and MDA-MB-231 adherent cells (Figure S1B-H). The Cell Counting Kit-8 (CCK-8) assays and EdU assays showed that LncCCAT1 overexpression significantly promoted adherent cell proliferation, while LncCCAT1 depletion inhibited the proliferative abilities of spheroid cells (Figure 2A and 2B). Flow cytometric analysis showed that LncCCAT1 overexpression increased the proportion of BCSCs in adherent cells, while LncCCAT1-KO remarkably reduced the BCSC proportion in spheroid cells (Figure 2C and Figure S2A). Notably, LncCCAT1 overexpression and knockdown significantly altered the expression of the pluripotent transcription factors Nanog, Sox2, Oct4, and ALDH1A1 compared with those of control cells (Figure 2D and Figure S2B). Importantly, LncCCAT1 overexpression dramatically increased the sphere-formation efficiency of breast cancer cells, while LncCCAT1-KO impaired self-renewal capacity on serial passaging (Figure 2E and F). Collectively, these results demonstrated that LncCCAT1 plays a critical role in proliferation and stemness induction and maintenance of BCSCs. Next, we examined the effect of LncCCAT1 on BCSC migration and invasion by transwell assays (Figure 2G and H, Figure S2C and D). LncCCAT1 overexpression promoted cell migration and invasion in MCF-7 and MDA-MB-231 cells, while LncCCAT1-KO impaired the migration and invasion of spheroid cells. These findings indicated that LncCCAT1 is important for breast cancer metastasis.

### LncCCAT1 facilitates TCF4 expression by competing for miR-204/211

**Figure 3:**
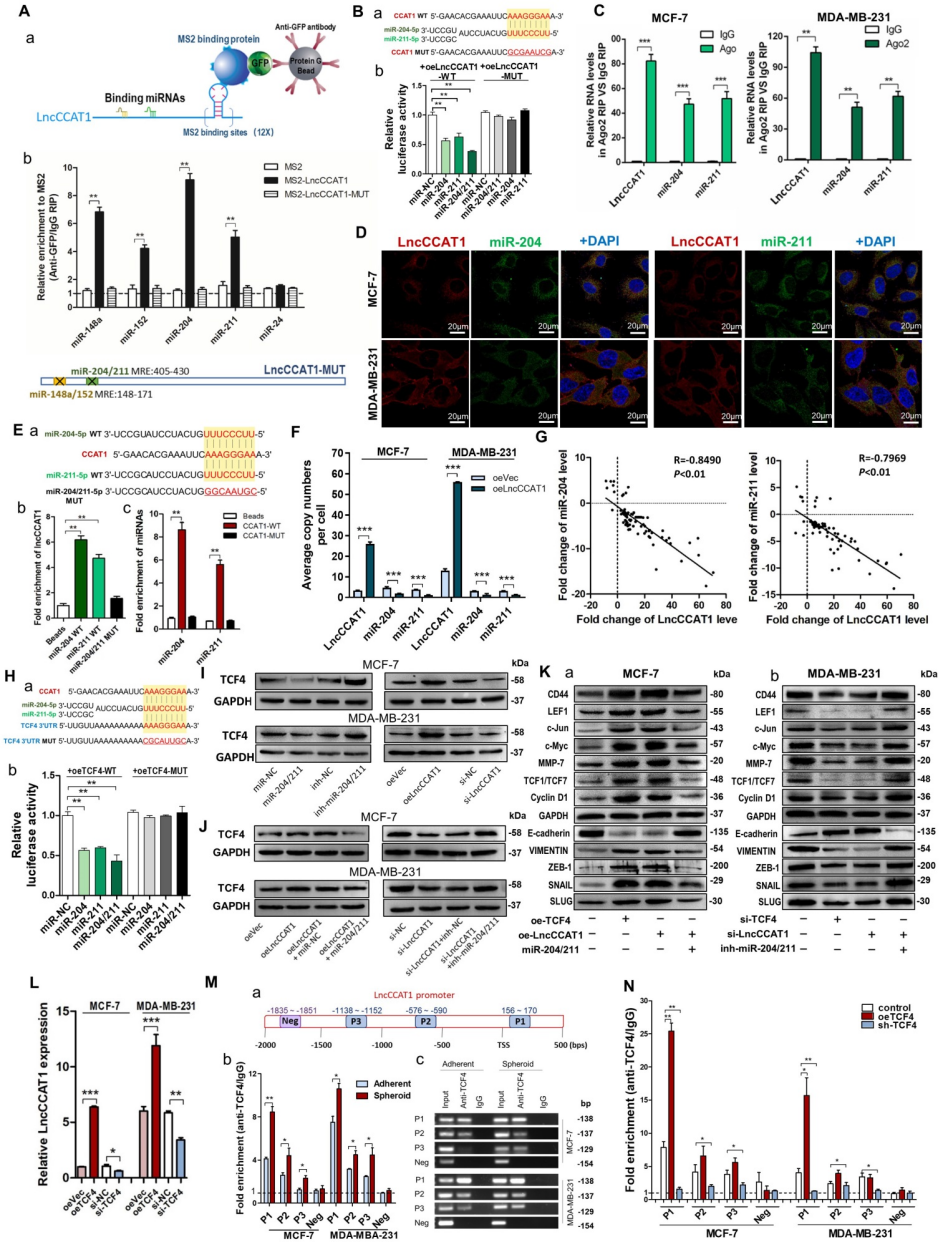
** LncCCAT1 upregulates TCF4 by competitively binding to miR-204/211.** (**A**) A schematic diagram (a) of the MS2-RIP method used to detect endogenous microRNAs associated with LncCCAT1 in MCF-7 cells. miR-204/211 and miR-148a/152 were significantly enriched in RNAs retrieved from MS2-LncCCAT1 compared to control or MS2-LncCCAT1-MUT detected by qRT-PCR (b). miR-24 acted as a negative control. (**B**) Putative binding sequences between miR-204/211 and LncCCAT1 (WT or MUT) (a). Luciferase activity in MCF-7 cells co-transfected with a luciferase reporter containing LncCCAT1-WT or LncCCAT1-MUT (miR-204/211-binding sequence mutated) and miR-204/211 mimics (b). (**C**) Relative enrichment of LncCCAT1, miR-204, and miR-211 associated with AGO2 in MCF-7 and MDA-MB-231 cells detected by anti-AGO2 RIP (non-specific IgG as negative control). (**D**) RNA-FISH assays indicated the colocalization of LncCCAT1 (red dots) and miR-204 (green dots) or miR-211 (green dots) in MCF-7 and MDA-MB-231 cells. Scale bar, 20 μm. (**E**) Putative binding sequences between miR-204/211 (WT) and LncCCAT1 in miR-204/211 (WT) were mutated in miR-204/211 (MUT) (a); pulldown assays using biotin-labeled miR-204 or miR-211 (b) or biotin-labeled LncCCAT1 (c) in MCF-7 cells. (**F**) The exact copy numbers of LncCCAT1, miR-204 and miR-211 per cell in control and LncCCAT1-overexpressing MCF-7 and MDA-MB-231 cells. (**G**) The correlation between miR-204 or miR-211 and LncCCAT1 in breast cancer tissues by Spearman correlation analysis. (**H**)The RNA algorithm predicted potential binding sites of miR-204/211 with LncCCAT1 or with TCF4, with considerable sequence complementary in the indicated regions(a). Luciferase activity in MCF-7 cells cotransfected with a luciferase reporter containing either TCF4-WT or TCF4-MUT (miR-204/211-binding sequence mutated) and miR-204/211 mimics(b). Data are presented as the relative ratio of renilla luciferase activity and firefly luciferase activity. (**I, J**) TCF4 protein levels in MCF-7 and MDA-MB-231 cells transfected with miR-204/211 mimics or inhibitors, oeLncCCAT1, si-LncCCAT1 or their controls (I), and transfected with oeVec, oeLncCCAT1, oeLncCCAT1 plus miR-NC, or oeLncCCAT1 plus miR-204/211 mimics (J). (**K**) The protein levels of Wnt/β-catenin signaling and EMT target genes in MCF-7 cells transfected with oeVec, oeTCF4, oeLncCCAT1 and miR-204/211 mimics (a) and MDA-MB-231 cells transfected with si-NC, si-TCF4, si-LncCCAT1 and miR-204/211 inhibitors (b). (**L**) LncCCAT1 levels in MCF-7 and MDA-MB-231 cells transfected with oeVec or oeTCF4 detected by qRT-PCR. (**M**) Putative TCF4 binding sites on the promoter region of LncCCAT1. A random region (Neg) without DNA binding elements (DBEs) of TCF4 served as a negative control (a). The enrichment of TCF4 on the LncCCAT1 promoter relative to IgG in breast cancer adherent cells and spheroid cells detected by ChIP assays. An approximately 156-170 bp fragment of the LncCCAT1 promoter is sufficient for the binding of TCF4 by qRT-PCR analysis (b) and PCR analysis (c). (**N**) The enrichment of TCF4 on the LncCCAT1 promoter relative to IgG in MCF-7 and MDA-MB-231 cells transfected with oeVec and sh-NC (control), sh-NC and oeTCF4 (oeTCF4), or oeVec and sh-TCF4 (sh-TCF4) detected by ChIP-qPCR assays. All data are shown as the mean±SD. *P<0.05, **P<0.01, and ***P<0.001 by two-tailed Student's *t*-test.

Cytoplasmic lncRNAs usually act as competing endogenous RNAs (ceRNA) by binding miRNAs 13. To examine whether LncCCAT1 can bind endogenous miRNAs, we predicted putative miRNA-binding sites located in the primary transcript of LncCCAT1 by StarBase and AnnoLnc software and then calculated the minimum free energy of hybridization using RNAhybrid. Twelve candidate miRNAs involved in carcinogenesis were selected for further analysis. We performed MS2 RNA immunoprecipitation (MS2-RIP) in MCF-7 cells to pull down endogenous miRNAs associated with LncCCAT1 and detected the above miRNAs via qRT-PCR. As shown in Figure S3A, transfection of pcDNA3.1-MS2-CCAT1 upregulated LncCCAT1 expression level without affecting the distribution of LncCCAT1 in MCF-7 cells. The MS2-RIP results showed that miR-148a/152 and miR-204/211 were significantly enriched in MS2-LncCCAT1 instead of empty vector (MS2) or MS2-LncCCAT1-MUT, in which the above 4 miRNA response elements (MREs) were mutated (Figure 3A and Figure S3B).

Next, we confirmed the specific interaction between LncCCAT1 and miR-204/211. The luciferase reporter assay showed that miR-204/211 overexpression reduced the luciferase activities of the LncCCAT1-WT reporter vector but not the LncCCAT1-MUT reporter (Figure 3B). RIP assays performed on MCF-7 and MDA-MB-231 cell extracts using antibodies against Ago2 demonstrated that LncCCAT1 and miR-204/211 were all obviously enriched in Ago2-immunoprecipitation (Ago2-IP) relative to control IgG immunoprecipitation (Figure 3C). RNA-FISH showed that LncCCAT1 colocalized with miR-204/211 in the cytoplasm of MCF-7 and MDA-MB-231 cells (Figure 3D). An RNA pulldown assay using biotin-labeled miR-204/211 and a pulldown assay using biotin-labeled LncCCAT1 were performed and the putative binding sequences between miR-204/211 (WT or MUT) and LncCCAT1 were shown (Figure 3E, a). LncCCAT1 was pulled down by miR-204/211-WT, whereas miR-204/211-MUT with a disrupting putative binding sequence failed to coprecipitate LncCCAT1 (Figure 3E, b). In parallel, miR-204/211 was precipitated by LncCCAT1-WT instead of LncCCAT1-MUT (Figure 3E, c). These results confirmed that the recognition of miR-204/211 by LncCCAT1 was sequence-specific. As a ceRNA, the abundance of LncCCAT1 and miR-204/211 should be comparable. We therefore used qRT-PCR to quantify the exact copy numbers of LncCCAT1 and miR-204/211 in control and LncCCAT1-overexpressing MCF-7 and MDA-MB-231 cells. As shown in Figure 3F, the copies of LncCCAT1 per cell was similar to that of miR-204/211 in MCF-7 and MDA-MB-231 cells. Moreover, LncCCAT1 overexpression decreased the copy numbers of miR-204/211. Furthermore, we found that miR-204/211 was downregulated in breast cancer tissues compared with adjacent normal tissues (Figure S4A and B), and LncCCAT1 levels were negatively associated with miR-204/211 levels in breast cancer tissues (Figure 3G) and cell lines (Figure S4C). Intriguingly, miR-204/211 levels were negatively regulated by LncCCAT1 but were not affected by LncCCAT1-MUT (Figure S4D), suggesting that LncCCAT1 could repress the expression of miR-204/211 via direct binding at the MRE.

To further elucidate the significance of the LncCCAT1-miR-204/211 axis in BCSC functions, we used TargetScan, miRanda and PicTar software, and TCF4, which functions as a major transcriptional activator of the canonical Wnt signaling pathway together with β-catenin coactivator, was a predicted target of miR-204/211. The predicted target sites among LncCCAT1, miR-204/211 and the TCF4 3' untranslated region (UTR) illustrated (Figure 3H, a). Luciferase reporter assays demonstrated that miR-204/211 could bind to the 3'-UTR of TCF4 (Figure 3H, b). miR-204/211 overexpression or LncCCAT1 knockdown significantly repressed the expression of TCF4 protein in breast cancer cells, while miR-204/211 downregulation or LncCCAT1 overexpression upregulated TCF4 protein levels (Figure S3C, Figure 3I). However, miR-204/211 had no significant effect on TCF4 mRNA expression (Figure S4E). Next, to prove that the regulation of TCF4 by LncCCAT1 was mediated by miR-204/211, we conducted the rescue experiments. As shown in Figure 3J, miR-204/211 overexpression or knockdown abolished the effect of oeLncCCAT1 or si-LncCCAT1 on TCF4 expression.

We further investigated whether LncCCAT1 can activate the Wnt signaling pathway by upregulating TCF4 through competition for miR-204/211. As shown in Figure 3K, overexpression of TCF4 or LncCCAT1 activated Wnt signaling and EMT, while overexpression of miR-204/211 repressed the promoting effect of LncCCAT1 on Wnt signaling and EMT. Meanwhile, in MDA-MB-231 cells, knockdown of TCF4 and LncCCAT1 significantly restrained the activation of Wnt signaling and EMT, while knockdown of miR-204/211 abolished the inhibitory effect of LncCCAT1. Moreover, TCF4 overexpression promoted the proliferation, migration, and self-renewal of MCF-7 cells, and silencing of TCF4 in MDA-MB-231 cells achieved the opposite effect; these findings were consistent with the functions of LncCCAT1 overexpression or depletion (Figure S5A-E). The data showed that LncCCAT1 could activate Wnt signaling and EMT by inhibiting miR-204/211 and promoting TCF4 protein expression.

### TCF4 activates the transcription of LncCCAT1

Given that TCF4 is a transcription factor and studies have shown that many regulatory feed-forward loops are involved in tumor progression 14, we tested whether TCF4 could regulate the expression of LncCCAT1. As shown in Figure 3L, the expression of LncCCAT1 was upregulated by TCF4 overexpression but decreased by TCF4 knockdown. Subsequently, we predicted three DNA binding elements (DBEs) for TCF4 in the promoter region of LncCCAT1 by bioinformatics analysis (Figure 3M, a). A chromatin immunoprecipitation (ChIP) assay showed that TCF4 was enriched in the promoter region of LncCCAT1, which was significantly enriched in the spheroid cells (Figure 3M, b & c). Furthermore, TCF4 overexpression or knockdown could increase or decrease the enrichment of TCF4 in the LncCCAT1 promoter region, respectively (Figure 3N). Taken together, these data indicated that TCF4 could promote LncCCAT1 transcription, thus forming a positive feedback circuit in BCSCs.

### LncCCAT1 functions as a ceRNA for miR148a/152 to upregulate DNMT1 and inhibit FAT4 expression

**Figure 4:**
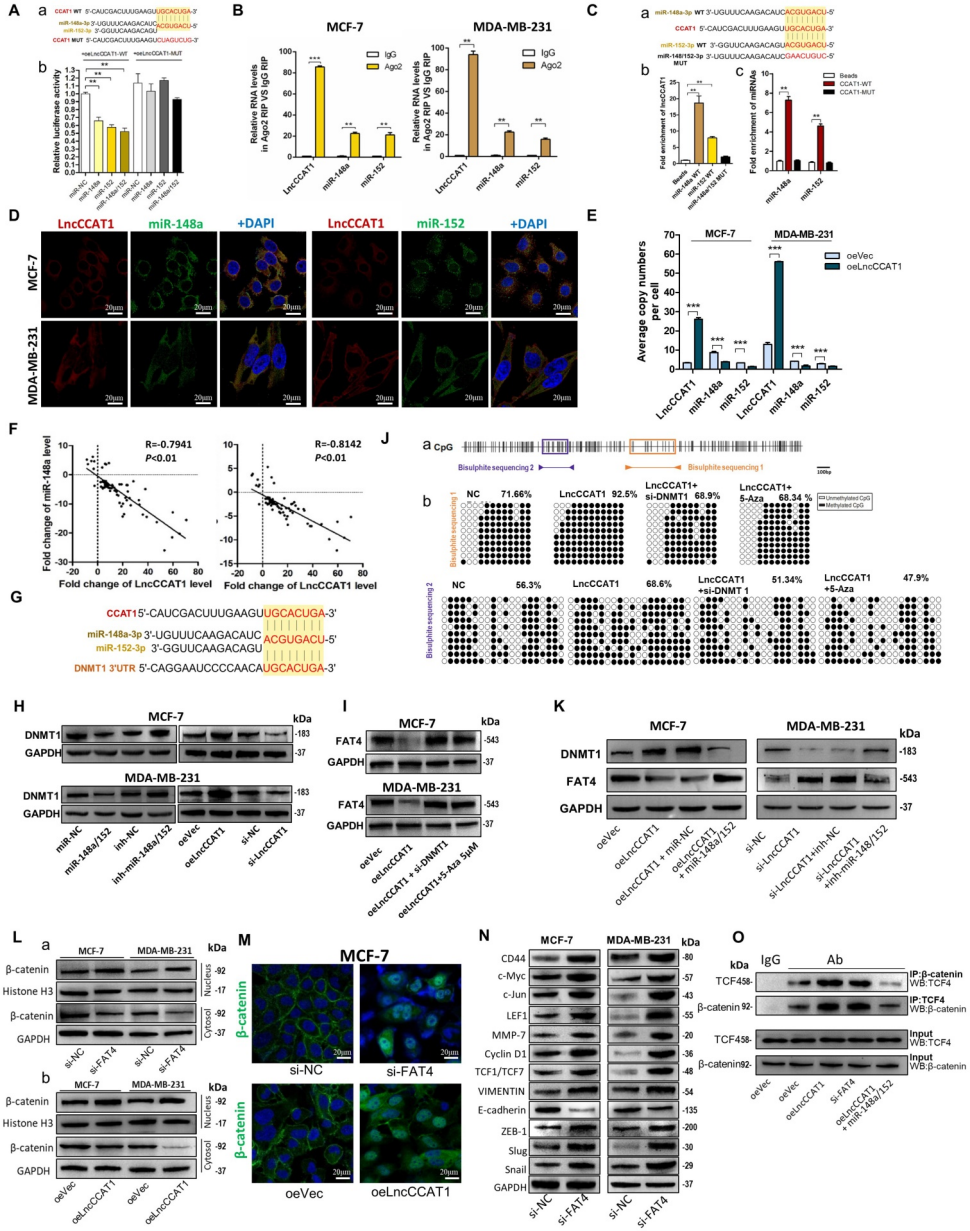
** LncCCAT1 facilitates β-catenin translocation into the nucleus by targeting miR-148a/152.** (**A**) Luciferase activity in MCF-7 cells co-transfected with a luciferase reporter containing LncCCAT1-WT or LncCCAT1-MUT (miR-148a/152-binding sequence mutated) and miR-148a/152 mimics (a). Data are presented as the relative ratio of renilla luciferase activity and firefly luciferase activity(b). (**B**) Relative enrichment of LncCCAT1, miR-148a, and miR-152 associated with AGO2 in MCF-7 and MDA-MB-231 cells detected by anti-AGO2 RIP (non-specific IgG as negative control). (**C**) Putative binding sequences between miR-148a/152 (WT) and LncCCAT1 in miR-148a/152 (WT) were mutated in miR-148a/152 (MUT) (a). RNA pulldown assays using biotin-labeled miR-148a or miR-152 (b) or biotin-labeled LncCCAT1 (c) in MCF-7 cells. (**D**) RNA-FISH assays indicated the colocalization of LncCCAT1 (red dots) and miR-148a (green dots) or miR-152 (green dots) in MCF-7 and MDA-MB-231 cells. Scale bar, 20 μm. (**E**) The exact copy numbers of LncCCAT1, miR-204 and miR-211 per cell in control and LncCCAT1-overexpressing MCF-7 and MDA-MB-231 cells. (**F**) The correlation between miR-148a or miR-152 and LncCCAT1in breast cancer tissues by Spearman correlation analysis. (**G**) The RNA algorithm predicted potential binding sites between miR-148a or miR-152 with LncCCAT1 or with DNMT1, with considerable sequence complementary in the indicated regions. (**H**) DNMT1 protein levels in different transfected MCF-7 and MDA-MB-231 cells as indicated. (**I**) FAT4 protein levels in different transfected or treated MCF-7 and MDA-MB-231 cells as indicated. (**J**) A schematic diagram of the CpG island and CpG sites in the FAT4 locus on human chromosome 4q28.1. Closed arrowheads and bars predicted the bisulfite sequenced regions (a). Bisulfite sequencing of the FAT4 CpG island in MCF-7 cells (b). Each box indicates the methylation status of the CpG site. Each row represents an individual sequenced DNA strand. The percentage of methylation in each sequenced region is indicated. (**K**) DNMT1 and FAT4 protein levels in different transfected MCF-7 and MDA-MB-231 cells as indicated. (**L, M**) Altered nuclear translocation of β-catenin in response to FAT4 knockdown (a) or LncCCAT1 overexpression (b) in MCF-7 and MDA-MB-231 cells analyzed by western blotting (Histone H3 or GAPDH as a control, respectively) (L) and in MCF-7 cells detected by IF(M). Scale bar, 20 μm. (**N**) The protein levels of Wnt/β-catenin signaling and EMT target genes in MCF-7 and MDA-MB-231 cells transfected with si-NC or si-FAT4. (**O**) Binding activities between β-catenin and TCF4 were detected by co-IP assays in different transfected MCF-7 cells as indicated. All data are shown as the mean±SD. *P<0.05, **P<0.01, and ***P<0.001 by two-tailed Student's t test.

We also investigated the interaction between LncCCAT1 and miR-148a/152. Luciferase reporter assays demonstrated that miR-148a/152 could bind to the MRE of LncCCAT1 (Figure 4A). Ago2 RIP assays showed that LncCCAT1 and miR-148a/152 were all obviously enriched in Ago2-IP (Figure 4B). Subsequent RNA pulldown experiments provided more solid evidence for the specific associations between miR-148a/152 and LncCCAT1 (Figure 4C). Moreover, RNA-FISH analysis suggested that LncCCAT1 colocalized with miR-148a/152 in the cytoplasm of MCF-7 and MDA-MB-231 cells (Figure 4D). These data revealed that LncCCAT1 can bind with miR-148a/152. We further compared the abundance of LncCCAT1 and miR-148a/152, which were also similar in MCF-7 and MDA-MB-231 cells (Figure 4E). LncCCAT1 overexpression also decreased the copy numbers of miR-148a/152 in these cells.

Next, we investigated whether a reciprocal negative correlation between LncCCAT1 and miR-148a/152 existed in breast cancer. Firstly, miR-148a/152 expression was mostly downregulated in breast cancer tissues compared with adjacent normal tissues (Figure S6A and B). Secondly, the expression levels of miR-148a/152 and LncCCAT1 were significantly negatively correlated in breast cancer tissues (Figure 4F) and cell lines (Figure S6C). Thirdly, LncCCAT1 overexpression significantly reduced miR-148a/152 expression, while LncCCAT1 knockdown increased miR-148a/152 levels. LncCCAT1-MUT had no obvious effect on miR-148a/152 expression (Figure S6D), suggesting that LncCCAT1 might induce miR-148a/152 degradation via direct binding to the MRE.

As LncCCAT1 shares a miR-148a/152 response element with DNA methyltransferase 1 (DNMT1), a known target of miR-148a/152 15, and participates in tumor progression by methylating and silencing tumor suppressor genes, we investigated whether LncCCAT1 could upregulate DNMT1 through sequestration of miR-148a/152. The predicted target sites interactions among LncCCAT1, miR-148a/152 and DNMT1 3'-UTR are illustrated in Figure 4G. miR-148a/152 overexpression reduced DNMT1 protein levels, while miR-148a/152 knockdown increased DNMT1 protein levels in two breast cancer cell lines, in contrast to the effect of LncCCAT1 overexpression or knockdown on DNMT1 protein (Figure S3D, Figure 4H).

We then studied which tumor suppressor gene could be suppressed by LncCCAT1 through upregulating DNMT1 in breast cancer by screening 14 genes, which were not only downregulated in LncCCAT1-overexpressing esophageal squamous cancer cells but can also be methylated in the promoter regions (MethPrimer, http://www.urogene.org/methprimer/index1.html). As shown in Figure S7A and B, FAT atypical cadherin 4 (FAT4) mRNA expression was downregulated by LncCCAT1 and rescued to normal levels after treatment with si-DNMT1 or 5-Aza-2'-deoxycytidine (5-Aza; a deoxycytidine analog used to activate methylated and silenced genes by promoter demethylation). We further verified that LncCCAT1 could decrease FAT4 protein level by upregulating DNMT1 (Figure 4I). To demonstrate that LncCCAT1-upregulated DNMT1 could inhibit FAT4 expression through methylation of its promoter, we assessed the extent of CpG methylation within the FAT4 promoter by bisulfite sequencing. We measured two distinct regions, named BSP1 and BSP2, within the CpG island of the FAT4 promoter (Figure 4J, a). BSP1 was found to have a higher degree (92.5%) of methylation in LncCCAT1-overexpressing cells than in control cells (71.66%), and DNMT1 downregulation or 5-Aza treatment recovered the methylation of the FAT4 promoter to 68.9% and 68.34%, respectively. Analysis of BSP2 displayed a similar trend in methylation status (Figure 4J, b). Moreover, promotion or impairment of LncCCAT1 on DNMT1 or FAT4 was recovered by overexpression of miR-148a/152 in MCF-7 cells, while a miR-148a/152 inhibitor could eliminate si-LncCCAT1-induced DNMT1 downregulation or FAT4 upregulation in MDA-MB-231 cells (Figure 4K). Together, our analysis strongly indicated that LncCCAT1 could downregulate FAT4 by DNMT1 by functioning as a ceRNA for miR148a/152.

FAT4 is a 543-kD protein and usually acts as a tumor suppressor 16; it was reported to be involved in Wnt/β-catenin signaling by modulating β-catenin nuclear translocation in gastric cancer 17. Therefore, we explored the effect of FAT4 on Wnt/β-catenin activation in breast cancer. We knocked down FAT4 in MCF-7 and MDA-MB-231 cells (Figure S7C and D) and found increased β-catenin levels in the nucleus concomitant with decreased β-catenin levels in the cytosolic fraction (Figure 4L, a). Similar results were observed upon LncCCAT1 overexpression (Figure 4L, b). Likewise, the immunofluorescence (IF) results demonstrated that both LncCCAT1 overexpression and FAT4 knockdown promoted β-catenin nuclear translocation (Figure 4M). In addition, FAT4 knockdown accelerated Wnt signaling and EMT activation (Figure 4N). Importantly, the coimmunoprecipitation (co-IP) assay demonstrated that LncCCAT1 overexpression and FAT4 knockdown promoted increased β-catenin translocation to the nucleus to bind with TCF4, while miR-148a/152 attenuated the LncCCAT1-mediated promotion (Figure 4O). Together, these data suggested that LncCCAT1 could act as a ceRNA for miR-148a/152 to elevate DNMT1 expression and then downregulates FAT4, which finally activated Wnt/β-catenin signaling.

### LncCCAT1 interacts with ANXA2 to activate Wnt/β-catenin signaling

**Figure 5:**
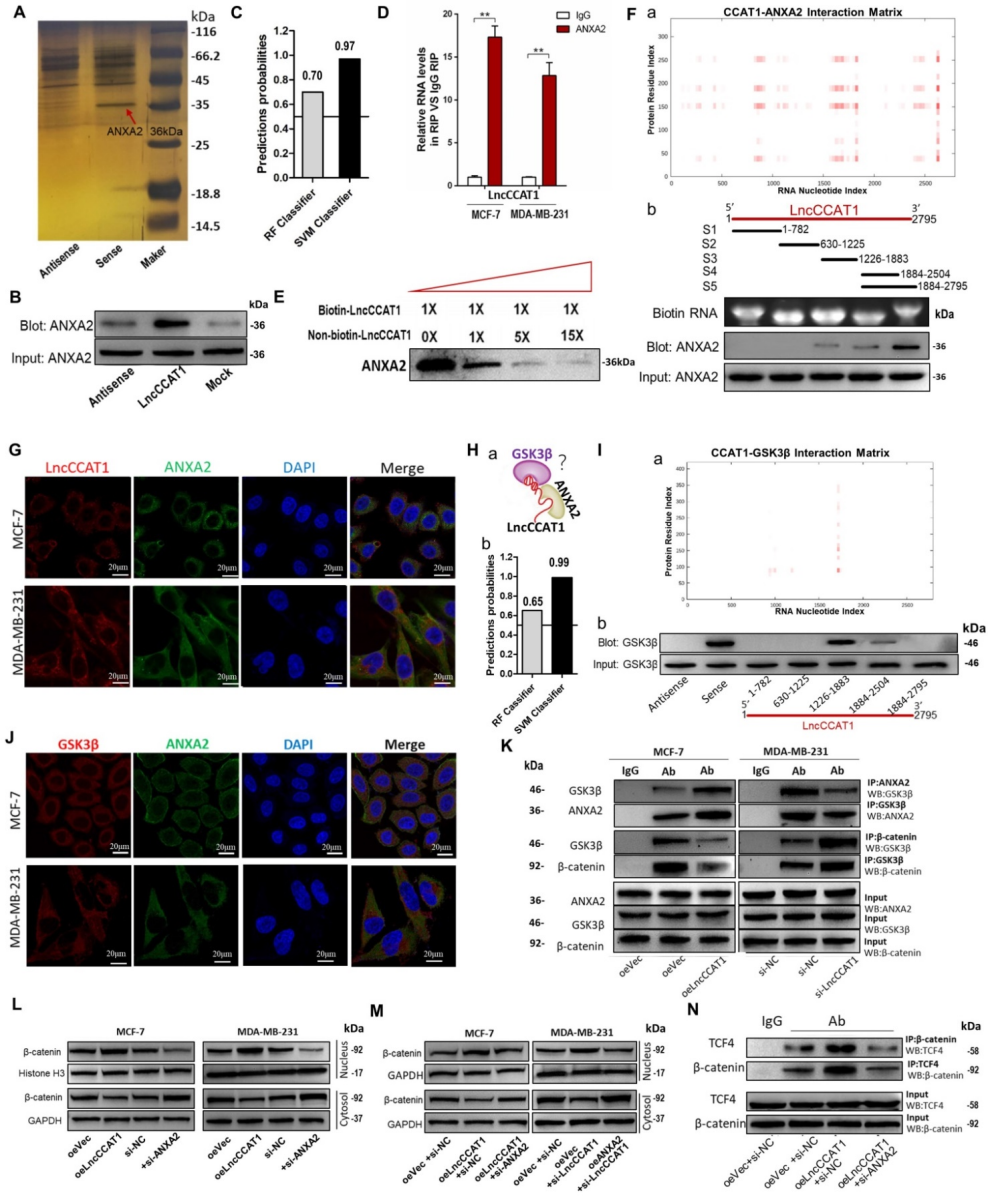
** LncCCAT1 interacts with ANXA2 to activate Wnt/β-catenin signaling.** (**A**) RNA pulldown assays were performed using biotin-labeled sense or antisense of LncCCAT1. Silver staining and mass spectrometry were performed to identify the interacting proteins. ANXA2 was identified, as indicated by the red arrow. (**B**) Proteins were pulled down by biotin-labeled sense or antisense LncCCAT1 and analyzed by western blotting with the ANXA2 antibody. (**C**) The possibility of interaction of LncCCAT1 and ANXA2 was predicted by RPISeq predictions (RF>0.5 and SVM>0.5 were considered positive). (**D**) The interaction between LncCCAT1 and ANXA2 was verified by RIP assays with ANXA2 antibody (non-specific IgG as a negative control). (**E**) The specific binding between LncCCAT1 and ANXA2 was confirmed by competition assays using biotin-labeled LncCCAT1 with increasing amounts of biotin-unlabeled LncCCAT1. (**F**) CatRAPID fragments module prediction of the interaction profile and matrix between ANXA2 protein and LncCCAT1(a). Mapping of ANXA2-binding domains of LncCCAT1(b). As shown: schematic diagram of LncCCAT1 full-length and truncated fragments (top); gel electrophoresis of *in vitro* transcribed biotin-labeled RNA of full-length and truncated LncCCAT1 (middle); western blotting of ANXA2 in RNA pulldown samples by different LncCCAT1 fragments (bottom). (**G**) Colocalization of LncCCAT1 (red) and ANXA2 (green) was visualized by FISH/IF assays. Scale bar, 20 μm.(**H**) Schematic diagram of the hypothesis that LncCCAT1 contributes to the interaction of ANXA2/GSK3β via binding with them in the adjacent domains(a). The possibility of interaction of LncCCAT1 and GSK3β was predicted by RPISeq predictions (b). (**I**) CatRAPID fragments module prediction of the interaction profile and matrix between GSK3β protein and LncCCAT1 (a). The core regions of LncCCAT1 required for physical interaction with GSK3β were identified by RNA pulldown assays with full-length LncCCAT1 and truncated fragments(b). (**J**) Colocalization of GSK3β (red) and ANXA2 (green) was visualized by FISH/IF assays. Scale bar, 20 μm. (**K**) Altered binding activities between GSK3β and ANXA2 and between GSK3β and β-catenin were detected by co-IP analyses of LncCCAT1-overexpressing MCF-7 and LncCCAT1-knockdown MDA-MB-231 cells. (**L**) Protein levels of β-catenin in the nucleus and cytosol in MCF-7 and MDA-MB-231 cells transfected with oeVec, oeANXA2, si-NC or si-ANXA2 detected by western blotting. (**M**) Protein levels of β-catenin in the nucleus and cytosol in different transfected MCF-7 cells and MDA-MB-231 cells as indicated. (**N**) Binding activities between β-catenin and TCF4 detected by co-IP assays in different transfected MCF-7 cells as indicated. All data are shown as the mean±SD. *P<0.05, **P<0.01, and ***P<0.001 by two-tailed Student's *t*-test.

LncRNAs could also function by binding to specific protein partners in cytoplasm^18^. Therefore, we performed an RNA pulldown assay with *in vitro-*transcribed biotin-labeled LncCCAT1 to search for potential LncCCAT1-associated proteins. As shown in Figure 5A and Figure S8A, mass spectrometry analysis of protein bands specific for LncCCAT1 revealed that ANXA2 bound to LncCCAT1 in MCF-7 cells. Moreover, ANXA2 was detected in the biotin-labeled sense LncCCAT1 group by RNA pulldown assays (Figure 5B). Then bioinformatics was used to predict the possibility of interaction between LncCCAT1 and ANXA2. RPISeq predictions (http://pridb.gdcb.iastate.edu/RPISeq/references.php) showed Random Forest (RF) was 0.7 and Support Vector Machine (SVM) was 0.97 (Figure 5C). RIP (Figure 5D) and competitive RNA pulldown assays (Figure 5E) further confirmed the interaction between ANXA2 and LncCCAT1. Next, catRAPID fragments, an algorithm based on individual interaction propensities of polypeptide and nucleotide sequence fragments, revealed that the 2586-2697 nucleotide positions of the LncCCAT1 sequence might bind to the ANXA2 protein with high propensities (Figure 5F, a) (http://service.tartaglialab.com/email_redir/123981/afc79d773e). We next constructed a series of LncCCAT1 truncations to map its binding fragment with ANXA2. Using an RNA pulldown assay, we found that the 3'-end fragment of LncCCAT1 (2504 to 2795 nt) was responsible for its interaction with ANXA2 (Figure 5F, b). We also observed that LncCCAT1 colocalized with ANXA2 in the cytoplasm of MCF-7 cells and MDA-MB-231 cells by RNA-FISH and IF (Figure 5G). However, LncCCAT1 did not influence the expression level of ANXA2 (Figure S8B).

ANXA2 interferes with multiple cellular processes and was reported to promote hepatocarcinogenesis by activating Wnt/β-catenin signaling 8. Accordingly, we first studied the functions of ANXA2 in breast cancer. As shown, ANXA2 overexpression could promote proliferation, sphere formation, migration and invasion of MCF-7 cells accompanied by activation of Wnt/β-catenin signaling and EMT process, while depletion of ANXA2 achieved the opposite effects in MDA-MB-231 cells (Figure S8C-G).

We next explored the function of the LncCCAT1/ANXA2 complex in breast cancer. ANXA2 was reported to prevent β-catenin phosphorylation and degradation by binding glycogen synthase kinase 3β (GSK3β) and disrupting the formation of the GSK3β/β-catenin complex in hepatocellular carcinoma cells 8. We noticed that GSK3β was one of the potential LncCCAT1-interacting protein candidates in MCF-7 cells detected by mass spectrometry analysis. Therefore, we speculated that LncCCAT1 might contribute to the interaction of ANXA2/GSK3β via binding with them (Figure 5H, a). RPISeq predictions showed RF and SVM of LncCCAT1- GSK3β were 0.65 and 0.99 (Figure 5H, b). CatRAPID fragments revealed that the 1695-1806 nucleotide positions of the LncCCAT1 sequence might bind to the GSK3β protein with high propensities (Figure 5I, a) (http://service.tartaglialab.com/email_redir/191623/e7e09cc3c6). Analysis of LncCCAT1 truncations indicated that GSK3β might bind to the 1266 to 1883 nt region of LncCCAT1 (Figure 5I, b). We further investigated the relationships among LncCCAT1, ANXA2, GSK3β and β-catenin. RNA-FISH assays showed that ANXA2 colocalized with GSK3β in the cytoplasm of MCF-7 and MDA-MB-231 cells (Figure 5J). Co-IP assays demonstrated that ANXA2 could bind GSK3β, and LncCCAT1 overexpression significantly enhanced the ANXA2/GSK3β interactions, thus repressing the binding between GSK3β/β-catenin in MCF-7 cells, whereas LncCCAT1 depletion in MDA-MB-231 cells generated the opposite effects (Figure 5K).

Moreover, we found that ANXA2 could alter the nuclear accumulation of β-catenin and the activation of Wnt/β-catenin signaling and EMT, similar to LncCCAT1 (Figure 5L). Meanwhile, ANXA2 knockdown or overexpression repressed the effect of LncCCAT1 overexpression or knockdown on β-catenin nuclear translocation, respectively (Figure 5M). LncCCAT1 overexpression enhanced the binding activity between TCF4/β-catenin, while ANXA2 knockdown attenuated this effect (Figure 5N). The above data indicated that LncCCAT1 contributes to the interaction of ANXA2/GSK3β via binding with them in the adjacent domains, and LncCCAT1-ANXA2-GSK3β interaction may play an important role in Wnt/β-catenin signaling and EMT.

### LncCCAT1 regulates breast cancer cell functions *in vitro* by binding miR-204/211, miR-148a/152, and ANXA2 to activate Wnt signaling

**Figure 6:**
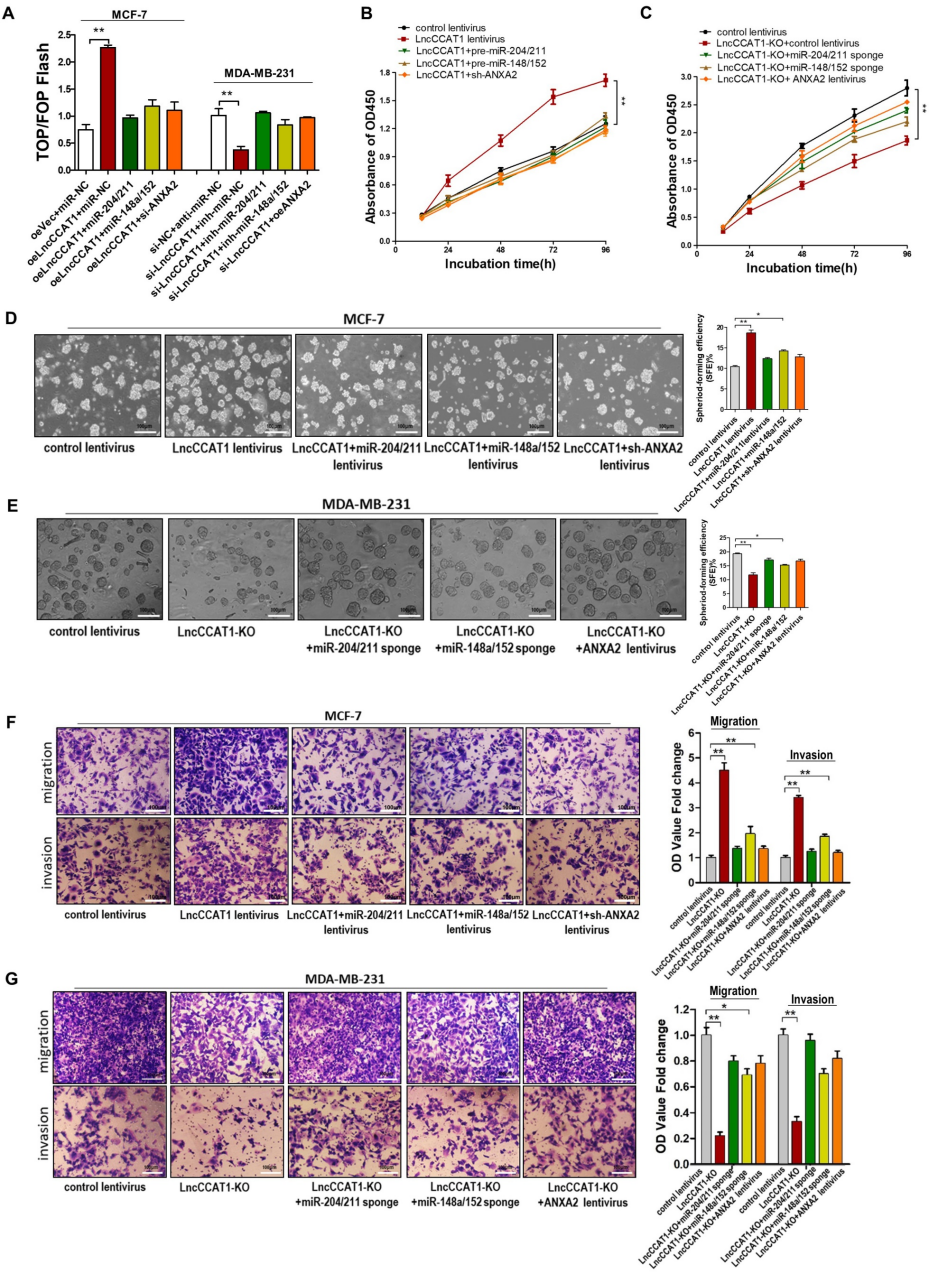
** Overexpression of miR-204/211 or miR-148a/152 or knockdown of ANXA2 restores the pro-proliferation, pro-self-renewal, pro-migration, and pro-invasion functions of LncCCAT1 in breast cancer cells *in vitro*.** (**A**) The activity of the WNT/β-catenin pathway was determined using a TOP/FOP luciferase reporter plasmid, and the expression was normalized to renilla activity. (**B, C**) Proliferative abilities of MCF-7 cells transfected with lentivirus as indicated (B) and MDA-MB-231 cells or LncCCTA1-KO MDA-MB-231 cells transfected with lentivirus as indicated (C) detected by CCK-8 assays. (**D, E**) Sphere-formation abilities of MCF-7 cells transfected with lentivirus as indicated (D) and MDA-MB-231 cells or LncCCTA1-KO MDA-MB-231 cells transfected with lentivirus as indicated (E). SFEs are shown on the right. Scale bar, 100 μm. (**F, G**) Migration and invasion abilities of MCF-7 cells transfected with lentivirus as indicated (F) and MDA-MB-231 cells or LncCCTA1-KO MDA-MB-231 cells transfected with lentivirus as indicated (G) detected by transwell assays. Scale bar, 100 μm. All data are shown as the mean±SD. *P<0.05, **P<0.01, and ***P<0.001 by two-tailed Student's *t*-test.

To further verify that the contribution of LncCCAT1 to BCSC functions was mediated by binding miR-204/211, miR-148a/152, and ANXA2 to activate Wnt signaling, we performed gain- and loss-of-function assays. The TOP/FOP-Flash luciferase reporter system was used to examine Wnt signaling activity. We observed that enhanced Wnt activity in LncCCAT1-overexpressing cells was attenuated by miR-204/211 or 148a/152 overexpression or ANXA2 knockdown. In contrast, reduced luciferase activity in LncCCAT1-KO cells was reversed by suppressing miR-204/211 and miR-148a/152 or upregulating ANXA2 (Figure 6A). Also, overexpression of miR-204/211, miR-148a/152 or knockdown of ANXA2 abolished the enhanced proliferative, sphere-formation, migration and invasion capacities by LncCCAT1 overexpression. Meanwhile, inhibition of miR-204/211 or miR-148a/152 or overexpression of ANXA2 rescued the reduction in proliferative, sphere-formation, migratory and invasive capacities caused by LncCCAT1-KO (Figure 6B-G). These findings suggested that LncCCAT1 could promote the proliferation, stemness, migration and invasion of breast cancer cell through binding to miR-204/211, miR-148a/152, and ANXA2, which leading to the activation of Wnt signaling.

### LncCCAT1 promotes breast tumor growth and metastasis *in vivo* by binding miR-204/211, miR-148a/152 and ANXA2

**Figure 7:**
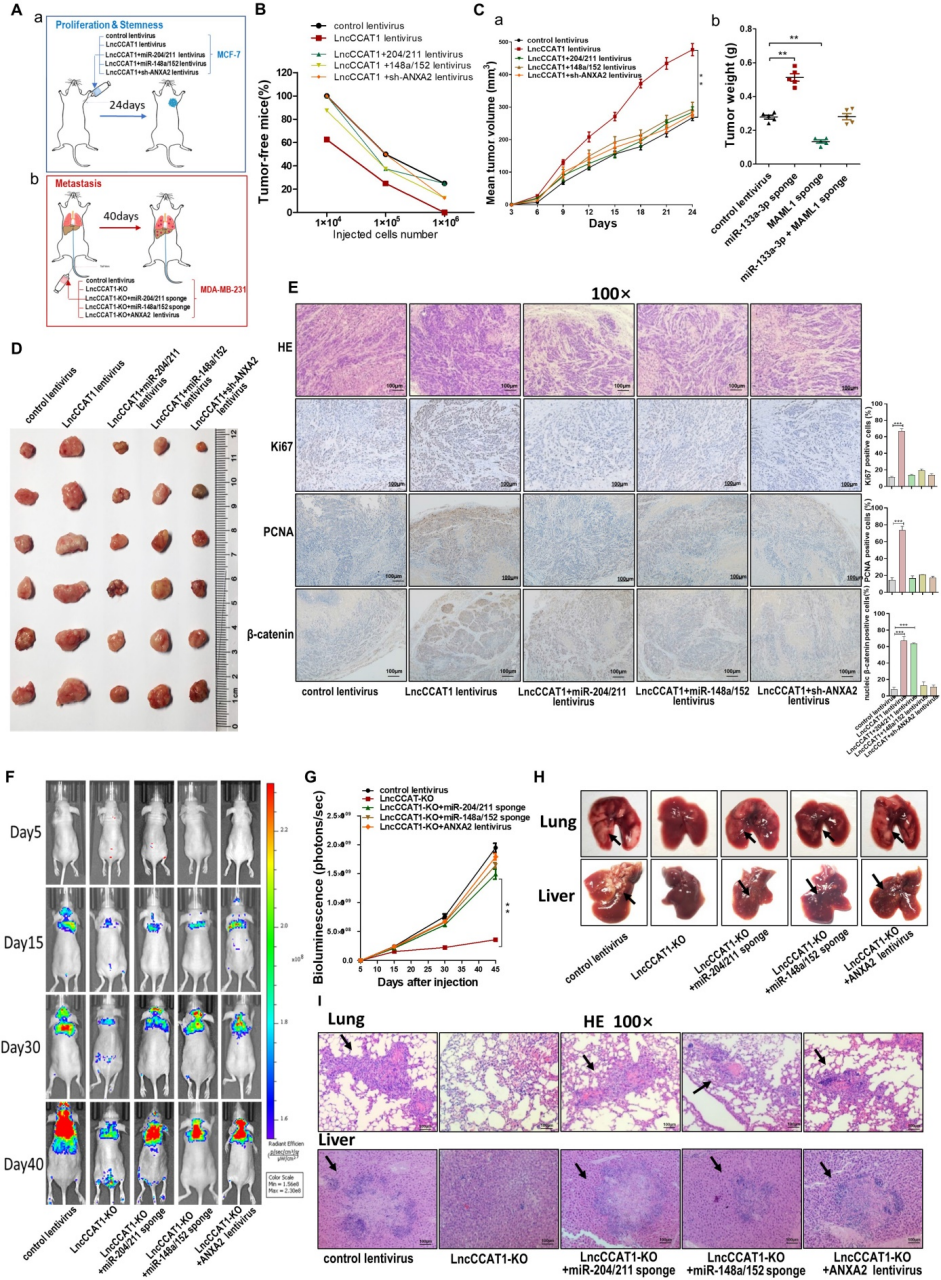
** LncCCAT1 promotes breast cancer growth and metastasis *in vivo* by binding to miR-204/211, miR-148a/152, and ANXA2.** (**A**) Flowchart of the *in vivo* xenograft experiments designed for detecting tumor proliferation and stemness (a), and metastasis (b). (**B**) *In vivo* limiting dilution assay of indicated MCF-7 cells was adopted to evaluate the effect of LncCCAT1 on the tumor-initiating frequency. Tumors were observed over 1 month; n=8 for each group. (**C, D**) The tumor growth of mice subcutaneously implanted with the indicated MCF-7 cells. Tumor volume (C, a) and weight (C, b) were measured, and tumor size is pictured (D). (**E**) Representative H&E-stained sections and immunohistochemical staining of Ki-67, PCNA and β-catenin of the tumors from subcutaneously implanted mice. Scale bar, 100 μm. (**F, G**) Representative BLI images (F) and quantitative analysis of the fluorescence intensities (G) of mice intravenously injected with the indicated MDA-MB-231 cells on days 5, 15, 30, and 40 after injection. (**H, I**) The gross lesions (H) and representative H&E-stained sections (I) of lung tissues and liver tissues isolated from the intravenously injected mice. Black arrows indicate metastatic nodules. Scale bar, 100 μm. All data are shown as the mean±SD. *P<0.05, **P<0.01, and ***P<0.001 by two-tailed Student's *t*-test.

Finally, we confirmed the oncogenic role of LncCCAT1 in breast cancer *in vivo*. To test the pro-proliferative and -stemness effects of LncCCAT1, we subcutaneously implanted different stable MCF-7-expressing cell lines into nude mice via the armpit (Figure 7A, a). To evaluate the effect of LncCCAT1 on tumor metastasis, we intravenously injected different stable MDA-MB-231-expressing cell lines into nude mice via the tail vein (Figure 7A, b). *In vivo* limiting dilution assay revealed that LncCCAT1 overexpression in MCF-7 cells significantly increased tumor incidence, while miR-204/211, miR-148a/152, or sh-ANXA2 lentivirus abrogated the LncCCAT1-enhanced tumorigenicity of MCF-7 cells *in vivo* (Figure 7B, Figure S9A-C). Moreover, in the xenograft model, miR-204/211, miR-148a/152 and sh-ANXA2 suppressed the tumor growth enhanced by LncCCAT1 overexpression (Figure 7C-D). H&E staining showed that the histopathology of tumors formed by the LncCCAT1-overexpressing MCF-7 cells exhibited morphologic characteristics of poorly differentiated carcinoma and increased cell mitosis, whereas miR-204/211, miR-148a/152 and sh-ANXA2 attenuated these effects and showed morphological characteristics of highly differentiated carcinoma with low malignancy (Figure 7E). Ki-67 and PCNA immunohistochemistry showed that miR-204/211, miR-148a/152 and sh-ANXA2 attenuated the tumor cell growth induced by LncCCAT1 (Figure 7E). β-catenin immunohistochemistry revealed that LncCCAT1-promoted β-catenin nuclear translocation was abolished by miR-148a/152 and sh-ANXA2. Furthermore, LncCCAT1-overexpressed MCF-7 cells exhibited higher expression of pluripotent transcription factors (Figure S9D) and Wnt target genes (Figure S10) compared with the control cells in the xenografts, whereas miR-204/211, miR-148a/152 and sh-ANXA2 attenuated these effects.

Bioluminescence images (BLI) were taken at intervals from day 5 to day 40 post-tail vein injection. The analysis of the BLI scans revealed that LncCCAT1-KO in MDA-MB-231 cells caused little lung and liver tumor metastasis. However, miR-204/211 or miR-148a/152 knockdown or ANXA2 overexpression reversed the suppression of metastatic foci formation caused by LncCCAT1-KO (Figure 7F and G). We also observed that the metastatic lesions at the surface of the lungs and livers were reduced in the LncCCAT1-KO group compared with the control group, while miR-204/211 or miR-148a/152 knockdown or ANXA2 overexpression in LncCCAT1-KO cells recovered the levels to those of the control cells (Figure 7H). H&E staining showed that LncCCAT1-KO significantly reduced metastatic nodules in the lungs and livers of mice, which was rescued to control levels by miR-204/211 or miR-148a/152 knockdown or ANXA2 overexpression (Figure 7I). All of the above data supported that LncCCAT1 could promote breast tumor growth, stemness and metastasis through interaction with miR-204/211, miR-148a/152 and ANXA2.

## Discussion

In the present study, we elucidated the critical role of LncCCAT1 in BCSCs and the underlying mechanisms. We showed that LncCCAT1 was highly expressed in breast cancer tissues and BCSCs. Moreover, the high expression of LncCCAT1 in breast cancer patients was positively correlated with advanced tumor grade, TNM stage, positive lymph node metastasis and poor prognosis. We further found that LncCCAT1 was functionally required for BCSC stemness maintenance, proliferation, migration and invasion. Mechanistically, LncCCAT1 enhanced TCF4 levels by competitively binding to miR-204/211 and facilitated β-catenin nuclear translocation by interacting with miR-148a/152 and ANXA2, resulting in the activation of Wnt/β-catenin signaling, EMT, and LncCCAT1 transcription, thereby promoting breast cancer carcinogenesis.

More than 20 lncRNAs have been clearly investigated in breast cancer progression or CSC functions 19, 20. LncRNA-Hh strengthened BCSC generation in twist-positive breast cancer via activation of the hedgehog signaling pathway 21. LncRNA H19 promoted BCSC maintenance by forming a double-negative feedback loop with the miRNA let-7 and the transcription factor LIN28 22. However, the regulatory roles of lncRNAs in CSCs, especially in BCSCs, remain largely unknown. In this study, we identified 19 intersecting lncRNAs that were differentially expressed in both poorly differentiated breast cancer tissues and BCSCs. Among these, LncCCAT1 was highly expressed and was therefore further verified in 80 breast cancer tissues and MCF-7 and MDA-MB-231 BCSCs. Moreover, overexpression of LncCCAT1 in breast cancer tissues was associated with a poor prognosis and could be an independent prognostic indicator.

LncCCAT1 maps to chromosome 8q24.21, which was first found to be upregulated in colon cancer 23. In recent years, LncCCAT1 was reported to be upregulated and to exhibit oncogenic properties in various cancer types 24, 25. C-Myc-activated LncCCAT1 can promote proliferation and invasion of colon cancer cells, gastric cancer cells, hepatoma cells and pancreatic cancer cells 26, 27 and reduce cell radiosensitivity by regulating miR-148b in breast cancer cells 28. Study also showed that LncCCAT1 can stimulate the symmetric division of non-small-cell lung CSCs 29. However, the biological functions of LncCCAT1 in the control of BCSCs remain unclear. Our study provides the first evidence that LncCCAT1 promotes BCSC proliferation, stemness, migration and invasion, indicating that LncCCAT1 might play critical roles in breast cancer progression by regulating BCSC functions.

Cytoplasmic lncRNAs, which act as ceRNAs for miRNAs, base-pair with mRNAs, and interact with proteins, have been studied extensively^30^. In this study, we found that LncCCAT1 was mainly localized in the cytoplasm and that endogenous miR-204/211, miR-148a/152, and ANXA2 protein were associated with LncCCAT1 in breast cancer cells. The possibility of lncRNA-induced miRNA degradation in cancer cells has been reported elsewhere 31, but the underlying mechanism is still elusive. For example, LncCCAT1 overexpression reduced miR-7 expression in esophageal squamous carcinoma cells and decreased miR-218 expression in gallbladder cancer cells 25. Herein, we observed that LncCCAT1 could bind miR-204/211/148a/152 and repress their expression, leading to the upregulation of their targets, TCF4 and DNMT1. As LncCCAT1 and miR-204/211/148a/152 are all associate with Ago2, the core component of the RNA-induced silencing complex (RISC), the regulation between LncCCAT1 and miR-204/211/148a/152 might be similar to the miRNA-mediated silencing of protein-coding genes. Furthermore, we demonstrated that upregulation of TCF4 directly initiated the Wnt signaling pathway and thereby induced EMT, while DNMT1 indirectly activated Wnt signaling by enhancing TCF4/β-catenin binding activity through downregulating FAT4. DNMT1 was recently found to be involved in pancreatic CSC regulation via upregulation of the miR-17-92 cluster 32. Herein, we demonstrated that LncCCAT1-increased DNMT1 could activate Wnt signaling in BCSCs by inhibiting FAT4 expression through methylation of its promoter. FAT4 was reported to modulate the nuclear translocation of β-catenin to facilitate the transcription of Wnt target genes in gastric cancer 33, but the underlying mechanism was unclear. Due to the fact that FAT1 could bind with β-catenin and antagonize its nuclear translocation^34^, we hypothesized that FAT4 might share a similar mechanism with FAT1. Intriguingly, ANXA2, which was also related to Wnt/β-catenin activation, was demonstrated to abundantly bind with LncCCAT1. By binding to GSK3β, ANXA2 can disrupt the formation of the GSK3β/β-catenin complex, which leads to reduced β-catenin phosphorylation and degradation 8. Cytoplasmic lncRNAs could bind with proteins to regulate their expression 35, influence their stability 36 or harbor members of the protein complex 37. In the present study, we found that LncCCAT1 could act as a modular scaffold to recruit ANXA2 and GSK3β, thus upregulating β-catenin and leading to increased β-catenin translocation to the nucleus to interact with TCF4.

All of the above approaches triggered the activation of the Wnt/β-catenin signaling pathway, which is not only implicated in tumor progression but also plays a pivotal role in CSC functions 38. Rescue experiments performed *in vitro* and *in vivo* further demonstrated that LncCCAT1 facilitated breast tumor growth and metastasis through activation of the Wnt/β-catenin signaling pathway by binding with miR-204/211, miR-148a/152 and ANXA2. However, the data showed that the effect of miR-148a/152 was weaker than miR-204/211 and ANXA2. This finding might be due to the indirect regulatory axis and the different background expression levels among miR-204/211, miR-148a/152 and ANXA2. Interestingly, the transcription of LncCCAT1 itself is directly activated by TCF4 and therefore might enhance the duration and intensity of Wnt signaling through a positive feedback loop.

## Conclusions

In summary, our study highlights the importance of LncCCAT1 as a mediator of BCSC-modulated breast cancer progression. Our data suggest that LncCCAT1 may be a potential biomarker and a therapeutic target for breast cancer patients.

## Supplementary Material

Supplementary methods, figures and tables.

## Abbreviations

BCSCs: Breast cancer stem cells
LncCCAT1: long noncoding RNA cancer-associated transcript-1
ANXA2: Annexin A2
TCF4: T-cell factor 4
Wnt: wingless/integrated
CSCs: cancer stem cells
EMT: epithelial-to-mesenchymal transition
LncRNAs: Long noncoding RNAs
STR: short tandem repeat
RIP: RNA immunoprecipitation
DAB: diaminobenzidine
GEO: Gene Expression Omnibus
FISH: fluorescence *in situ* hybridization
ceRNA: competing endogenous RNAs
MS2-RIP: MS2 RNA immunoprecipitation
MREs: miRNA response elements
Ago2-IP: Ago2-immunoprecipitation
UTR: untranslated region
DBEs: DNA binding elements
ChIP: chromatin immunoprecipitation
DNMT1: DNA methyltransferase 1
FAT4: FAT atypical cadherin 4
5-Aza: 5-Aza-2'-deoxycytidine
Co-IP: coimmunoprecipitation
RF: Random Forest
SVM: Support Vector Machine
GSK3β: glycogen synthase kinase 3β
BLI: Bioluminescence images
RISC: RNA-induced silencing complex
