# Supplementary material for: LncCCAT1 Promotes Breast Cancer Stem Cell Function through Activating WNT/β-catenin Signaling
Source: Theranostics. 2019 Oct 1;9(24):7384–402. doi: 10.7150/thno.37892 (PMC6831302; doi:10.7150/thno.37892)
Supplement: Supplementary file 1 — Supplementary methods, figures and tables. [file thnov09p7384s1.pdf]

Supplementary Files

LncCCAT1 Promotes Breast Cancer Stem Cell Function Through Activating WNT/β-catenin Signaling

Tingting Tang [1], Changying Guo[1], Tiansong Xia[3], Rui Zhang[4], Ke Zen[2], Yi Pan [1, *], Liang Jin[1, *]

Supplementary Data list

Supplementary Materials and Methods

Supplementary Figure and Table legends

Supplementary Figures:

Supplementary Figure S1

Supplementary Figure S2

Supplementary Figure S3

Supplementary Figure S4

Supplementary Figure S5

Supplementary Figure S6

Supplementary Figure S7

Supplementary Figure S8

Supplementary Figure S9

Supplementary Figure S10

Supplementary Spreadsheets:

Supplementary Table S1

Supplementary Table S2

Supplementary Table S3

Supplementary Table S4

Gel

Cell lines authentication report

Supplementary Materials and Methods

RNA isolation and qRT-PCR analysis

Total RNA was isolated using TRIzol (Invitrogen) and a RNeasy kit (QIAGEN, Duesseldorf, Germany) with DNase I digestion according to the manufacturers' instructions. Reverse transcription reaction was performed using PrimeScriptTM RT reagent Kit (Takara, Tokyo, Japan) and diluted cDNA was used for qRT-PCR analysis using SYBR Premix Ex Taq II Kit (Takara) with the appropriate primers listed in Supplementary Table S4. For microRNA, stem-loop reverse transcription reactions were performed and the $2^{-\triangle Ct}$ method was used to calculate the relative abundance of RNA genes compared with GAPDH or U6 expression.

Flow cytometry and cell sorting

Cells were stained using different antibodies according to the manufacturer's instructions. Labeled cells were detected using a FACSCalibur (BD Immunocytometry Systems, San Jose, CA). For cell sorting, cocktail PE-conjugated anti-human CD24, APC-conjugated anti-human CD44 and PE/Cy7-conjugated CD326 antibodies were incubated with MCF-7 cells or MDA-MB-231 cells, followed by sorting with FACS Aria III (BD). Antibodies used are provided in Supplementary Table S5.

In vitro Transcription Assays and RNA pull-down assays

RNA pull-down was performed as previously described. Briefly, in vitro translation assays were performed using T7 RNA Polymerase transcription kit according to the manufacturer instruction (Thermo, MA, USA), total RNA treated with RNase-free DNase I (Roche, Basel, Switzerland) and then purified with RNeasy Mini Kit (QIAGEN). Then LncCCAT1 RNAs were labeled by Desthiobiotinylation by using Pierce RNA 3 End Desthiobiotinylation Kit (Thermo). Biotinylated RNAs were incubated with cytoplasmic extract of MCF-7 cells at room temperature for 1h. Washed streptavidin agarose beads (Invitrogen) were added to each binding reaction and incubated at room temperature for 1h. Precipitates were washed for three times, boiled in SDS buffer and subjected to SDS–polyacrylamide gel electrophoresis. Specific bands were excised and analyzed by mass spectrometry or subjected to western blotting.

Bisulphite sequencing.

Genomic DNA was extracted from breast cancer cells and subjected to sodium bisulphite modification using EpiTect Bisulfite Kits (QIAGEN) following the manufacturer's protocols. Sodium bisulphite-treated DNA was

PCR amplified using primers predicted on MethPrimer website and EpiTaq™HS (Takara) was used for bisulphite PCR. The PCR products in each group were cloned into the T-Vector pMD19 (Takara), and over 10 clones were sequenced.

Cytosolic/nuclear fractionation.

The separation of the nuclear and cytosolic fractions was performed using the PARIS Kit (Life Technologies, CA, USA) according to the manufacturer's instructions. Cells were incubated with hypotonic buffer (25mM Tris–HCl, pH 7.4, 1mM $MgCl_2$ and 5mM KCl) on ice for 5min. An equal volume of hypotonic buffer containing 1% NP-40 was then added, and the sample was left on ice for another 5min. After centrifugation at 5,000g for 5min, the supernatant was collected as the cytosolic fraction. The pellets were resuspended in nuclear resuspension buffer (20 mM HEPES, pH 7.9), 400 mM NaCl, 1 mM EDTA, 1 mM EGTA, 1mM dithiothreitol and 1mM phenylmethyl sulfonyl fluoride and incubated at 4 °C for 30min. The nuclear fraction was collected after removal of insoluble membrane debris by centrifugation at 12,000g for 10min.

Western blotting analysis

Cells proteins were separated by SDS-PAGE electrophoresis and then transferred to a polyvinylidene difluoride membrane. Membranes were immunoblotted with primary antibodies and detected with horseradish peroxidase-conjugated anti-IgG. Then membranes were visualized with an enhanced chemiluminescence kit (Tanon, Shanghai, China). Antibodies used in the western blotting are provided in Supplementary Table S5.

Plasmid construction

LncCCAT1, ANXA2 and TCF4 cDNA were amplified by using Super-Fidelity DNA Polymerase (Vazyme, Nanjing, China) and cloned into the expression vector pcDNA3.1 (Invitrogen). The five LncCCAT1 fragments for RNA pull down assays used pcDNA3.1-CCAT1 as a template for generating constructs carrying deletions using respective primers and named S1-S5. The pcDNA3.1-CCAT1 with point mutations in the miR-148a/152 and miR-204/211 response elements was synthesized by GeneCreate (Wuhan, China) and named pcDNA3.1-CCAT1-MUT. pcDNA3.1-MS2-12X (Addgene) was double digested with EcoR I and Xho I, and the MS2-12X fragment was subcloned into pcDNA3.1, pcDNA3.1-CCAT1 and pcDNA3.1-CCAT1-MUT, named pcDNA3.1-MS2, pcDNA3.1-MS2-CCAT1 and pcDNA3.1-MS2-CCAT1-MUT respectively. For Luciferase reporter assay, the 59-nt region of either LncCCAT1, LncCCAT1-MUT (miR-148a/152) and LncCCAT1-MUT (miR-204/211)

was synthesized and subcloned into the pmirGLO vector (Promega, Madison, WI). The 3’untranslated regions (3’-UTR) of TCF4 mRNA containing the intact miR-204/211 recognition sequences were PCR-amplified and subcloned into pmirGLO vector. All PCR products were verified by DNA sequencing. Primers used for plasmid construction are listed in Supplementary Table S4.

Knockdown by siRNAs

siRNAs targeting FAT4, DNMT1, ANXA2, TCF4 were designed and synthesized by Ribo (Guangzhou, China). For transient knockdown LncCCAT1, RiboTM LncRNA Smart Silencer (Ribo) was used, which is a pool containing three siRNA and three antisense oligonucleotides (Supplementary Table S6).

Proliferation, migration and Invasion assay

The capacity of cellular proliferation was measured using the Cell Counting Kit-8 (CCK-8) (Dojindo Laboratories, Kumamoto, Japan) and EdU Cell Proliferation Assay Kit (Ribobio, Guangzhou, China) according to the manufacturer’s instructions. The optical density was determined with a microplate reader at a wavelength of 450 nm. To analyze the migration and invasion abilities of cells, transwell migration assay and transwell invasion assay was performed based on published method. For the cell transmembrane migration assay, all the steps were carried out similarly to those in the invasion assay except for the Matrigel (BD Biosciences, San Jose, CA, USA) coating. After incubation at 37˚C for 24 h, the filters were removed. The cells adhering to the lower surface were fixed and stained with Crystal Violet, 10 randomly selected fields in each well were counted. n=3, Magnification was 200×.

Luciferase reporter assay

pmirGLO, pmirGLO-CCAT-WT or pmirGLO-CCAT-MUT (miR-148a/152, miR-204/211) was co-transfected with pre miR-148a/152, pre miR-204/211 mimics or miR-NC into MCF-7 cells by Lipofectamine-mediated gene transfer. pmirGLO, pmirGLO-TCF4-WT or pmirGLO-TCF4-MUT was transfected into MCF-7 cells by lipofectamine-mediated gene transfer. The relative luciferase activity was normalized to Renilla luciferase activity 48h after transfection.

Cell transfection and Lentivirus production

Transfections were performed using the Lipofectamine 2000 kit (Invitrogen) according to the manufacturer’s

instructions. The double-stranded microRNA mimics and their respective negative control RNAs (GenePharma, Shanghai, China) were transfected into cells at a final concentration of 50 nM. The cells were harvested at 48h after transfection. Recombinant lentiviruses containing pre-hsa-miR-148a, pre-hsa-miR-204, hsa-miR-148a inhibitor, hsa-miR-152 inhibitor or the control were purchased from (GenePharma). For stable overexpression, the cDNA of LncCCAT1, TCF4 and ANXA2 genes were cloned into the lentiviral expression vector pLVX-IRES-ZsGreen1 (Addgene). To produce lentivirus, HEK-293T cells were co-transfected with the lentiviral vector and packaging vectors psPAX2 and pMD2.G using Lipofectamine 2000 (Invitrogen) according to the manufacturer's guidelines. Infectious lentiviruses were harvested at 48h post transfection and filtered through 0.45μm PVDF filters and the recombinant lentiviruses were concentrated 100-fold by ultracentrifugation (3 h at 50,000 g). The virus-containing pellet was dissolved in DMEM, aliquoted and stored at -80°C. Based on the shRNA sequences provided above, the most effective shRNAs were used for subsequent studies. shRNAs against ANXA2, TCF4 and control hairpins were cloned into pLVX-shRNA2 vector as described above, the virus supernatant was collected and cells were transfected with lentiviral constructs expressing as described above for 24h. The expression of LncCCAT1, ANXA2 and TCF4 in the infected cells was collected for protein and RNA analysis 96h after infection.

Fluorescence *in situ* hybridization (FISH) and LNA based *in situ* hybridization (LNA ISH)

Cy3 labeled LncCCAT1 probe was designed and synthesised by (Ribo, Guangzhou, China). For FISH assay, cells were fixed in 4% formaldehyde and permeabilized with 0.5% Triton X-100 for 5 min, washed with PBS three times and once in 2× SSC buffer. Hybridization was carried out using DNA probe sets at 37°C for 12 h. Images were obtained with confocal laser microscope (Carl Zeiss, Oberkochen, Germany). In situ hybridization (ISH) was performed by applying the ISH Kit (Boster, Bio-Engineering Company, Wuhan, China). Formalin-fixed paraffin embedded (FFPE) tissue slides were deparaffinized and deproteinated. Slides were then prehybridized in prehybridization solution for 2 h at 42 °C and incubated in DIG-labeled probe solution over night at 42°C. After stringent washing, the slides were exposed to a streptavidin-peroxidase reaction system and stained with 3, 3′diaminobenzidine (DAB, ZSGB-BIO, Beijing, China). Hematoxylin (Sigma) was used to counterstain the slides.

The copy number of LncCCAT1 and miRNAs per cell

The standard curves were formulated with limit dilution approaches using LncCCAT1 expressing vector

pcDNA3.1-LncCCAT1 and reverse transcribed miR-204/211/148a/152 cDNA as standard templates, and then the exact copy numbers of LncCCAT1 and miR-204/211/148a/152 per cell were calculated according to cell counts and molecular weights.

TOP-Flash reporter assay

Cells were transfected with 200 ng of the TOP-FLASH or FOP-FLASH reporter constructs together with 20 ng of the Renilla luciferase vector. Luciferase activity was measured by the Dual–Luciferase Reporter Assay System (Promega) 48 h after transfection, and TOP or FOP values were normalized to Renilla values. The TOP/FOP ratios were calculated and used as indicators of the endogenous level of WNT signaling. The experiment was repeated twice and the result was reproducible.

Supplementary Figure and Table legends

Supplementary Figure S1. Verification of LncCCAT1 overexpression, KD and KO efficiencies of oeLncCCAT1, si-LncCCAT1 and CRISPR/Cas9, respectively. (**A**) TCGA database indicated that LncCCAT1 was correlated with a poor survival rate in patients with Luminal A breast cancer (Kaplan-Meier survival analysis). (**B, C**) LncCCAT1 overexpression efficiencies by transfecting LncCCAT1 plasmid (B) and LncCCAT1 lentivirus (C) in MCF-7 and MDA-MB-231 cells detected by qRT-PCR. (**D**) LncCCAT1 knockdown efficiency after transfection of MCF-7 and MDA-MB-231 cells with LncCCAT1 siRNAs. (**E-H**) Generation of LncCCAT1-KO breast cancer cells by CRISPR/Cas9. Knockout strategy with gRNA sequences (gRNA-1), and relative positions of primers (F1/R1) used for genomic PCR and primers (F2/R2) used for detecting deletions (E). Identification of KO clones by genomic PCR (F). Detection of LncCCAT1 in KO clones by qRT-PCR (G). Nucleotide sequences of three clones involving deletions (H). *P<0.05, **P<0.01, and ***P<0.001 by two-tailed Student's *t*-test.

Supplementary Figure S2. LncCCAT1 promotes breast cancer cell stemness and migration *in vitro*.

(**A**) Percentage of CD44+CD24- or CD44+CD24-ESA+ cells in LncCCAT1-overexpressing adherent cells and LncCCAT1-KO spheroid cells of MCF-7 cells or MDA-MB-231 cells, respectively, detected by flow cytometric analysis. (**B**) mRNA levels of pluripotent transcription factors in LncCCAT1-overexpressing MCF-7 and MDA-MB-231 cells and LncCCAT1-knockdown MCF-7 and MDA-MB-231 spheroid cells. (**C, D**) The migration abilities of LncCCAT1-overexpressing adherent cells and LncCCAT1-KO spheroid cells of MCF-7 cells (C) or

MDA-MB-231 cells (D) detected by wound healing assay. Scale bar, 100μm. **P<0.01, and ***P<0.001 by two-tailed Student's *t*-test.

Supplementary Figure S3. Verification of MS2-LncCCAT1 overexpression, overexpression and KD efficiencies of miR-204/211, miR-148a/152, respectively.

(**A**) Expression (a) and distribution (b) of LncCCAT1 in MCF-7 cells transfected with either pcDNA3.1-MS2 or pcDNA3.1-MS2-CCAT1. **(B)** Enrichment of predicted miRNAs in RNAs retrieved from MS2-LncCCAT1 compared with control in MCF-7 cells detected by MS2-based RIP assay with anti-GFP antibody. (**C, D**) miR-204/211(C) or miR-148a/152(D) overexpression or knockdown efficiencies by transfecting miRNA mimics (left) and miRNAs inhibitors (right) in MCF-7 and MDA-MB-231 cells detected by qRT-PCR. All data are shown as the mean±SD. *P<0.05, **P<0.01, and ***P<0.001 by two-tailed Student's *t*-test.

Supplementary Figure S4. Expression levels of miR-204 and miR-211 in breast cancer tissues and cells.

(**A, B**) miR-204 (A) and miR-211 (B) levels were detected in 80 pairs of breast cancer tissues by qRT-PCR. The levels of miR-204/211 in breast cancer tissues were significantly higher than in adjacent normal tissues. (**C**) miR-204 and miR-211 levels were detected in adherent cells, spheroid cells, non-BCSCs and BCSCs of MCF-7 and MDA-MB-231 cells by qRT-PCR analysis. Relative gene expression values were normalized to those of endogenous U6. (**D**) miR-204 and miR-211 levels in LncCCAT1-overexpressing or knockdown or LncCCAT1-MUT-overexpressing MCF-7 cells detected by qRT-PCR analysis. Relative gene expression values were normalized to endogenous U6. (**E**) TCF4 mRNA levels were not altered after transfection with miR-204/211 mimic or miR-204/211 inhibitor in MCF-7 or MDA-MB-231 cells. All data are shown as the mean±SD. *P<0.05, **P<0.01, and ***P<0.001 by two-tailed Student's *t*-test.

Supplementary Figure S5. TCF4 promotes breast cancer cell proliferation, self-renewal, migration, and invasion *in vitro*. (**A, B**) TCF4 overexpression (A) and knockdown (B) efficiencies by transfecting TCF4 plasmid or si-TCF4 in MCF-7 or MDA-MB-231 cells detected by qRT-PCR (left) and western blotting (right). (**C**) Proliferative abilities of TCF4-overexpressing MCF-7 cells and LncCCAT1-knockdown MDA-MB-231 cells detected by CCK-8 assays. (**D**) Sphere-formation abilities of TCF4-overexpressing MCF-7 cells and TCF4-knockdown MDA-MB-231 cells. The sphere-formation efficiencies (SFEs) are shown on the right. Scale bar, 100 μm. (**E**) Migration (upper) and invasion (lower) abilities of TCF4-overexpressing MCF-7 cells and

LncCCAT1-knockdown MDA-MB-231 cells detected by transwell migration and invasion assays. Scale bar, 100 µm. All data are shown as the mean±SD. **P*<0.05, ***P*<0.01, and ****P*<0.001 by two-tailed Student's *t*-test.

Supplementary Figure S6. Expression levels of miR-148a and miR-152 in breast cancer tissues and cells. (**A, B**) miR-148a (A) and miR-152 (B) levels were detected in 80 pairs of breast cancer tissues by qRT-PCR. The levels of miR-148a/152 in breast cancer tissues were significantly higher than those in adjacent normal tissues. (**C**) miR-148a and miR-152 levels were detected in adherent cells, spheroid cells, non-BCSCs and BCSCs of MCF-7 and MDA-MB-231 cells by qRT-PCR analysis. Relative gene expression values were normalized to those of endogenous U6. (**D**) miR-148a and miR-152 levels in LncCCAT1-overexpressing or knockdown or LncCCAT1-MUT-overexpressing MCF-7 cells detected by qRT-PCR analysis. Relative gene expression values were normalized to endogenous U6. All data are shown as the mean±SD. *P<0.05, **P<0.01, and ***P<0.001 by two-tailed Student's *t*-test.

Supplementary Figure S7. LncCCAT1 inhibits FAT4 expression through upregulation of DNMT1. (**A, B**) mRNA levels of potential genes downregulated by both LncCCAT1 and DNMT1 in MCF-7 (A) and MDA-MB-231 (B) cells transfected with LncCCAT1, LncCCAT1 plus si-DNMT1, and LncCCAT1 together with 5 µM 5-Aza. (**C, D**) FAT4 knockdown efficiency following transfection with FAT4 siRNAs in MCF-7 (C) and MDA-MB-231 cells (D) detected by qRT-PCR (left) and western blotting (right). All data are shown as the mean±SD. *P<0.05, **P<0.01, and ***P<0.001 by two-tailed Student's *t*-test.

Supplementary Figure S8. ANXA2 promotes breast cancer cell proliferation, self-renewal, migration, and invasion *in vitro*. (**A**) MS/MS profiles of the ANXA2 band retrieved by LncCCAT1. The corresponding peptide sequences are listed on the top of the graphs. (**B**) ANXA2 protein levels were not altered after transfection with oeLncCCAT1 or si-LncCCAT1 in MCF-7 (left) or MDA-MB-231 (right) cells as detected by western blotting. (**C**) ANXA2 overexpression and knockdown efficiencies by transfecting ANXA2 lentivirus or sh-ANXA2 lentivirus in MCF-7 (left) or MDA-MB-231 (right) cells detected by qRT-PCR (upper) and western blotting (lower). (**D**) Proliferative abilities of ANXA2-overexpressing MCF-7 cells and ANXA2-knockdown MDA-MB-231 cells detected by CCK-8 assays. (**E**) Sphere-formation abilities of ANXA2-overexpressing MCF-7 cells and ANXA2-knockdown MDA-MB-231 cells. The sphere-formation efficiencies (SFEs) are shown on the

right. Scale bar, 100 μm. (**F**) Migration (upper) and invasion (lower) abilities of ANXA2-overexpressing MCF-7 cells and ANXA2-knockdown MDA-MB-231 cells detected by transwell assays. Scale bar, 100 μm. (**G**) Protein levels of crucial genes involved in the Wnt/β-catenin signaling pathway and EMT in MCF-7 (left) and MDA-MB-231 (right) cells transfected with oeANXA2 or si-ANXA2 detected by western blotting. All data are shown as the mean±SD. *P<0.05, **P<0.01, and ***P<0.001 by two-tailed Student's t-test.

Supplementary Figure S9. LncCCAT1 promotes breast cancer cells stemness *in vivo* by binding miR-204/211, miR-148a/152 and ANXA2. (A-C) Images (A) of tumors and index (B,C) of tumors harvested after serially diluted different MCF-7 cells were planted. **(D)** Representative immunohistochemical staining of Nanog, Sox2, Oct4, ALDH1A1, CD44 and CD24 of the tumors from subcutaneously implanted mice. Scale bar, 100 μm.

Supplementary Figure S10. Representative immunohistochemical staining of Wnt target genes including c-Myc, c-Jun, LEF1, MMP-7, Cyclin D1，TCF1/TCF7 and Axin2 of the tumors from subcutaneously implanted mice. Scale bar, 100 μm.

Supplementary Table S1. LncCCAT1 expression and clinicopathological features in 80 patients with breast cancer.

Supplementary Table S2. Sequences of primers used for qRT-PCR, plasmid construction and ChIP-qPCR.

Supplementary Table S3. Antibodies used for western blotting (WB), immunoprecipitation (IP), immunofluorescence (IF) and flow cytometry (FC).

Supplementary Table S4. Sequences of siRNAs and sgRNAs against specific targets.

Supplementary Figure S1

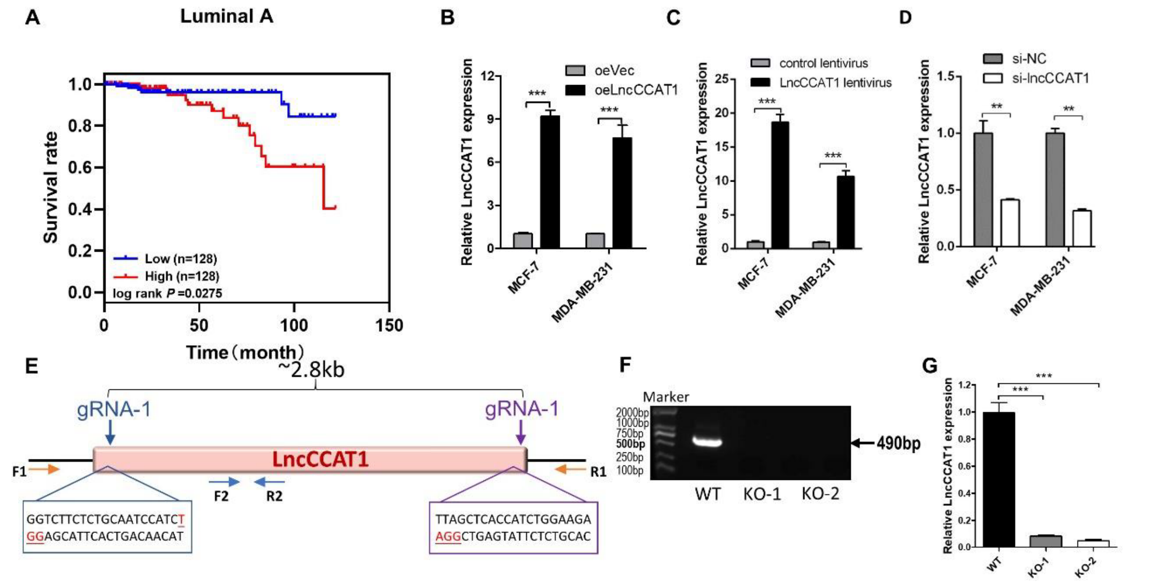

H

WT

...TGCTGTTCTTTGAAGGCATTCAAGCCTTAATAGCTAGCTGGATGAATGTTTAACTTCTAGGCCAGGCACTACTCTGTCCCAACAATAAGCCCTGTACATTGGGAA
AGGTGCCGAGACATGAACTTTGGTCTTCTCTGCAATCCATC**TGG**.......2203bp..............TTAGCTCACCATCTGGAAGA**AGG**CTGAGTATTCTCTGCACTCACATAG
TAGCTAATGCCTACTCCCCAGCCACCCACAATTCTTTCTGTAGGAAGGCTCGCTAGAATACTTTGTGATATTGGATATTAGTTCCATA...

KO-1

...TGCTGTTCTTTGAAGGCATTCAAGCCTTAATAGCTAGCTGGATGAATGTTTAACTTCTAGGCCAGGCACTACTCTGTCCCAACAATAAGCCCTGTACATTGGGAA
AGGTGCCGAGACATGAACTTTGGTCTTCTCTGCA...................................//...AGA**AGG**CTGAGTATTCTCTGCACTCACA
TAGTAGCTAATGCCTACTCCCCAGCCACCCACAATTCTTTCTGTAGGAAGGCTCGCTAGAATACTTTGTGATATTGGATATTAGTTCCATA...

KO-2

...TGCTGTTCTTTGAAGGCATTCAAGCCTTAATAGCTAGCTGGATGAATGTTTAACTTCTAGGCCAGGCACTACTCTGTCCCAACAATAAGCCCTGTACATTGGGAA
AGGTGCCGAGACATGAACTTTGGTC..//..**AGG**CTGAGTATTCTCTGCACTC
ACATAGTAGCTAATGCCTACTCCCCAGCCACCCACAATTCTTTCTGTAGGAAGGCTCGCTAGAATACTTTGTGATATTGGATATTAGTTCCATA...

Supplementary Figure S2

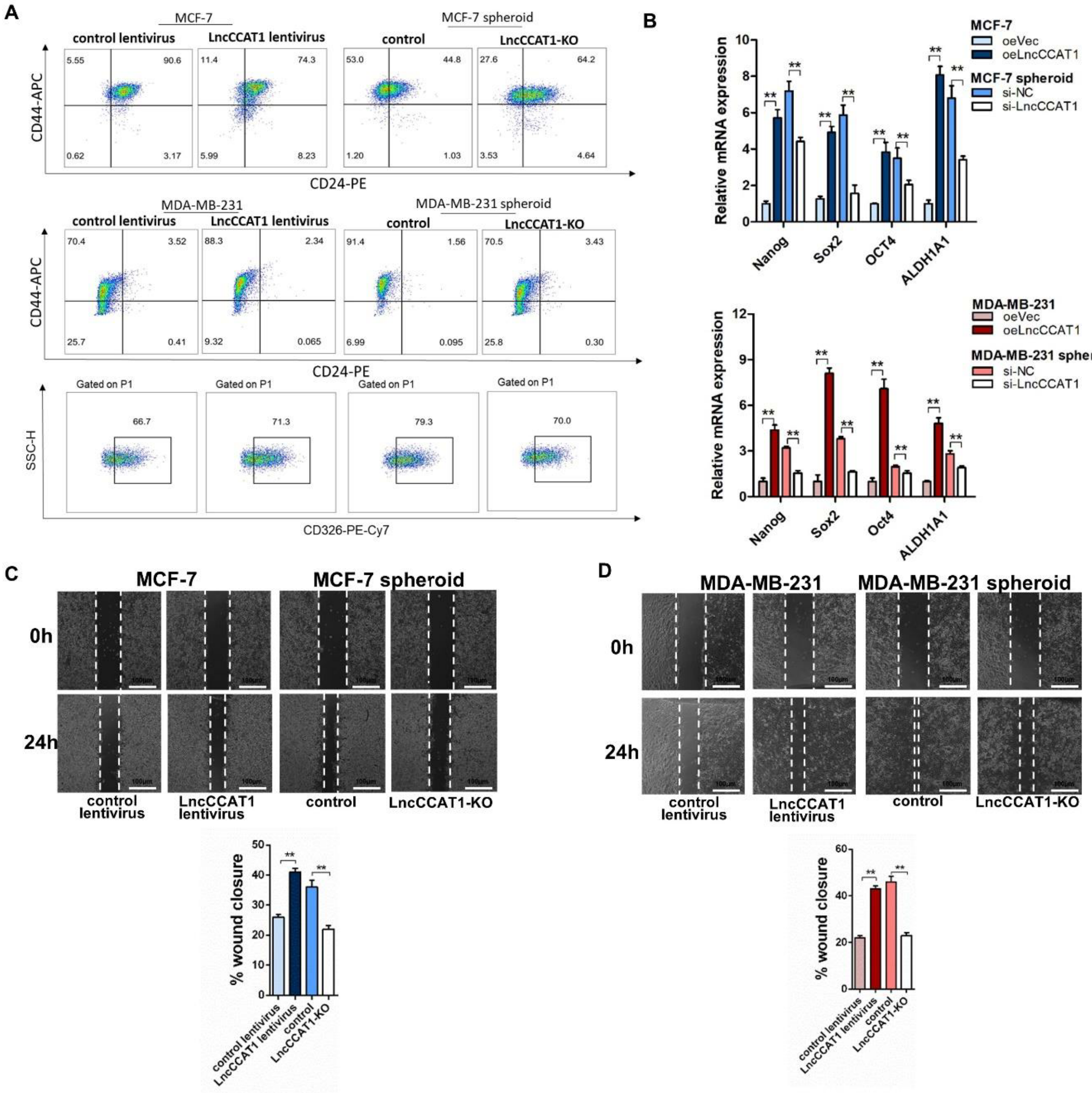

Supplementary Figure S3

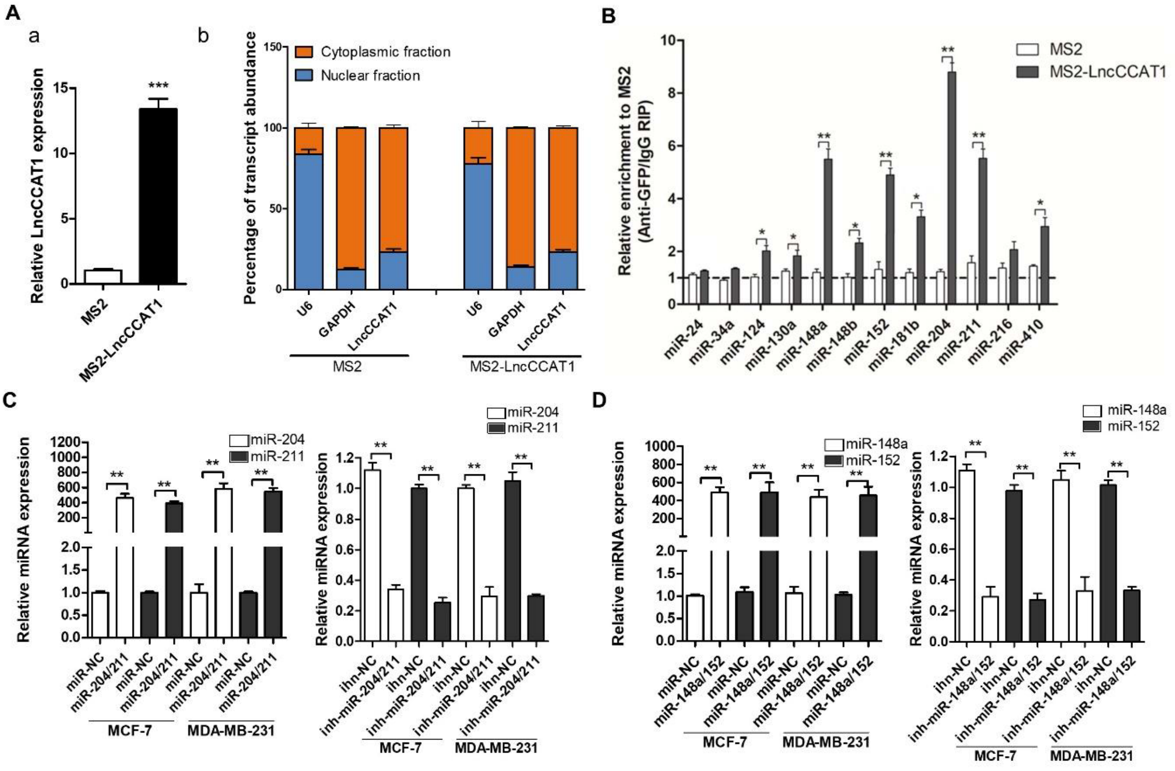

Supplementary Figure S4

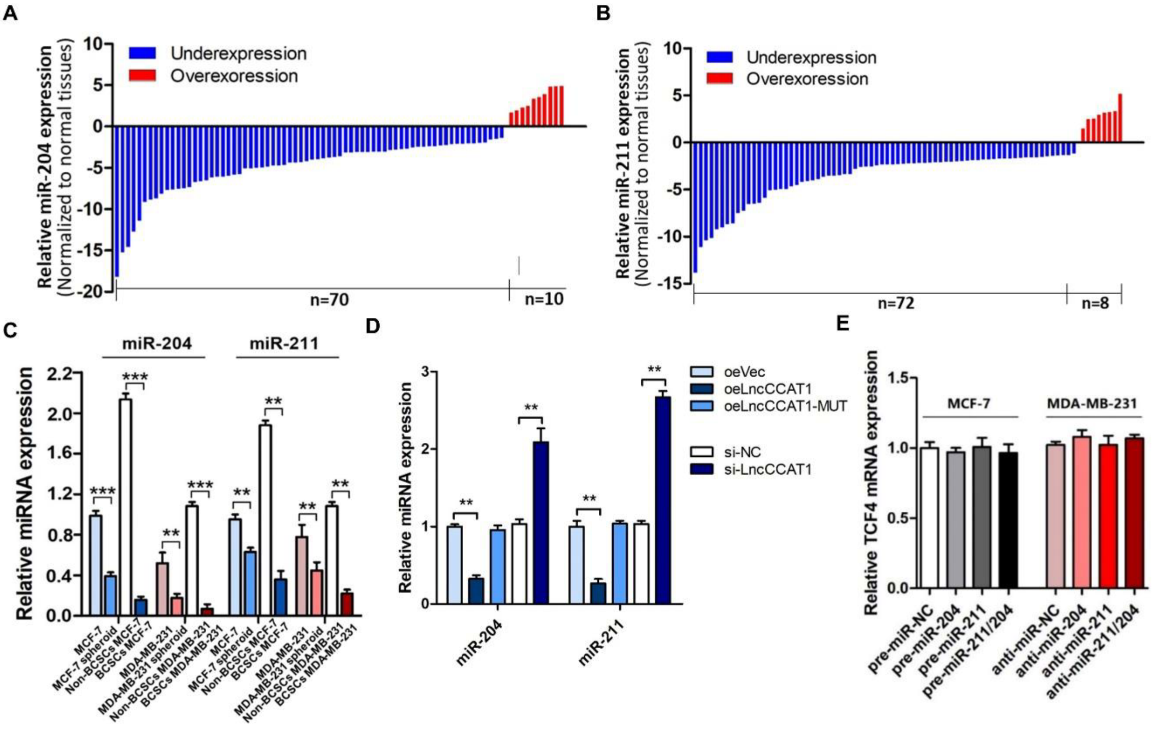

Supplementary Figure S5

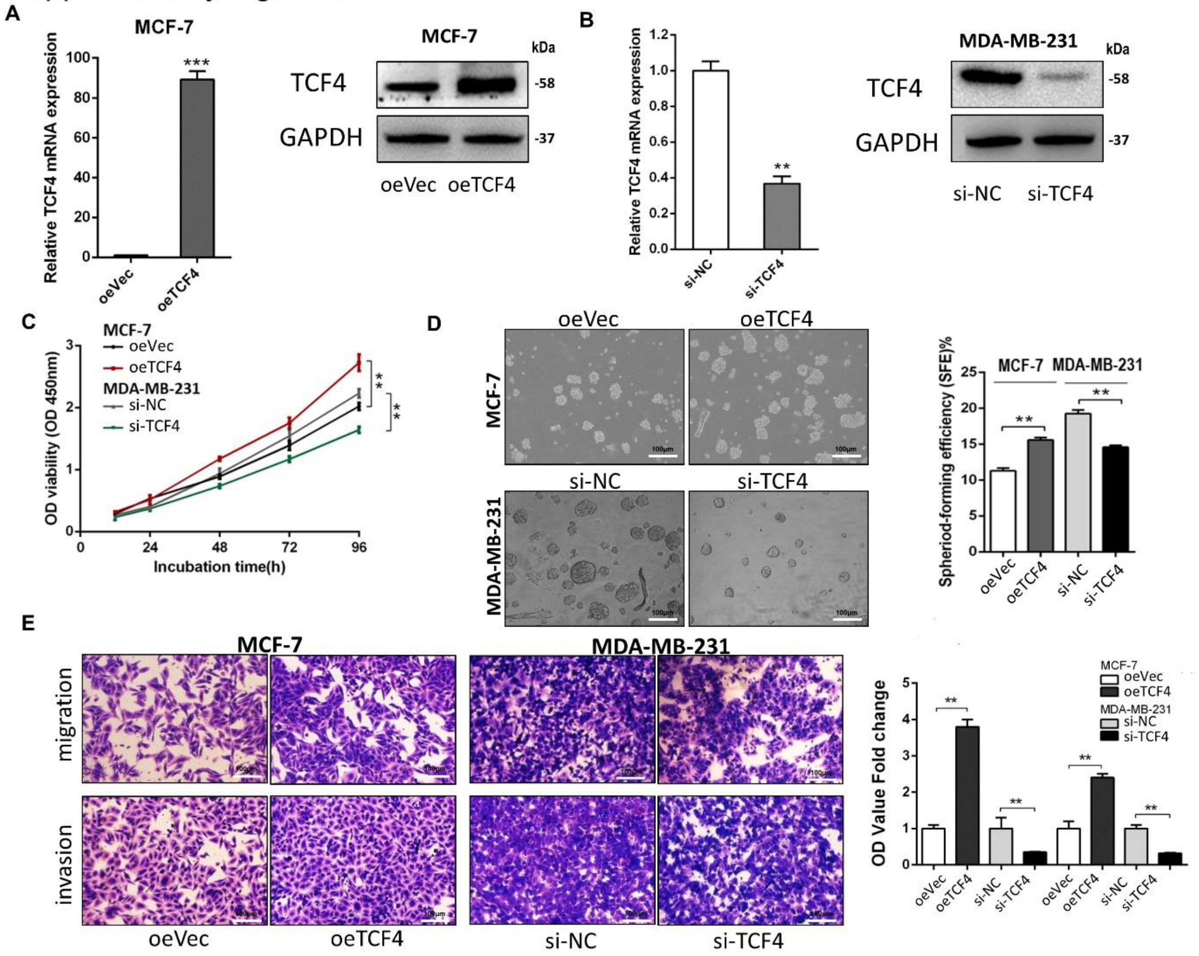

Supplementary Figure S6

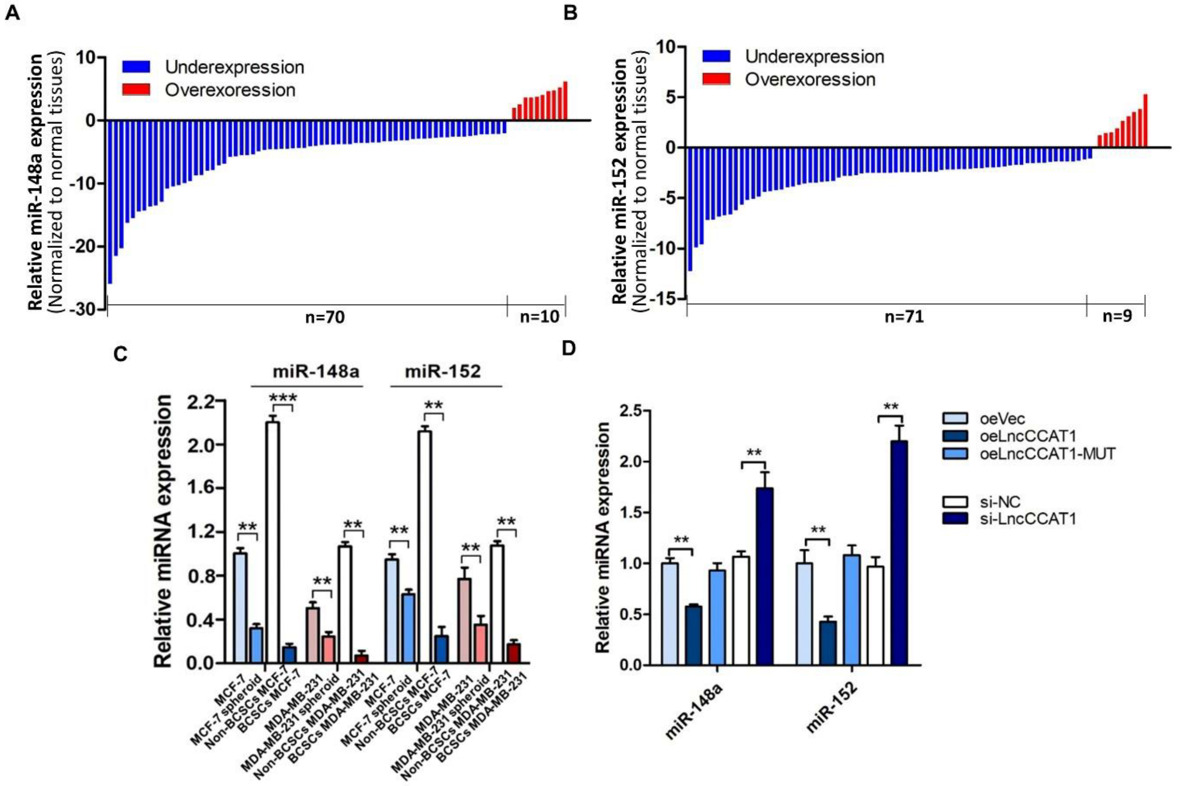

Supplementary Figure S7

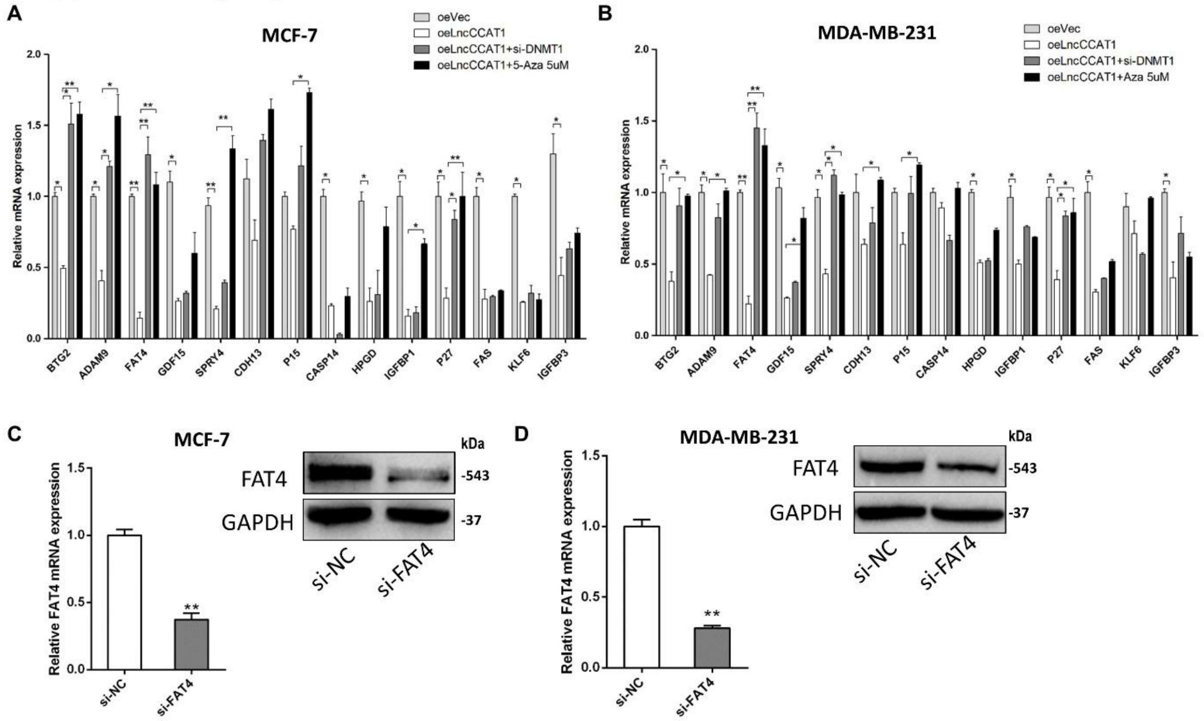

Supplementary Figure S8

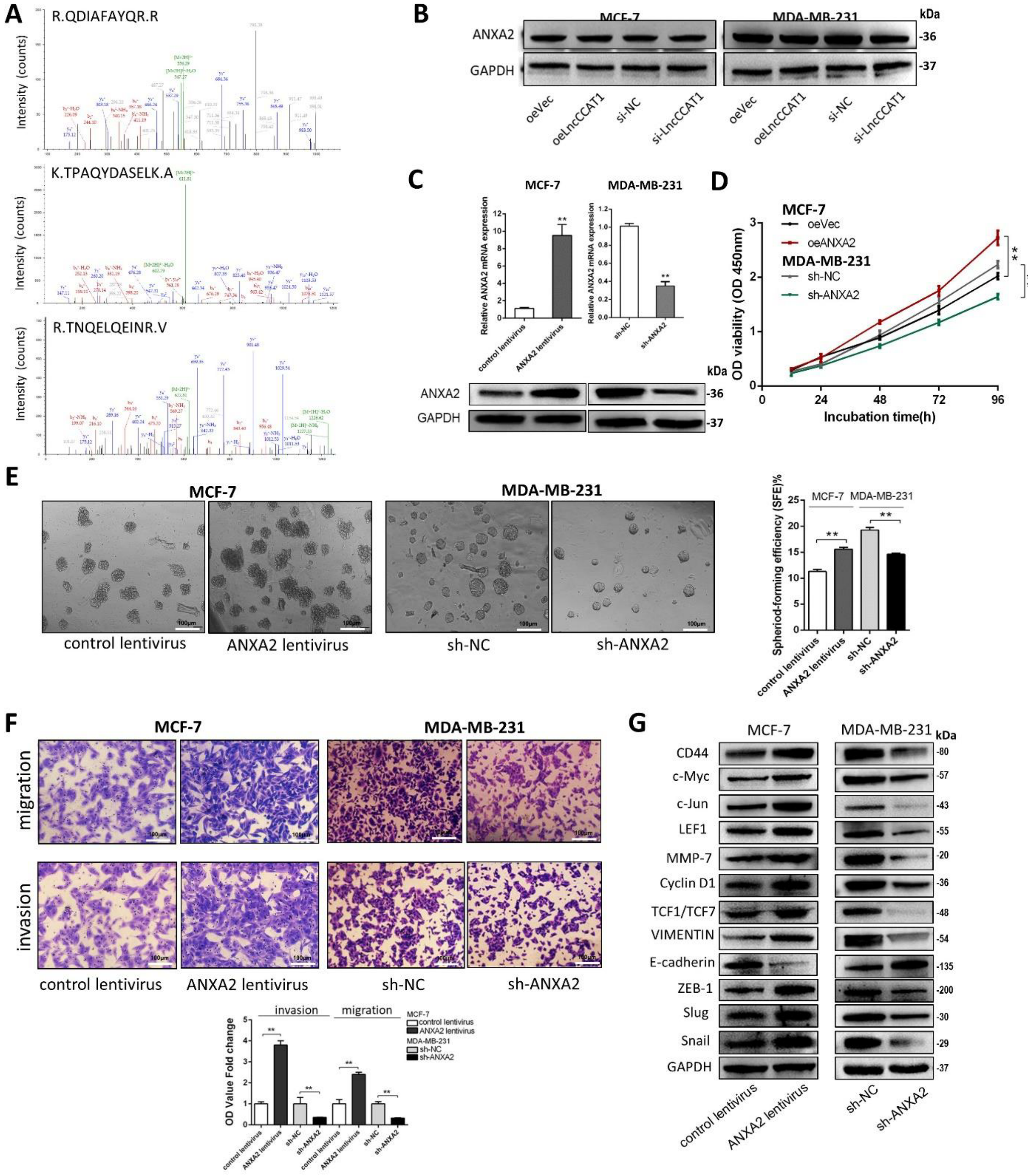

Supplementary Figure S9

A

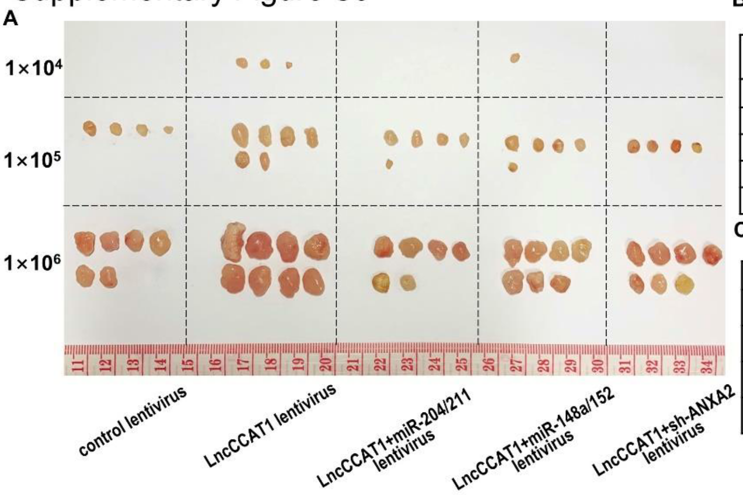

B

Confidence intervals for 1/(stem cell frequency)

Group	Lower	Estimate	Upper
control lentivirus	1003625	467739	217990
LncCCAT1 lentivirus	114127	52780	24409
LncCCAT1+miR-204/211 lentivirus	885431	409580	189462
LncCCAT1+miR-148a/152 lentivirus	580359	247231	105320
LncCCAT1+sh-ANXA2 lentivirus	731120	329276	148297

C

Pairwise tests for differences in stem cell frequencies

Group 1	Group 2	Chisq	DF	Pr(>Chisq)
control lentivirus	LncCCAT1 lentivirus	16.6	1	4.54e-05
LncCCAT1+miR-204/211 lentivirus	LncCCAT1 lentivirus	15.1	1	0.000104
LncCCAT1+miR-148a/152 lentivirus	LncCCAT1 lentivirus	8.6	1	0.00337
LncCCAT1+sh-ANXA2 lentivirus	LncCCAT1 lentivirus	11.4	1	0.000716

D

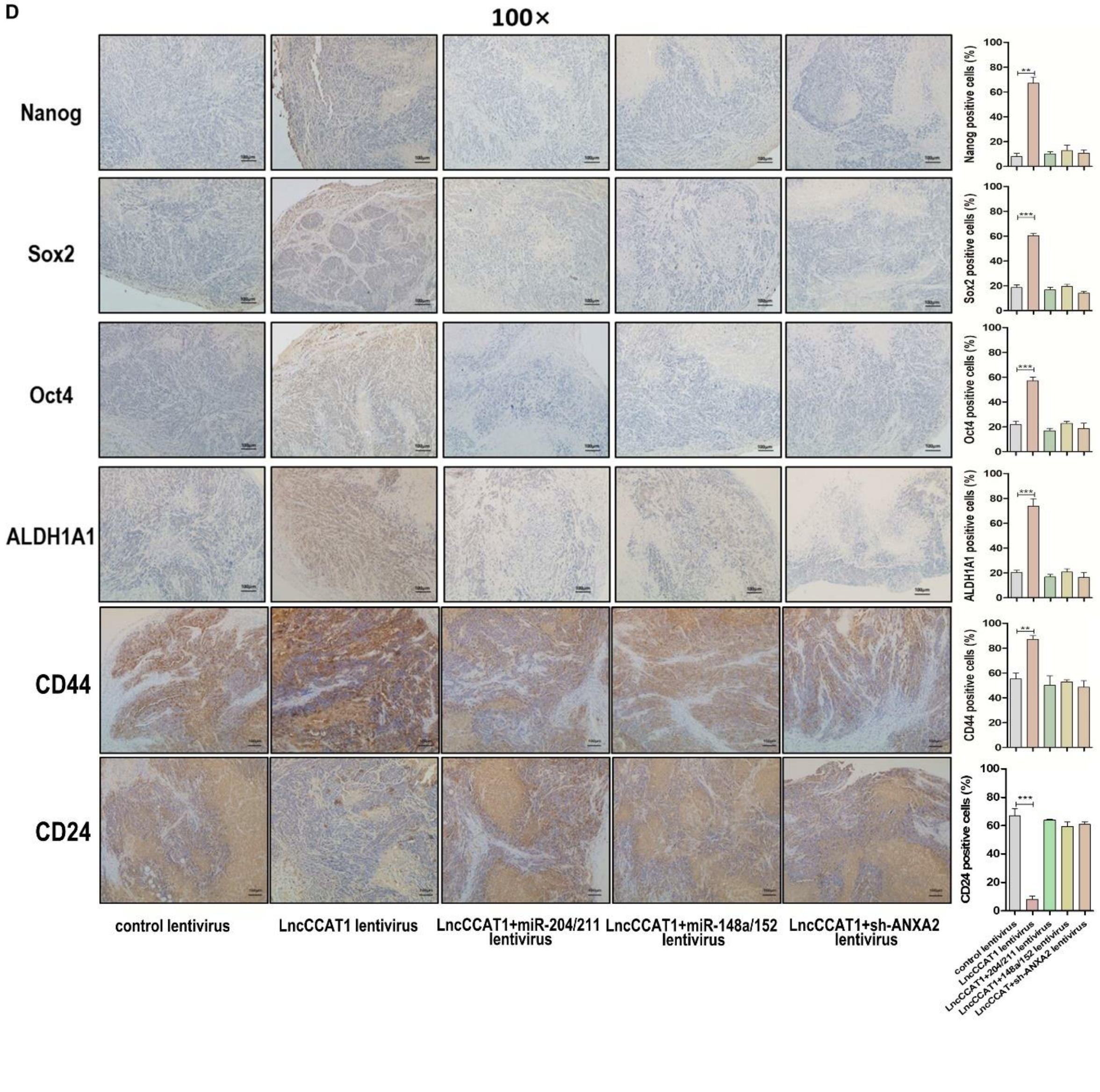

Supplementary Figure S10

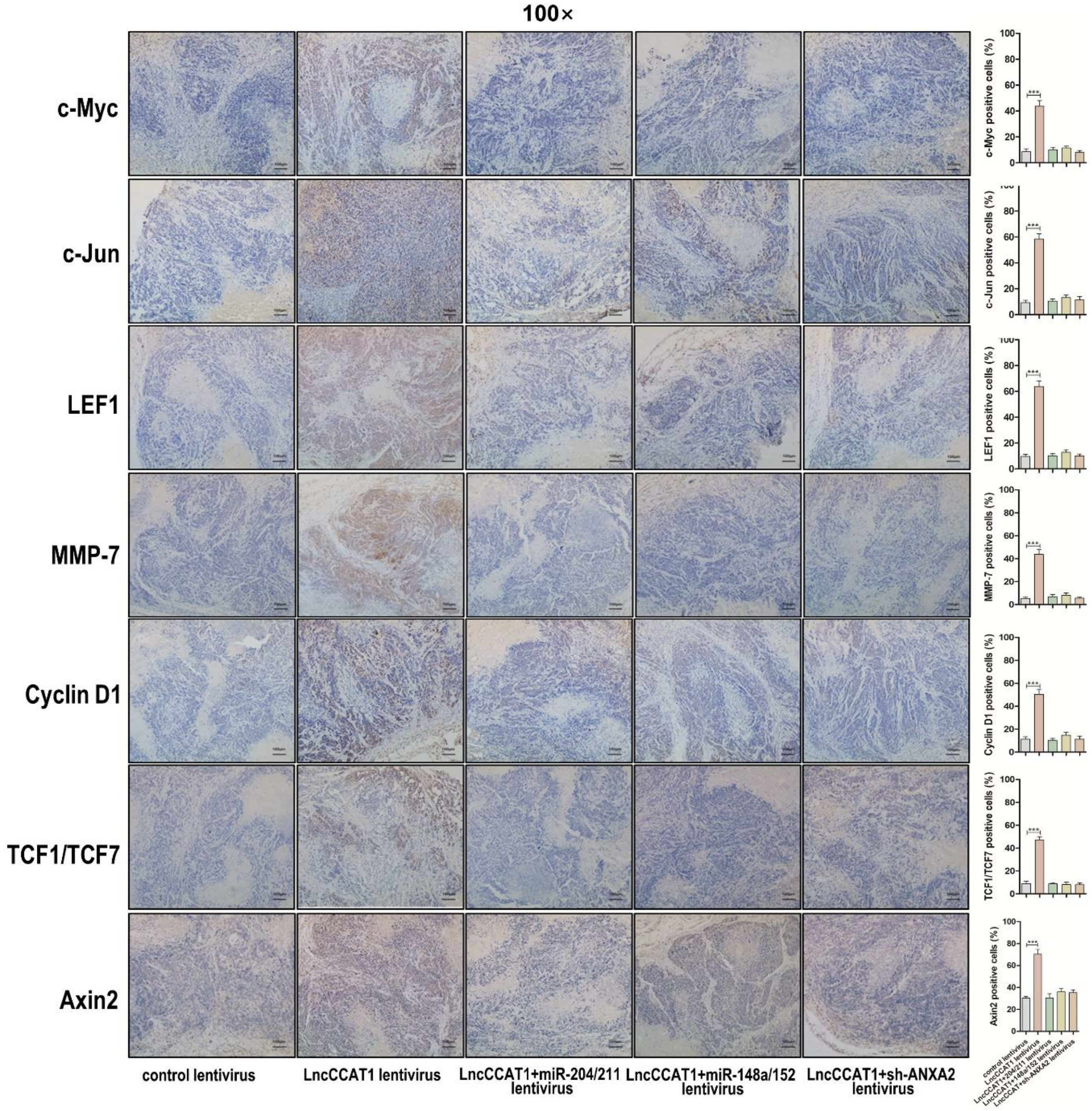

Supplementary Table S1. LncCCAT1 expression and clinicopathological features in 80 patients with breast cancer

Characteristics	Expression of LncCCAT1		p value*
	low	high	
**Sex**			
male	0	0	
female	40	40	
**Age**			0.239
≤60	31	35	
>60	9	5	
**Grade**			0.024*
Ⅰ/I-II, well-differentiated	4	0	
Ⅱ/II-III, moderately differentiated	20	14	
Ⅲ, poorly differentiated	16	26	
**Tumor histological**			0.006*
Ductal carcinoma in situ	11	2	
Invasive ductal carcinoma	29	38	
**T Classification**			0.058
T1	12	5	
T2	27	30	
T3	1	5	
**N Classification**			0.057
N0	17	6	
N1	13	21	
N2	6	8	
N3	4	5	
**ER status**			0.166
Negative	12	18	
Positive	28	22	
**PR status**			0.117
Negative	16	23	
Positive	24	17	
**HER2 status**			0.066
Negative	6	13	
Positive	34	27	
**Tumor size(cm^3)**			0.004*
≤ 6	27	14	
> 6	13	26	
**Lymph node metastasis**			0.000*
Negative	29	4	
Positive	11	36	

Median expression level was used as a cutoff to divide the 80 patients into LncCCAT1 low group (n = 40) and LncCCAT1 high group (n = 40).

Two-sided $\chi 2$ test. * $p<0.05$

Supplementary Table S2. Sequences of primers used for qRT-PCR, plasmid construction and ChIP-qPCR

Primer names	Sequences
**Sequences of primers used for qRT-PCR**	
LncCCAT1 forward	5'-TCACTGACAACATCGACTTTGAAG-3'
LncCCAT1 reverse	5'-GGAGAAAACGCTTAGCCATACAG-3'
hsa-miR-148a forward	5'-AATCGAACCTTCAGTGCACTACA-3'
hsa-miR-148a reverse	5'-TATGCTTGTTCTCGTCTCTGTGTC-3'
hsa-miR-152 forward	5'-GTAACTCGCTCAGTGCATGACA-3'
hsa-miR-152 reverse	5'-TATGGTTGTAGACGACTCCTTGAC-3'
hsa-miR-204 forward	5'-CGATTCCATTTGTGCTTGATCT-3'
hsa-miR-204 reverse	5'-TATGGTTTTGACGACTGTGTGAT-3'
hsa-miR-211 forward	5'-CGCTTCCCTTTGTCATCCT-3'
hsa-miR-211 reverse	5'-TATGGTTTTGACGACTGTGTGAT-3'
Nanog forward	5'-TCCTCCTCTTCCTCTATACTAAC-3'
Nanog reverse	5'-CCCACAATCACAGGCATAG-3'
Sox-2 forward	5'-AACCCCAAGATGCACAACTC-3'
Sox-2 reverse	5'-GCTTAGCCTCGTCGATGAAC-3'
Oct-4 forward	5'-GGACCAGTGTCCTTTCCTCT-3'
Oct-4 reverse	5'-CCAGGTTTTCTTTCCCTAGC-3'
ALDH1A1 forward	5'-GCACGCCAGACTTACCTGTC-3'
ALDH1A1 reverse	5'-CCTCCTCAGTTGCAGGATTAAAG-3'
BTG2 forward	5'-TGGGCTTAGGGAACCATCTCT-3'
BTG2 reverse	5'-TTCAGCCAAGGAATACATGCAA-3'
ADAM9 forward	5'-AAGTACCATGACAACGCCCAAT-3'
ADAM9 reverse	5'-CATTCTCGGAGTGGTCCATGT-3'
FAT4 forward	5'-AGACGGGACTTATCACGGTG-3'
FAT4 reverse	5'-GGAACTTCACTACCGGGTCATT-3'
GDF15 forward	5'-CTCCAGATTCCGAGAGTTGC-3'
GDF15 reverse	5'-AGAGATACGCAGGTGCAGGT-3'
GAPDH forward	5'-GAGTCAACGGATTTGGTCGT-3'
GAPDH reverse	5'-TTGATTTTGGAGGGATCTCG-3'
SPRY4 forward	5'-CCAGGATGTCACCCACCATTG-3'
SPRY4 reverse	5'-TGTGCTGCTGCTGCTC-3'
CDH13 forward	5'-AGTGTTCCATATCAATCAGCCAG-3'
CDH13 reverse	5'-CGAGACCTCATAGCGTAGCTT-3'
P15 forward	5'-TCTCCGTTGGCCGGAGGTCA-3'
P15 reverse	5'-TGCGCAGGTACCCTGCAACG-3'
CASP14 forward	5'-TGCACGTTTATTCCACGGTA-3'
CASP14 reverse	5'-TTCAGGGTTCGTTTTCCTTG-3'
HPGD forward	5'-CTGCACCATGCACGTGAACG-3'
HPGD reverse	5'-AAGTGTCTCTCAGTTGTTGCTG-3'
IGFBP1 forward	5'-TTGGGACGCCATCAGTACCTA-3'
IGFBP1 reverse	5'-TTGGCTAAACTCTCTACGACTCT-3'

P27 forward	5'-TAATTGGGGCTCCGGCTAACT-3'
P27 reverse	5'-TGCAGGTCGCTTCCTTATTCC-3'
FAS forward	5'-CTTTTCGTGAGCTCGTCTCTGA-3'
FAS reverse	5'-CTCCCCAGAAGCGTCTTTGA-3'
KLF6 forward	5'-CTGCCGTCTCTGGAGGAGT-3'
KLF6 reverse	5'-TCCACAGATCTTCCTGGCTGTC-3'
IGFBP3 forward	5'-AGACACACTGAATCACCTGAAGT-3'
IGFBP3 reverse	5'-AGGGCGACACTGCTTTTTCTT-3'
ANXA2 forward	5'-TCTACTGTTCACGAAATCCTGTG-3'
ANXA2 reverse	5'-AGTATAGGCTTTGACAGACCCAT-3'
TCF4 forward	5'-CAAGCACTGCCGACTACAATA-3'
TCF4 reverse	5'-CCAGGCTGATTCATCCCACTG-3'
**Sequences of primers used for plasmid construction**	
pcDNA3.1-CCAT1 forward	5'-CGCGGATCCGCCACCTTTAAATCATACCAATTGAA-3'
pcDNA3.1-CCAT1 reverse	5'-CCCAAGCTTCCAAGACTTAATATACTTATATTTA-3'
pcDNA3.1-ANXA2 forward	5'-CGCGGATCCATGGGCCGCCAGCTAGCGGG-3'
pcDNA3.1-ANXA2 reverse	5'-CCGCTCGAGTCAGTCATCTCCACCACACAG-3'
pcDNA3.1-TCF4 forward	5'-CGCGGATCCATGCATCACCAACAGCGAAT-3'
pcDNA3.1-TCF4 reverse	5'-CCGCTCGAGTTACATCTGTCCCATGTGATTCGAT-3'
S1(1-785) forward	5'-TAATACGACTCACTATAGGGATAAGGCAAGTTTTCCTGTGTGGCT-3'
S1(1-785) reverse	5'-TTTAAATCATACCAATTGAACCGAG-3'
S2(630-1225) forward	5'-TAATACGACTCACTATAGGGGAGAAGGGGTGAAGGGACACTGGCA-3'
S2(630-1225) reverse	5'-ACTGCCCTGAAACTTATATGCTGTT-3'
S3(1226-1883) forward	5'-TAATACGACTCACTATAGGGATGACAGGGTTTGTGCTGCTATGAG-3'
S3(1226-1883) reverse	5'-GAGGCAGGAGGGTGCTTGACAATAA-3'
S4(1884-2504) forward	5'-TAATACGACTCACTATAGGGCCATCCACACCTTGCCTGAAATACT-3'
S4(1884-2504) reverse	5'-GAACTGCACATGCGAGGGATCTAGG-3'
S5(1884-2795) forward	5'-TAATACGACTCACTATAGGGCCAAGACTTAATATACTTAT-3'
S5(1884-2795) reverse	5'-CCTGTCATGAACTGCACATGCGAGG-3'
pmirGLO-CCAT1-WT (miR148a/152) forward	5'-TCGAGGCATTCACTGACAACATCGACTTTGAAGTTGCACTGACCTGGCCAGCCCTGCCT-3'
pmirGLO-CCAT1-WT (miR148a/152) reverse	5'-CTAGAGGCAGGGCTGGCCAGGTCAGTGCAACTTCAAAGTCGATGTTGTCAGTGAATGCC-3'
pmirGLO-CCAT1-MUT (miR148a/152) forward	5'-TCGAGGCATTCACTGACGCAGCATGACACTTGACGATGACTGACTGGCCAGCCCTGCCT-3'
pmirGLO-CCAT1-MUT (miR148a/152) reverse	5'-CTAGAGGCAGGGCTGGCCAGTCAGTCATCGTCAAGTGTCATGCTGCGTCAGTGAATGCC-3'
pmirGLO-CCAT1-WT (miR204/211) forward	5'-TCGAGAATGCCCTGTTAAGTAAACGAACACGAAATTCAAAG

	GGAAACAGGAGCAATCAT-3'
pmirGLO-CCAT1-WT (miR204/211) reverse	5'-CTAGATGATTGCTCCTGTTTCCCTTTGAATTTCGTGTTCGTTT ACTTAACAGGGCATTC-3'
pmirGLO-CCAT1-MUT (miR204/211) forward	5'-TCGAGAATGCCCTGTTAAGTAACAAGCTGTACCGGCTGCGTA TCGCCAGGAGCAATCAT-3'
pmirGLO-CCAT1-MUT (miR204/211) reverse	5'-CTAGATGATTGCTCCTGGCGATACGCAGCCGGTACAGCTTGT TACTTAACAGGGCATTC-3'
pmirGLO-TCF4-WT forward	5'-TCGAGTGTATCCCCATTAAAAAAGAAAAAAAGGGAATGTGG CCTTTTTAGTGTGTTTTT-3'
pmirGLO-TCF4-WT reverse	5'-CTAGAAAAACACACTAAAAAGGCCACATTCCCTTTTTTTCTT TTTTAATGGGGATACAC-3'
pmirGLO-TCF4-MUT forward	5'-TCGAGTGTATCCCAGCCGCTGCCTGCAGCGCTATGCTGTGGC CTTTTTAGTGTGTTTTT-3'
pmirGLO-TCF4-MUT reverse	5'-CTAGAAAAACACACTAAAAAGGCCACAGCATAGCGCTGCAG GCAGCGGCTGGGATACAC-3'
**Sequences of primers used for ChIP-qPCR**	
P1 forward	5'-CTCTTTACAAATGGGATTCTTC-3'
P1 reverse	5'-CACGGCAGCTGGTAATGAT-3'
P2 forward	5'-TGATTCACCAAATTAATCTTGGC-3'
P2 reverse	5'-AGCATCTGGGACAAACTCTCC-3
P3 forward	5'-CCATGTAGTCCTGTCTGTTCTACTG-3'
P3 reverse	5'-AATCCTGTTCTTGTCTATAATGGCA-3'
Neg forward	5'-GTGTTTGCACATGGAAAATTCC-3'
Neg reverse	5'-TGGACCTTAAAGTTTCTCTA-3'

Supplementary Table S3. Antibodies used for western blotting (WB), immunoprecipitation (IP), immunofluorescence (IF) and flow cytometry (FC).

Protein	Applications	Antibody	Origin	dilution	Molecular weight
GAPDH	WB	D16H11, Cell Signaling Technology	Rabbit	1:1000	37 KD
Sox2	WB	sc-365964, Santa cruz	Mouse	1:500	34KD
4-Oct	WB	sc-101534, Santa cruz	Mouse	1:500	52KD
Nanog	WB	sc-293121, Santa cruz	Mouse	1:500	40KD
ALDH1A1	WB	sc-374076, Santa cruz	Mouse	1:500	56KD
DNMT1	WB	Ab19905, Abcam	Rabbit	1:1000	183KD
TCF4	WB	2565, Cell Signaling Technology	Rabbit	1:1000	58KD
FAT4	WB	ab130076, Abcam	Rabbit	1:1000	543KD
CD44	WB	3570, Cell Signaling Technology	Mouse	1:1000	80KD
c-Myc	WB	5605, Cell Signaling Technology	Rabbit	1:1000	57-65KD
c-Jun	WB	9165, Cell Signaling Technology	Rabbit	1:1000	43KD
LEF1	WB	2230, Cell Signaling Technology	Rabbit	1:1000	55KD
MMP-7	WB	3801, Cell Signaling Technology	Rabbit	1:1000	20-22KD
Cyclin D1	WB	2978, Cell Signaling Technology	Rabbit	1:1000	36KD
TCF1/TCF7	WB	2203, Cell Signaling Technology	Rabbit	1:1000	48,50KD
β-catenin	WB, IF, IP	8480, Cell Signaling Technology	Rabbit	1:1000, 1:100, 1:25	92KD
Histone H3	WB	4499, Cell Signaling Technology	Rabbit	1:1000	17KD
E-cadherin	WB	3195, Cell Signaling Technology	Rabbit	1:1000	135KD
Vimentin	WB	ab92547, Abcam	Rabbit	1:1000	54KD
ZEB-1	WB	3396, Cell Signaling Technology	Rabbit	1:1000	200KD
Slug	WB	9585, Cell Signaling Technology	Rabbit	1:1000	30KD
Snail	WB	3879, Cell Signaling Technology	Rabbit	1:1000	29KD
ANXA2	WB, IF, IP	ab41803, Abcam	Rabbit	1:1000, 1:100, 1:50	38KD
GSK-3β	WB, IF, IP	9832, Cell Signaling Technology	Mouse	1:1000, 1:250, 1:50	46KD
Ago2	RIP	03-110, Merck Millipore	Mouse	1:10	100KD
GFP	RIP	ab290, Abcam	Rabbit	1:20	28KD
IgG	CHIP, RIP	ab18413, Abcam	Mouse	1:10	150kD
CD44	FC	559942, BD Pharmingen™	Mouse	1:200	81KD
CD24	FC	555428, BD Pharmingen™	Mouse	1:200	9KD
ESA	FC	25-9326-42, eBioscience	Mouse	1:100	35KD

Supplementary Table S4. Sequences of siRNAs and sgRNAs against specific targets

**RNA names**	**Sequences**
FAT4-siRNA-1	CTACGAAGATTGGCTGTGA
FAT4-siRNA-2	GACGCCAAGTCATCTTAGA
FAT4-siRNA-3	GGTCTCGGTTTCAGGTCAA
DNMT1-siRNA-1	TCTTTGCATTATAGGCCTC
DNMT1-siRNA-2	CAATGAGACTGACATCAAA
DNMT1-siRNA-3	GAGGCCTATAATGCAAAGA
ANXA2-siRNA-1	CGGCTGTATGACTCCATGA
ANXA2-siRNA-2	GACCAACCGCAGCAATGCA
ANXA2-siRNA-3	GTCTGTCAAAGCCTATACT
TCF4-siRNA-1	GGTCTAGAAATGGAGGACA
TCF4-siRNA-2	TGGCTATGCAGGAATGTTG
TCF4-siRNA-3	CAGACAGTATAATGGCAAA
LncCCAT-siRNA-1	GTAGAAACACTATCACCTA
LncCCAT-siRNA-2	CTAGAGAATCACCCAATCT
LncCCAT-siRNA-3	GAGTGAGATCAGCGTTATT
LncCCAT-ASO-1	TCACCCAATCTACTCCCATT
LncCCAT-ASO-2	AAGGTGCCGAGACATGAACT
LncCCAT-ASO-3	GTCCCAACTAGCAAGTGCAA
sgRNA1	GGTCTTCTCTGCAATCCATCTGGAGCATTCACTGACAACAT
sgRNA2	TTAGCTCACCATCTGGAAGAAGGCTGAGTATTCTCTGCAC

Figure 2E

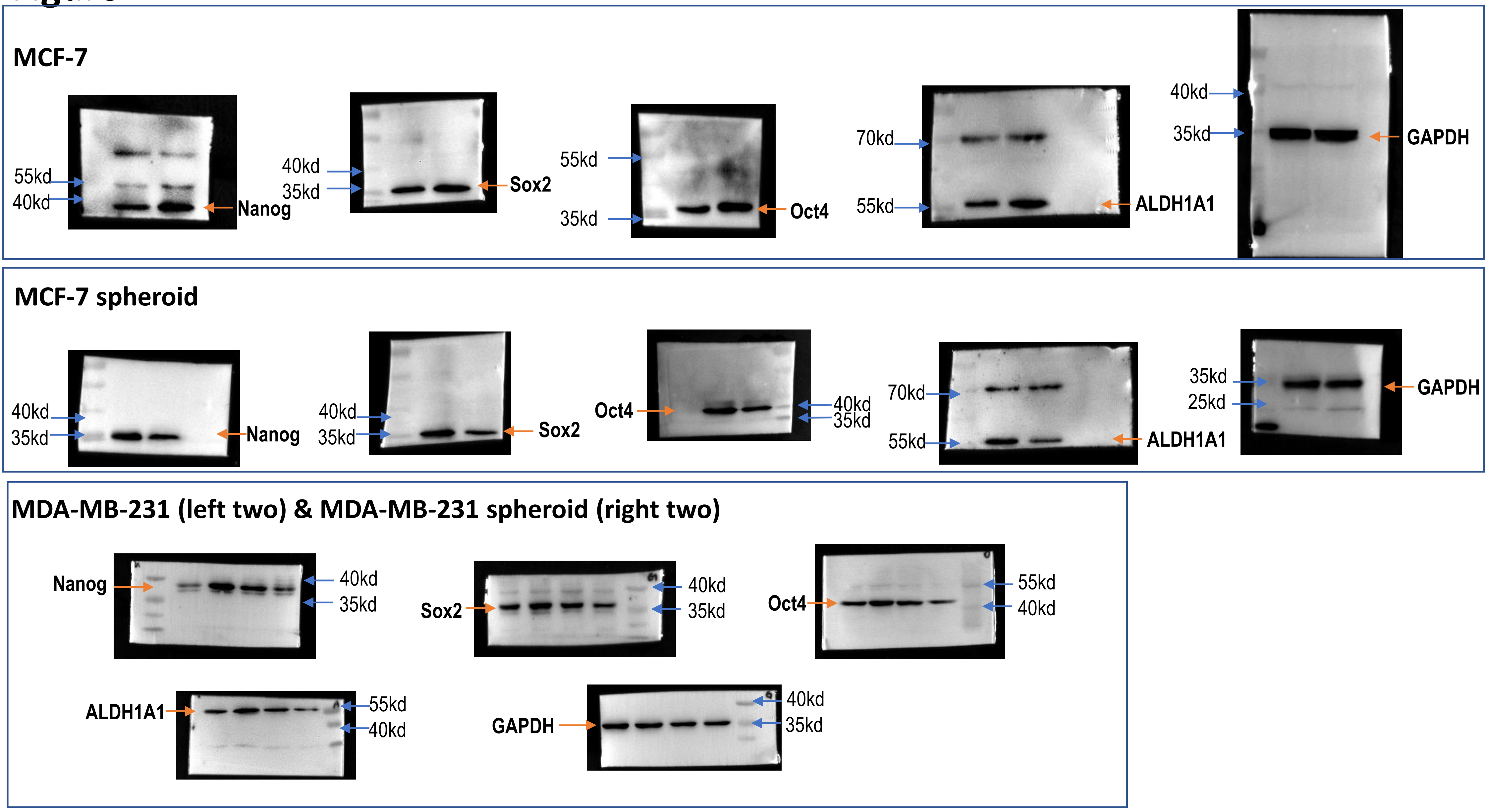

Figure 3I

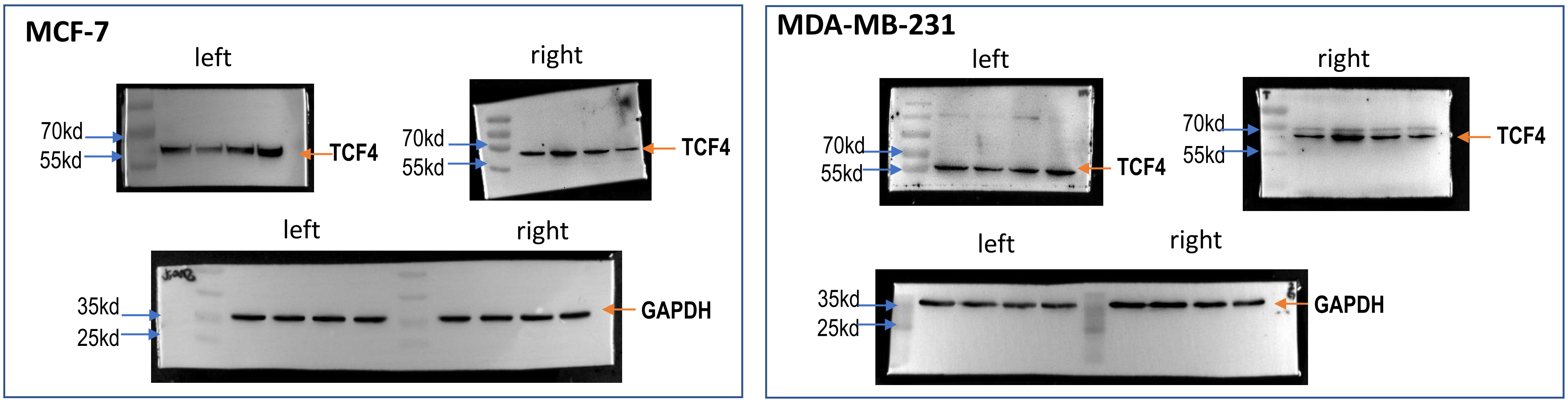

Figure 3J

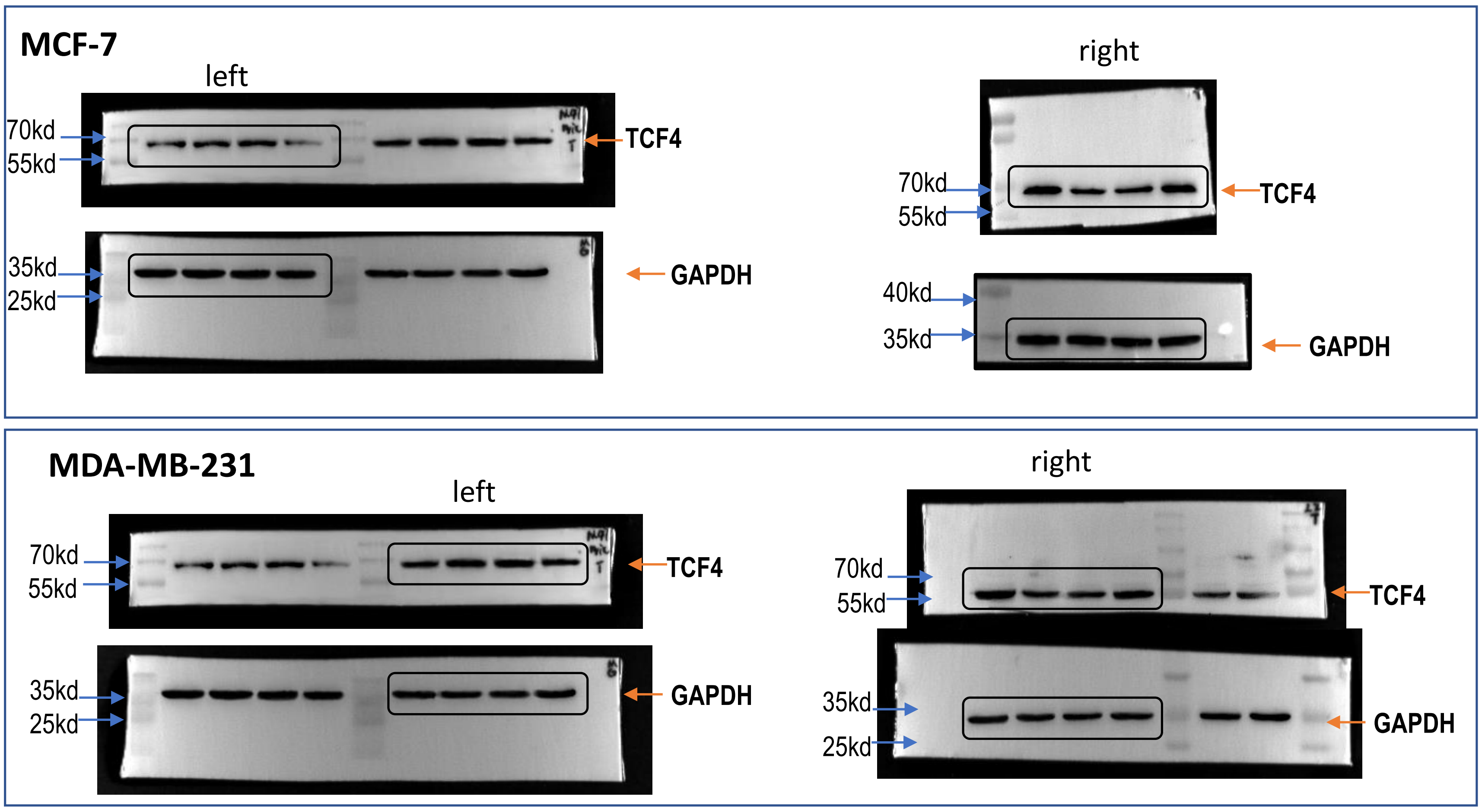

Figure 3K

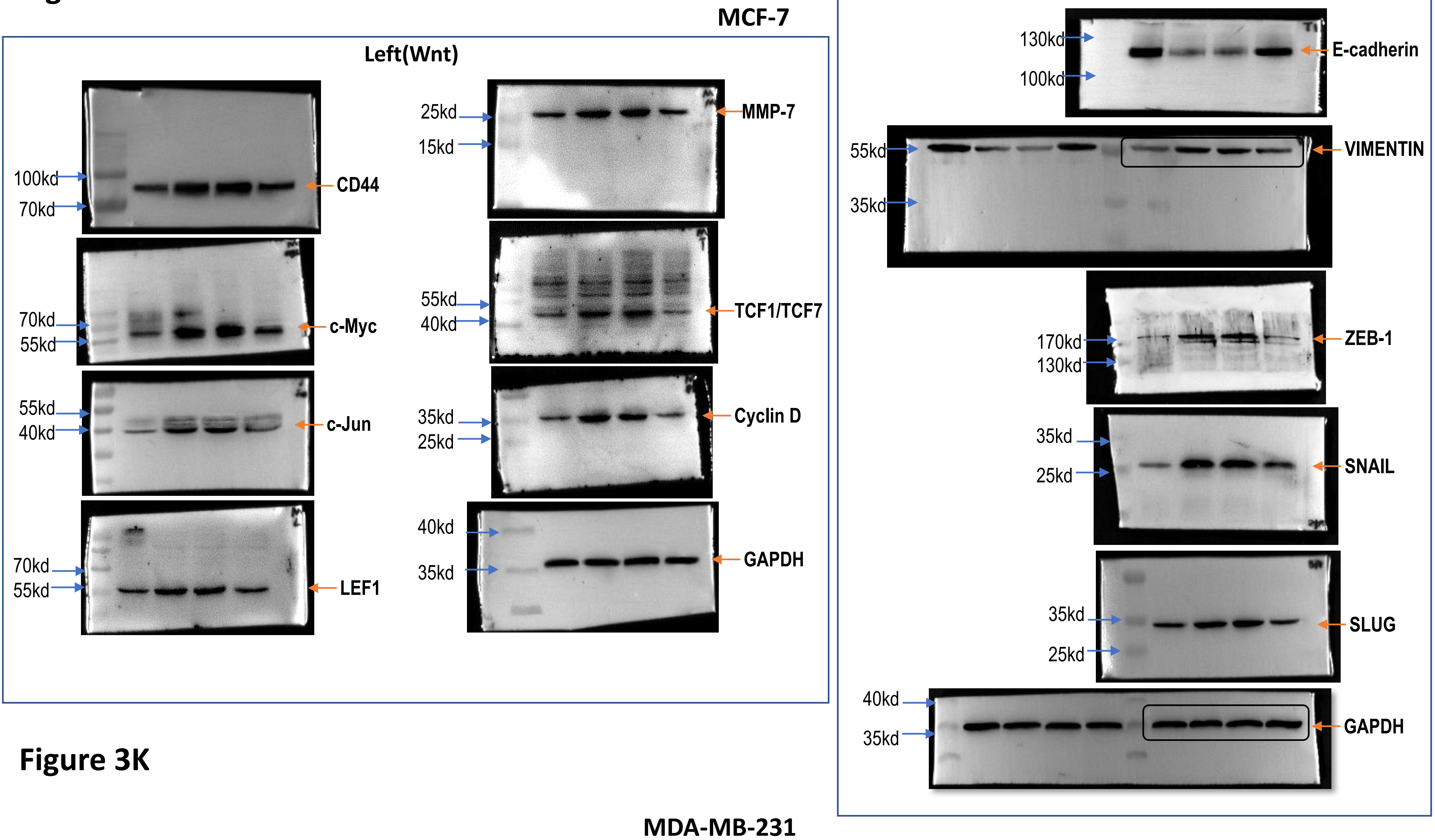

Figure 3K

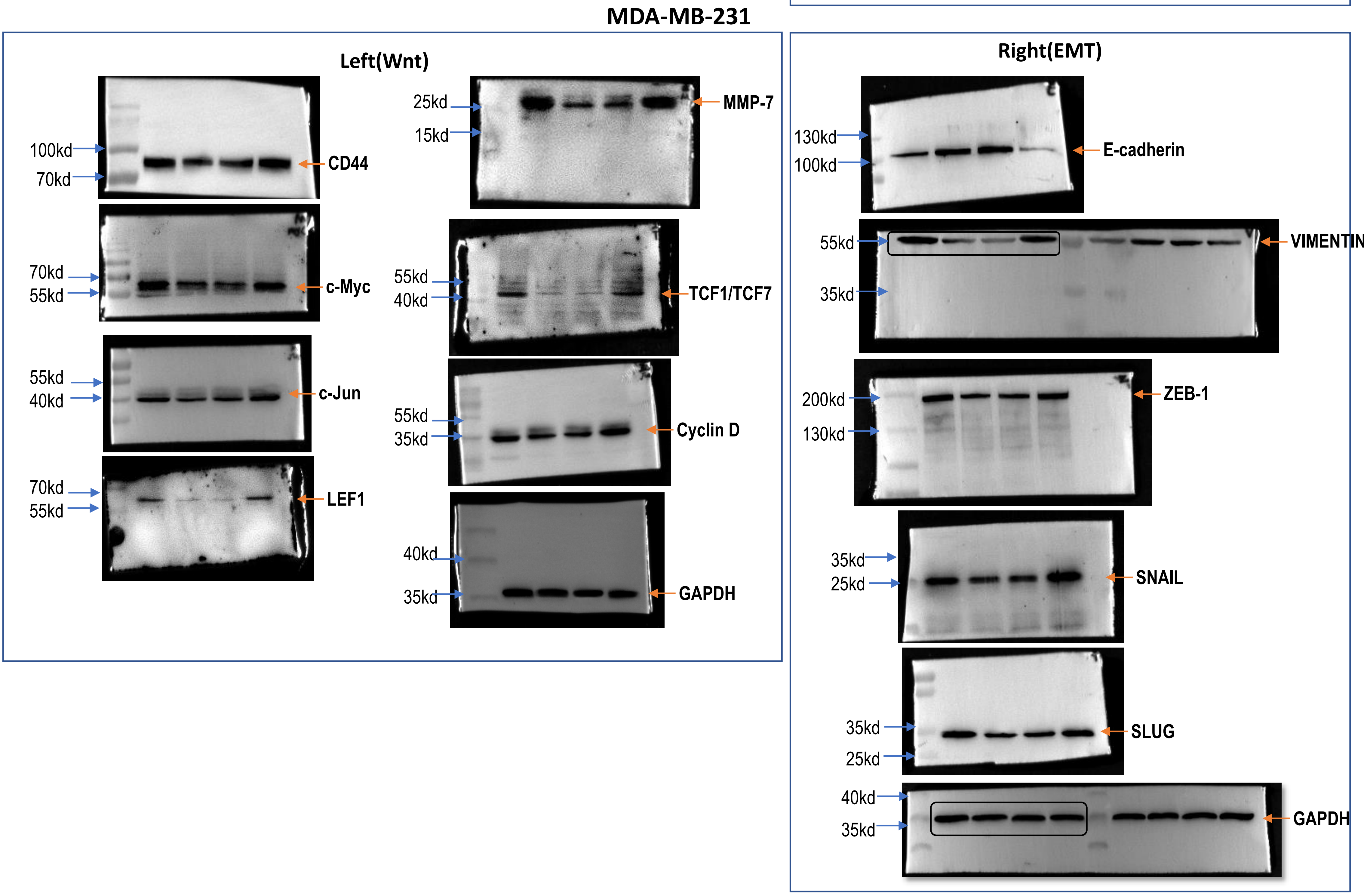

Figure 3M

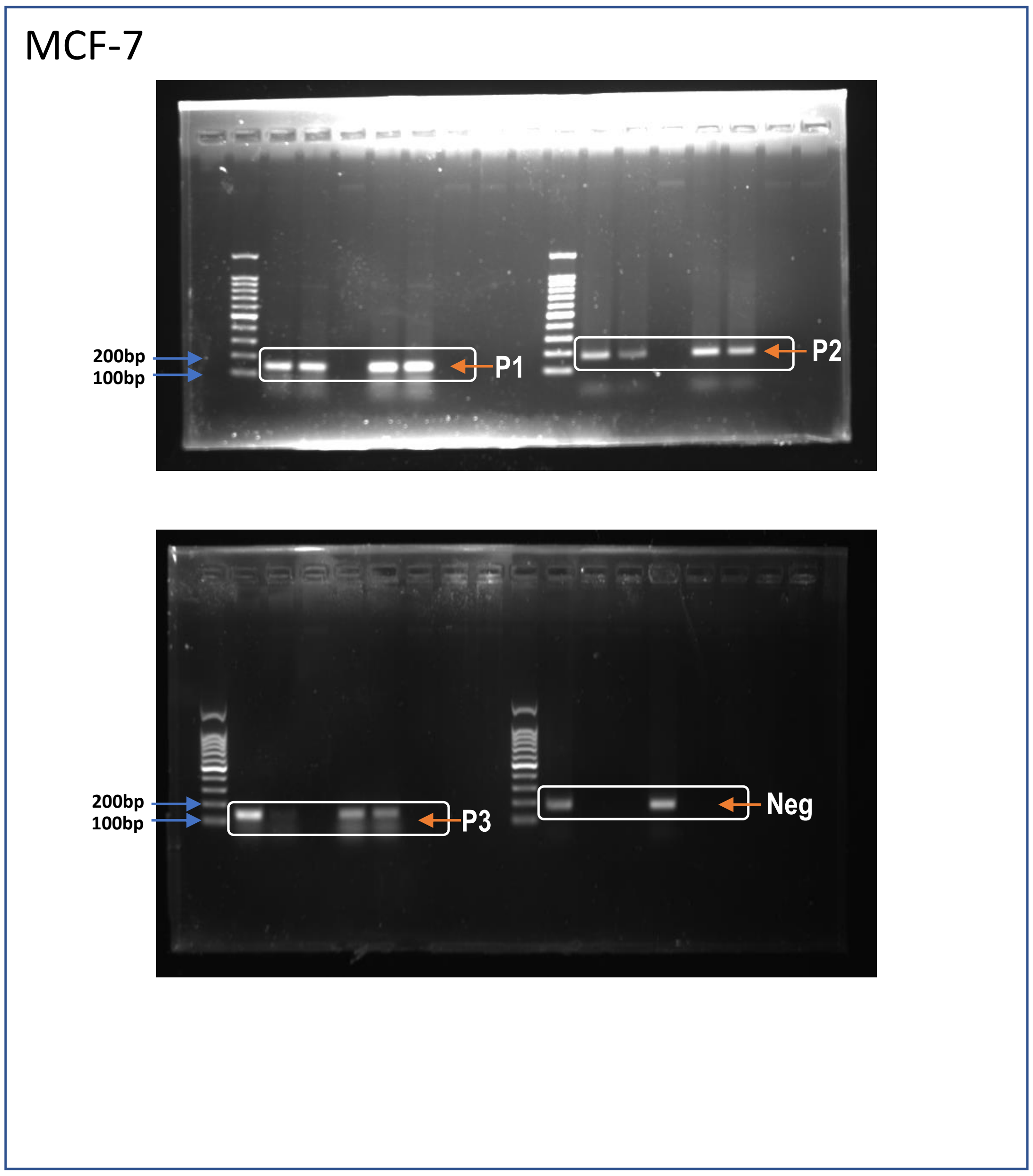
MCF-7
200bp
100bp
P1
P2
200bp
100bp
P3
Neg

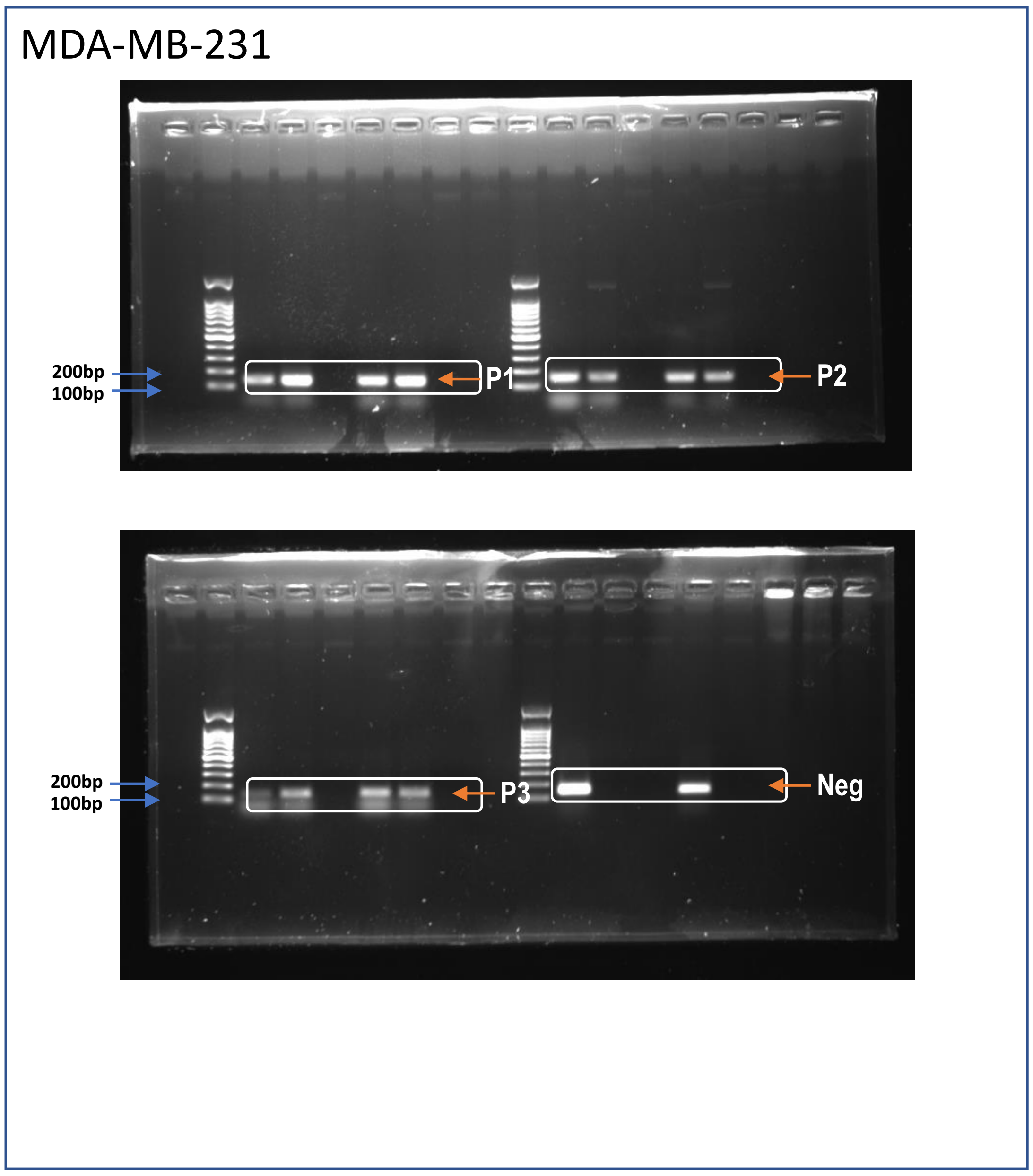
MDA-MB-231
200bp
100bp
P1
P2
200bp
100bp
P3
Neg

Figure 4H

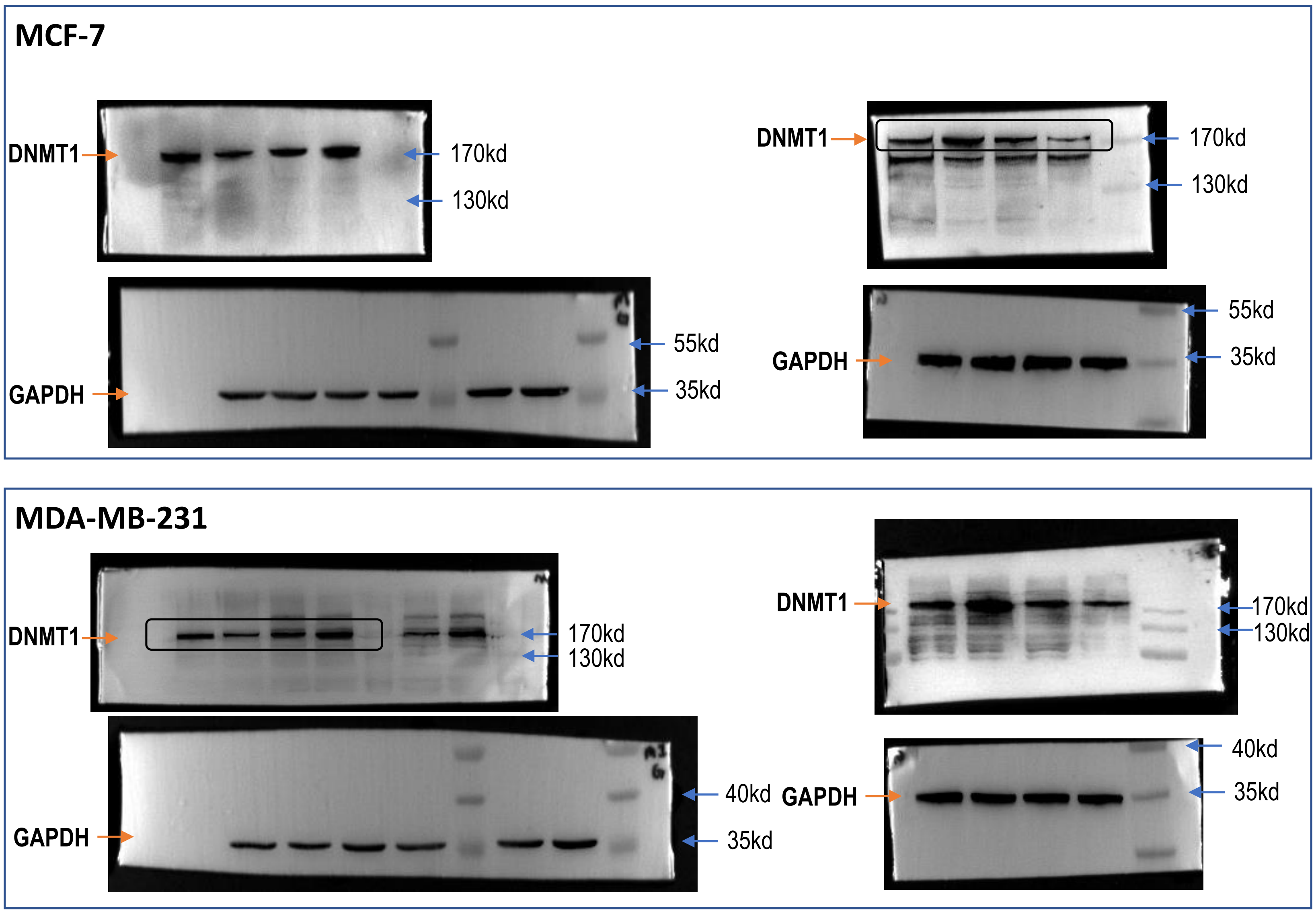

Figure 4I

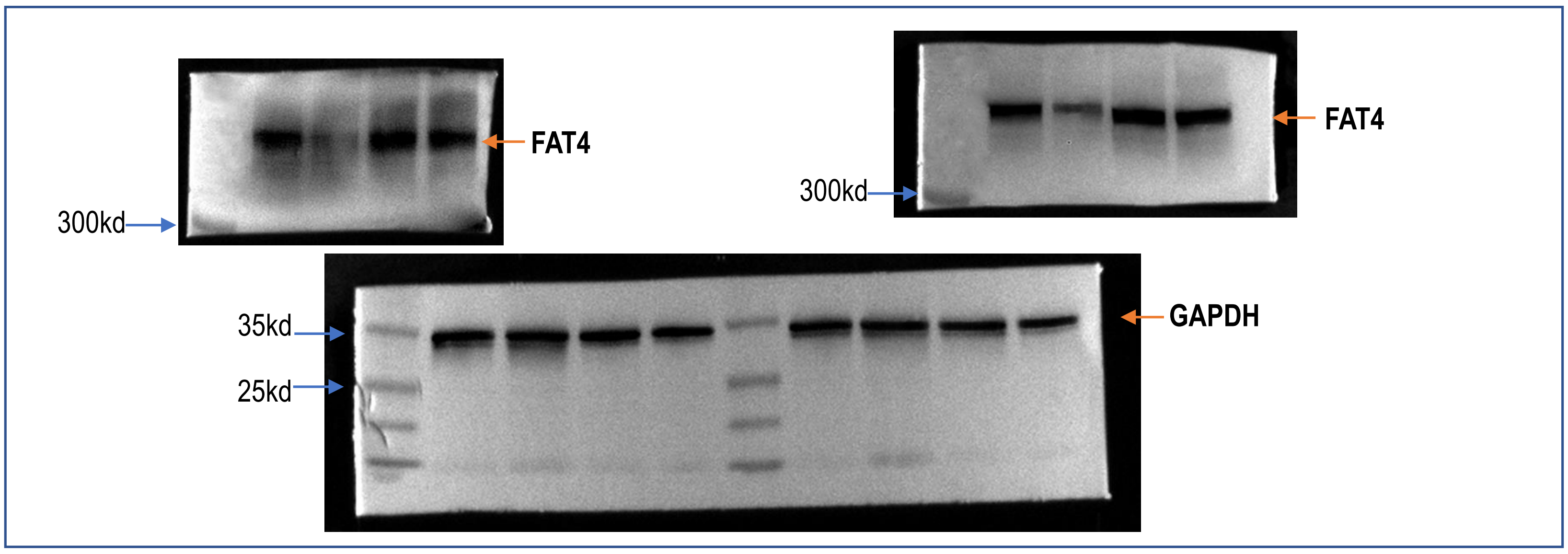

Figure 4K

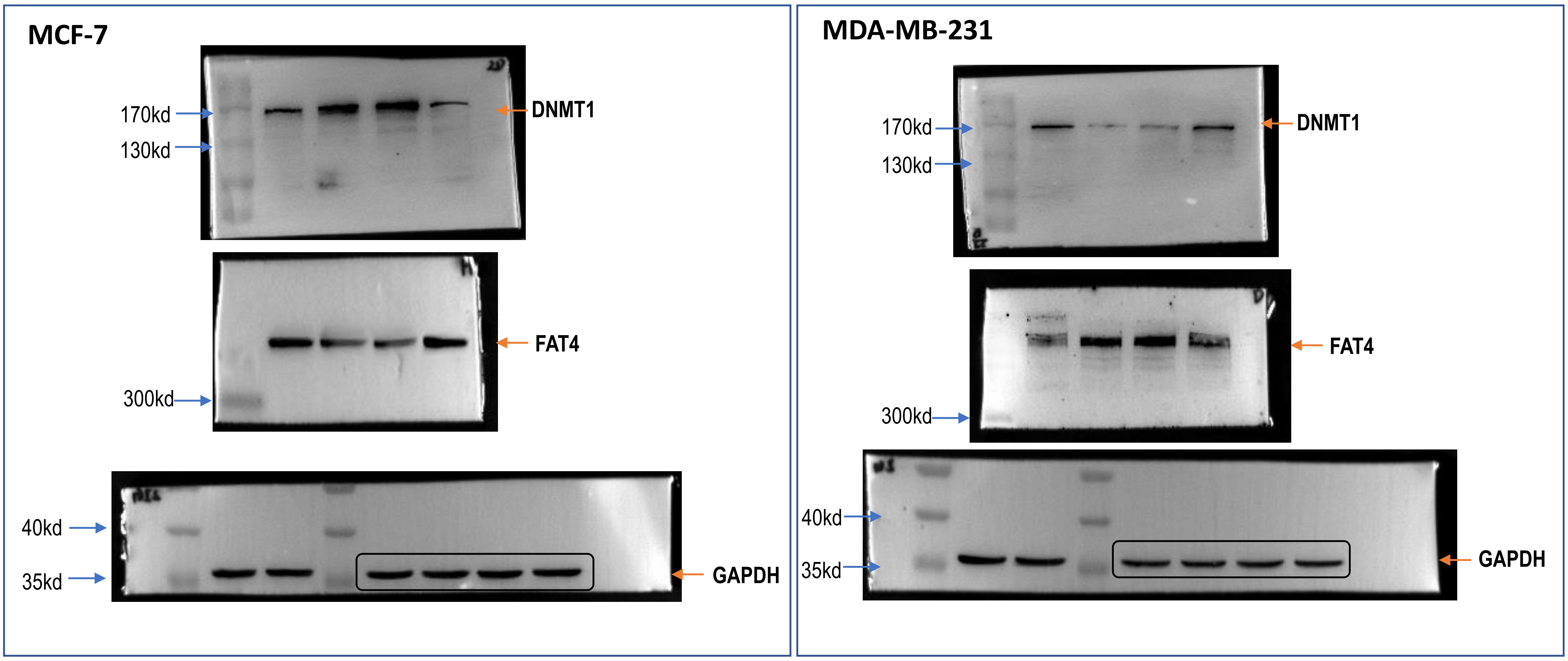

Figure 4L

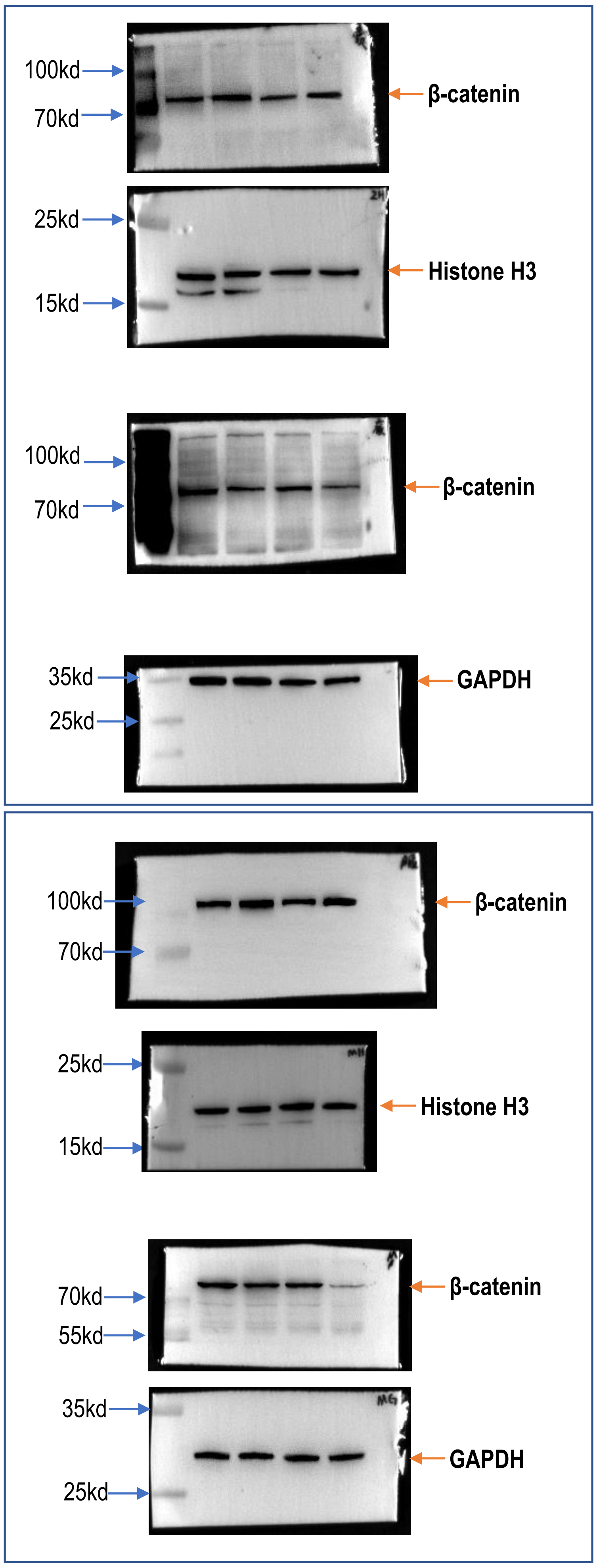

Figure 4N

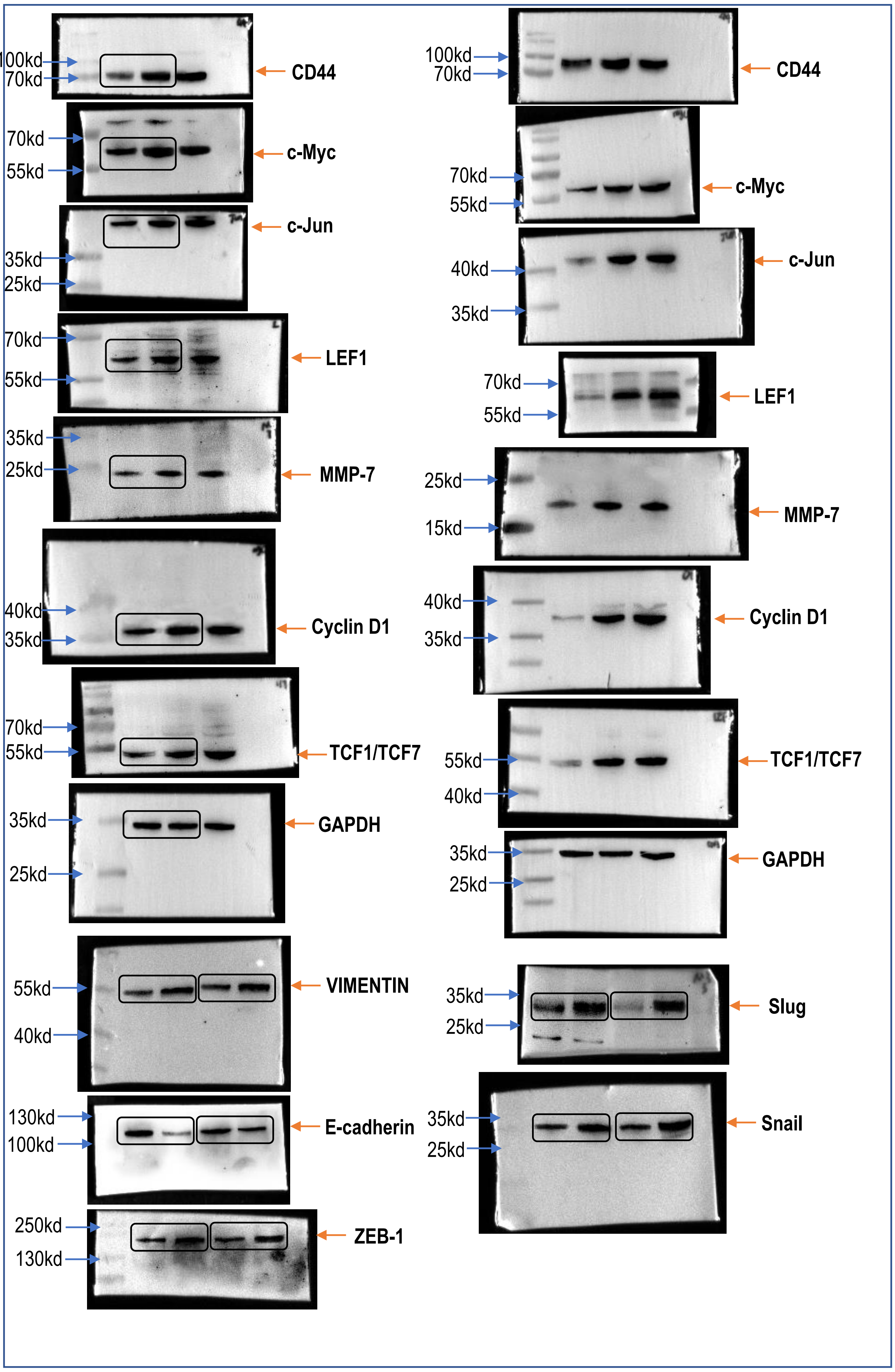

Figure 4O

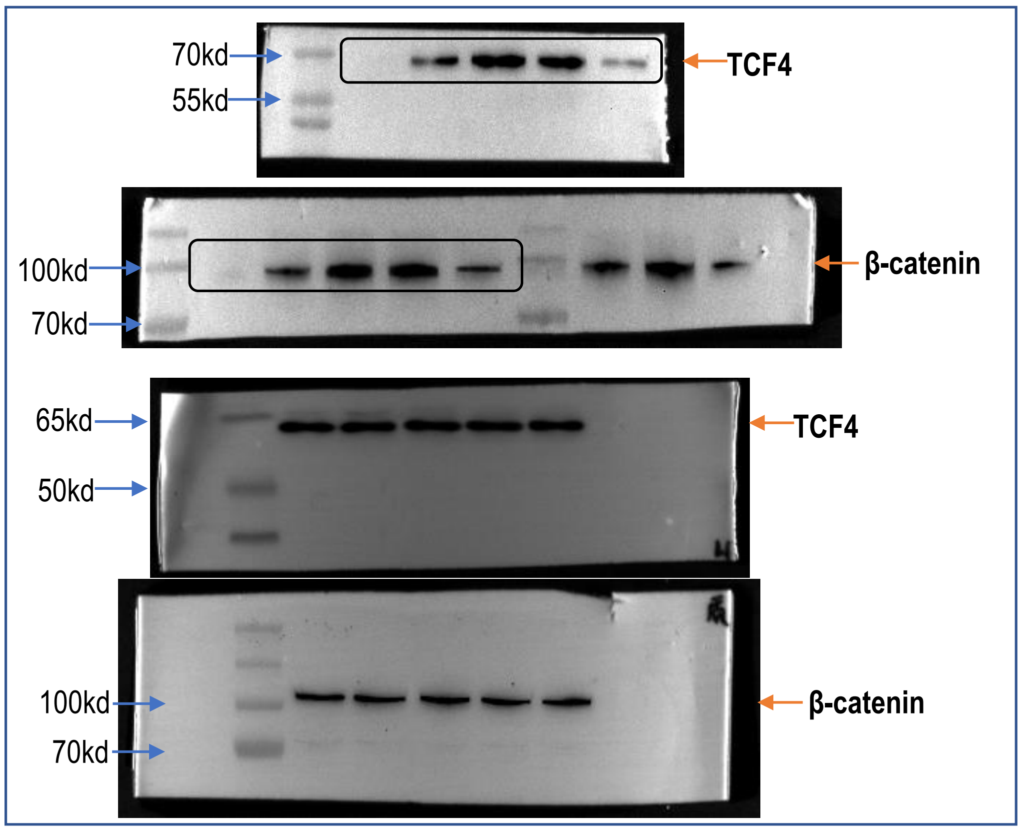

Figure 5B

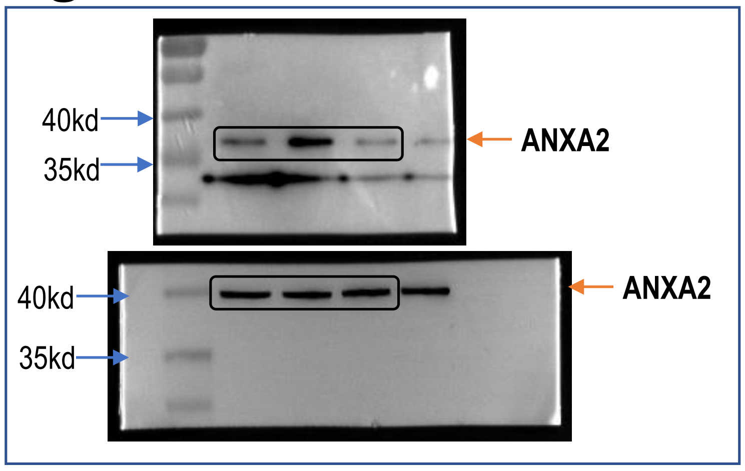

Figure 5E

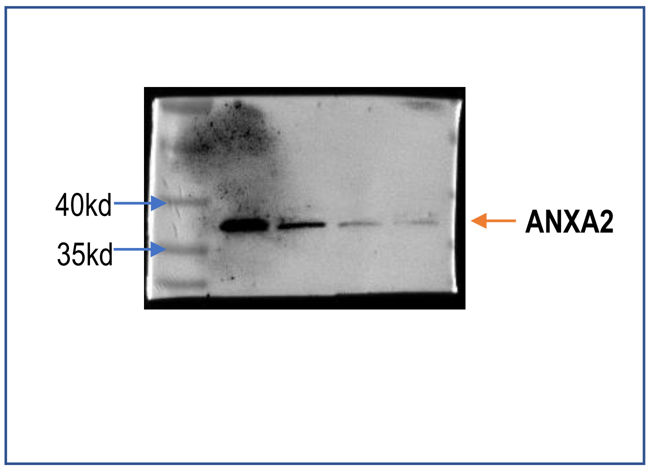

Figure 5F

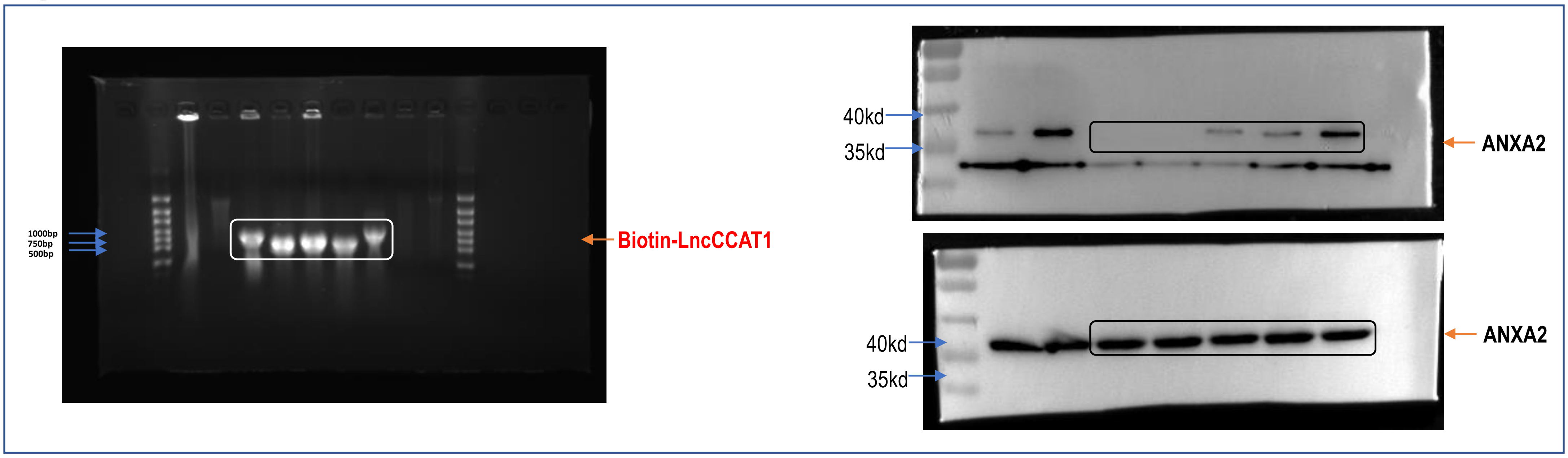

Figure 5K

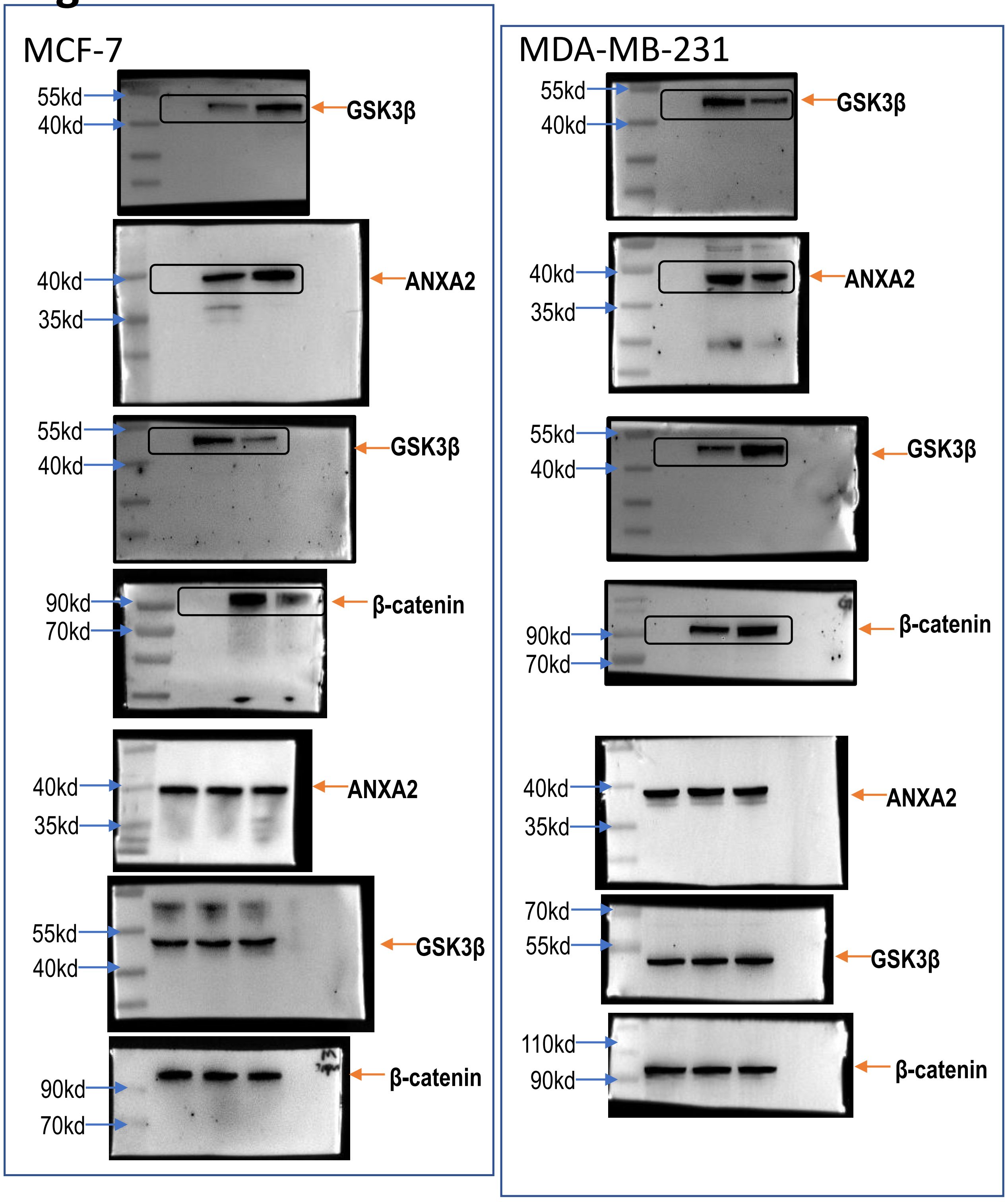

Figure 5I

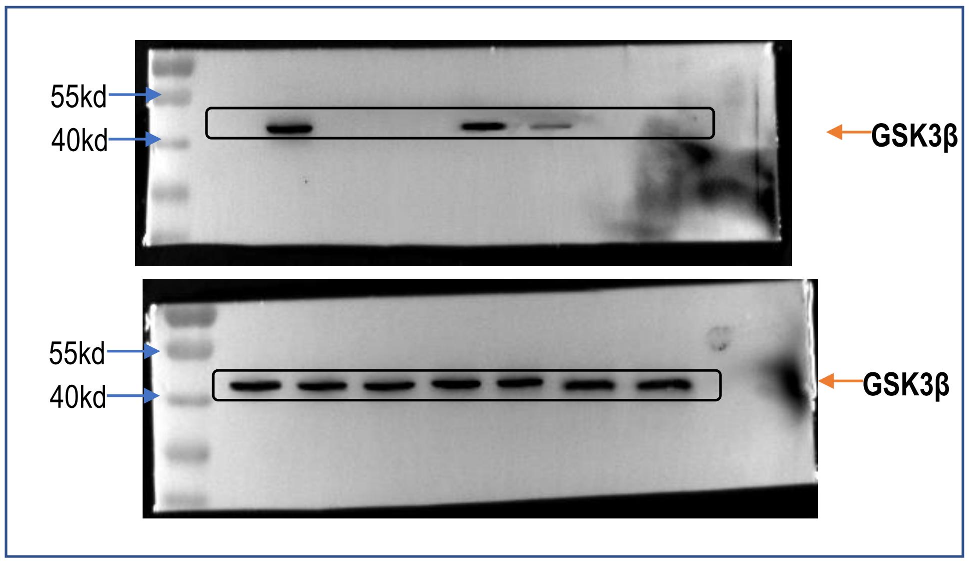

Figure 5L

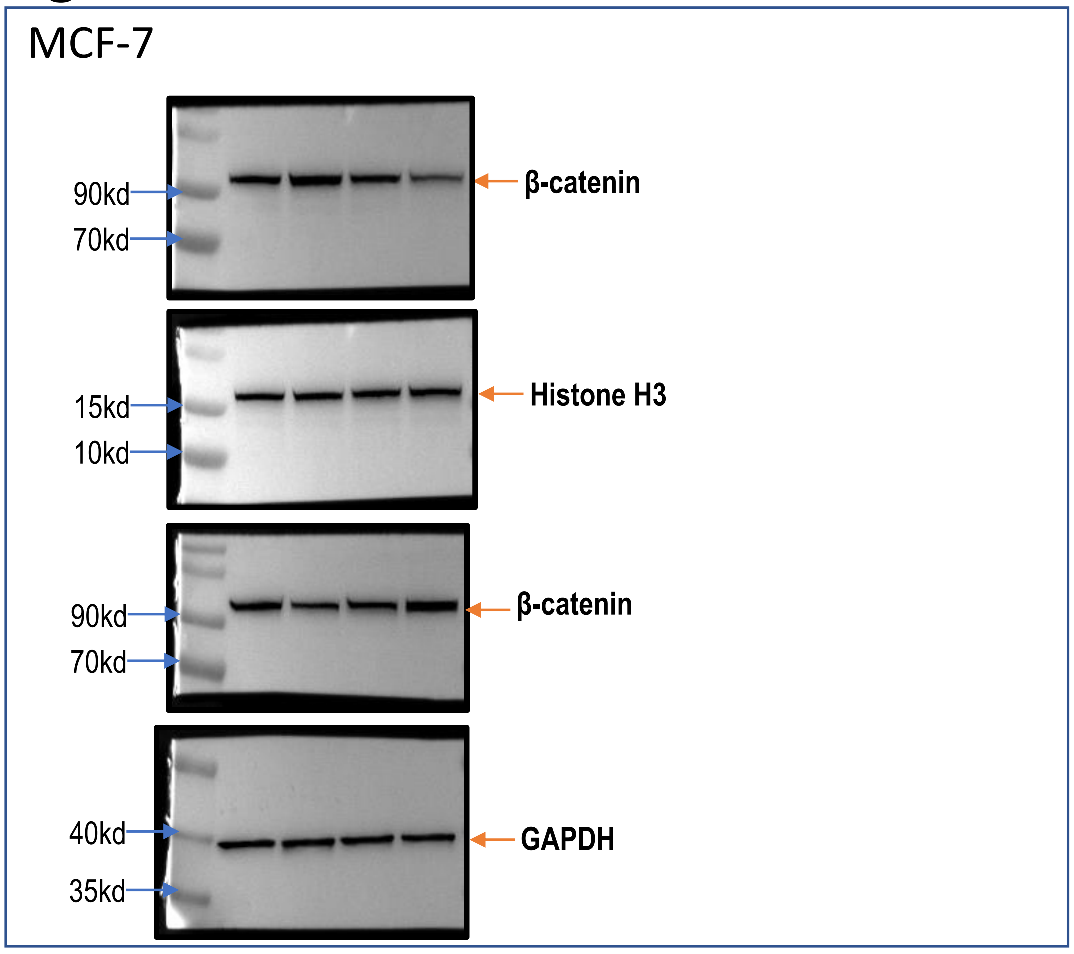

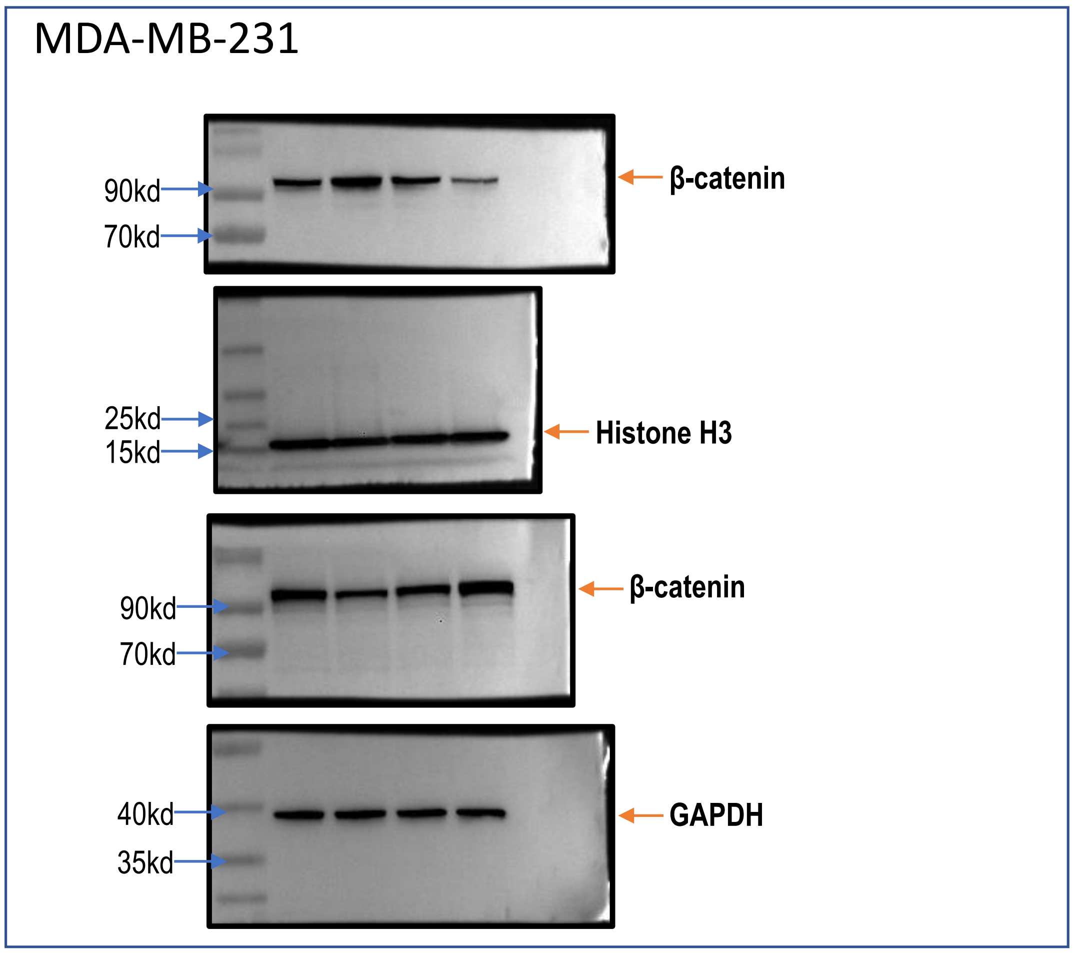

Figure 5M

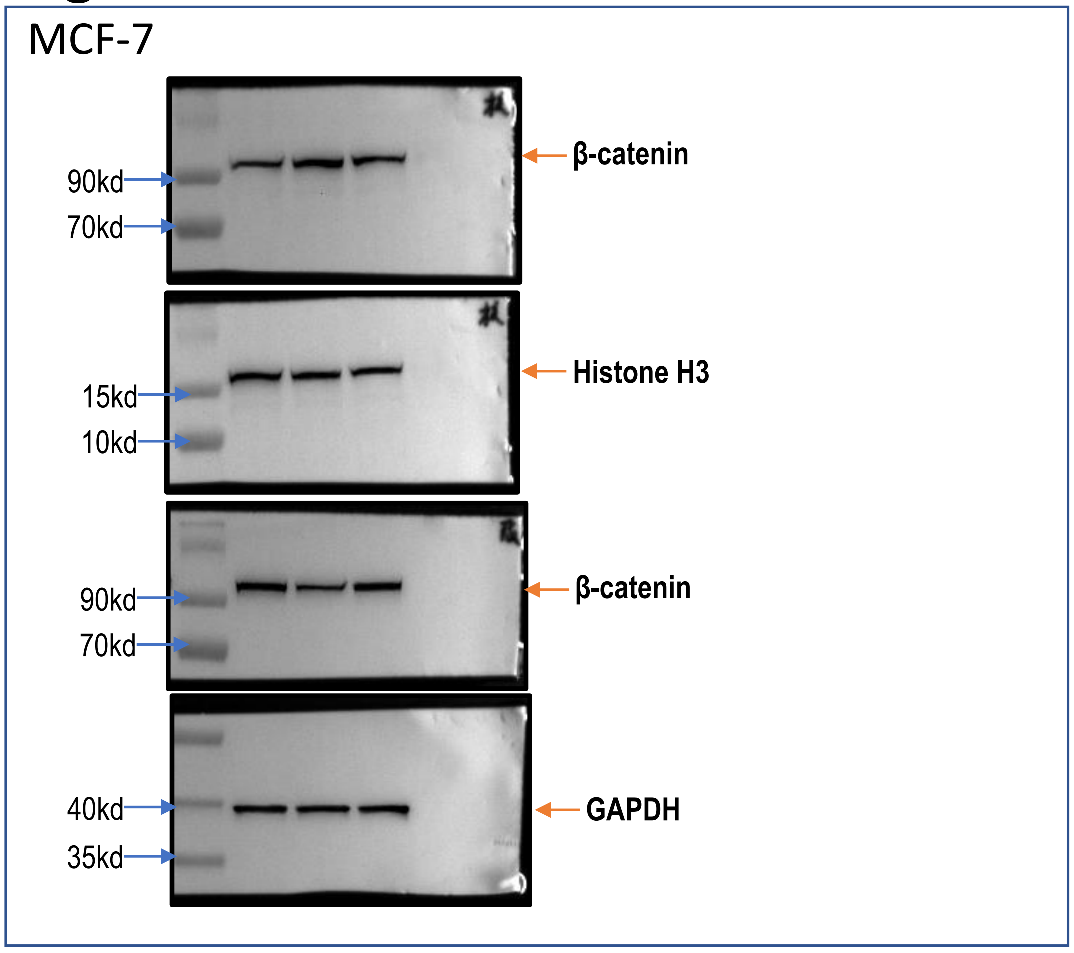

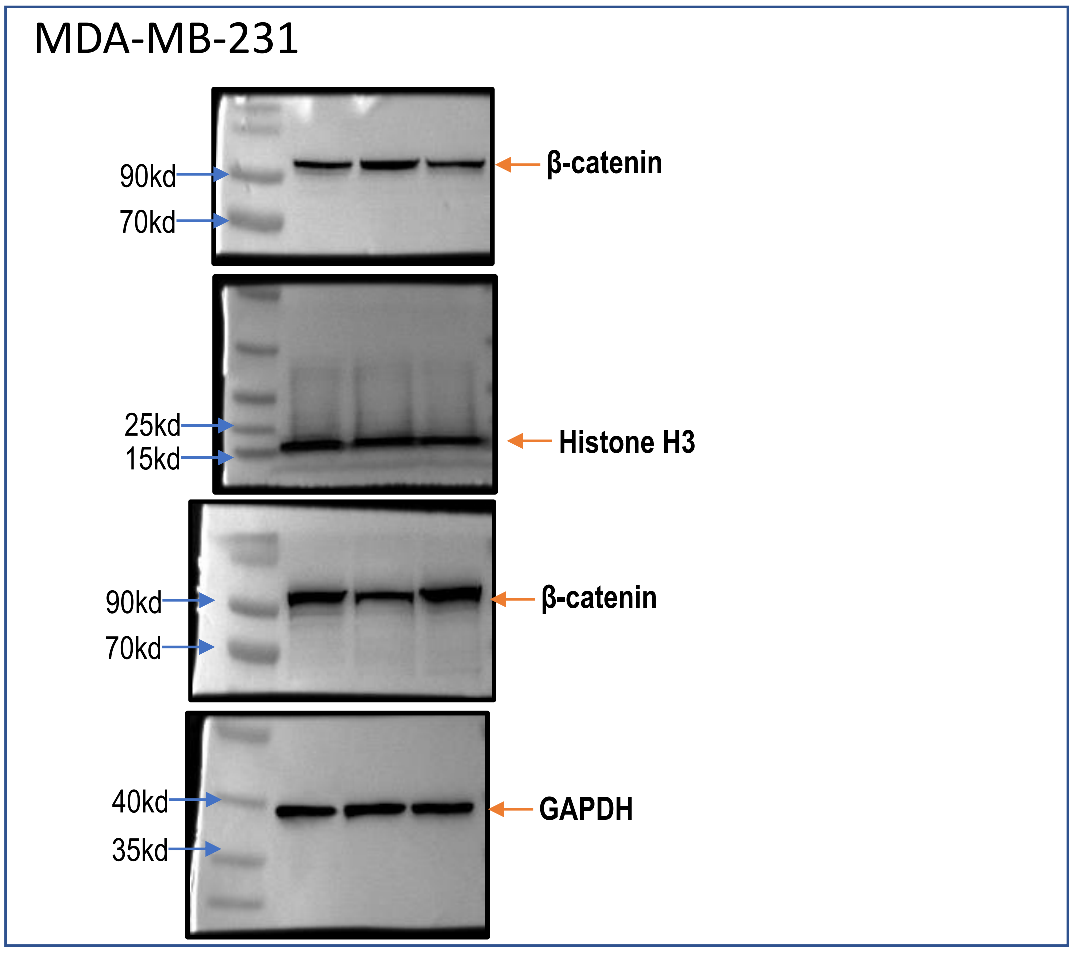

Figure 5N

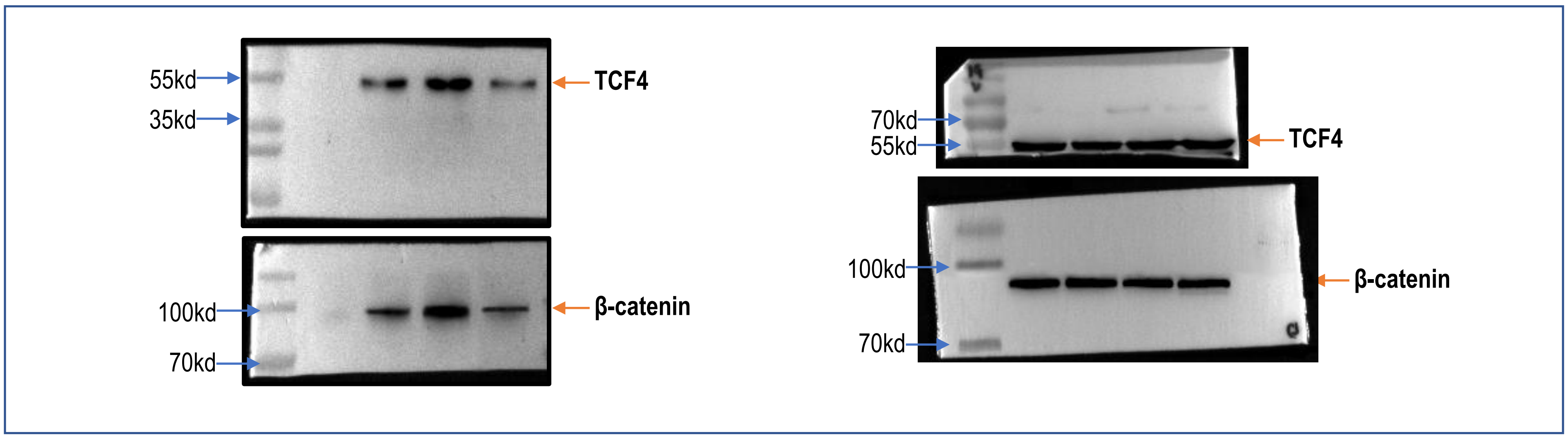

Figure S3A

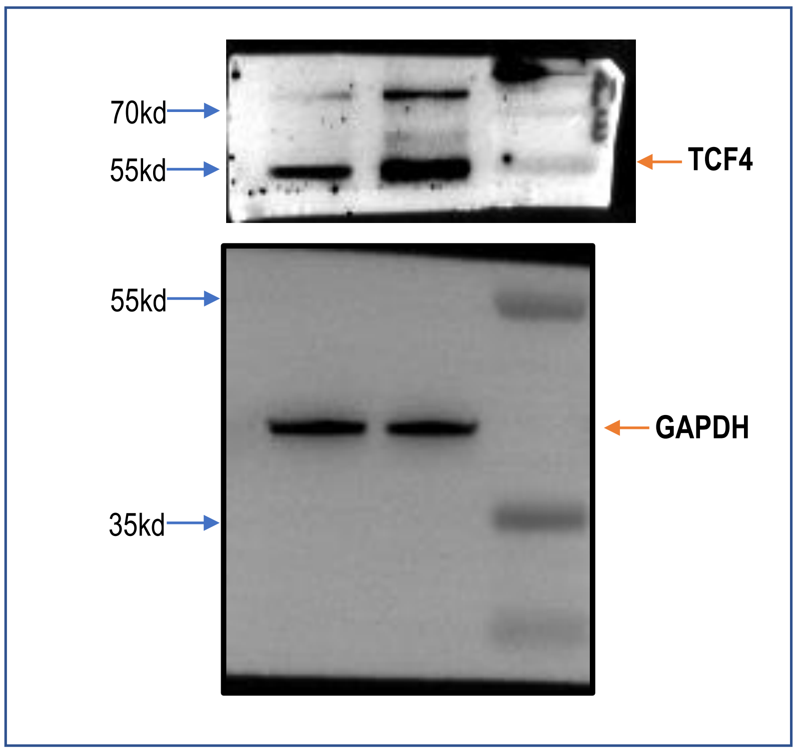

Figure S3B

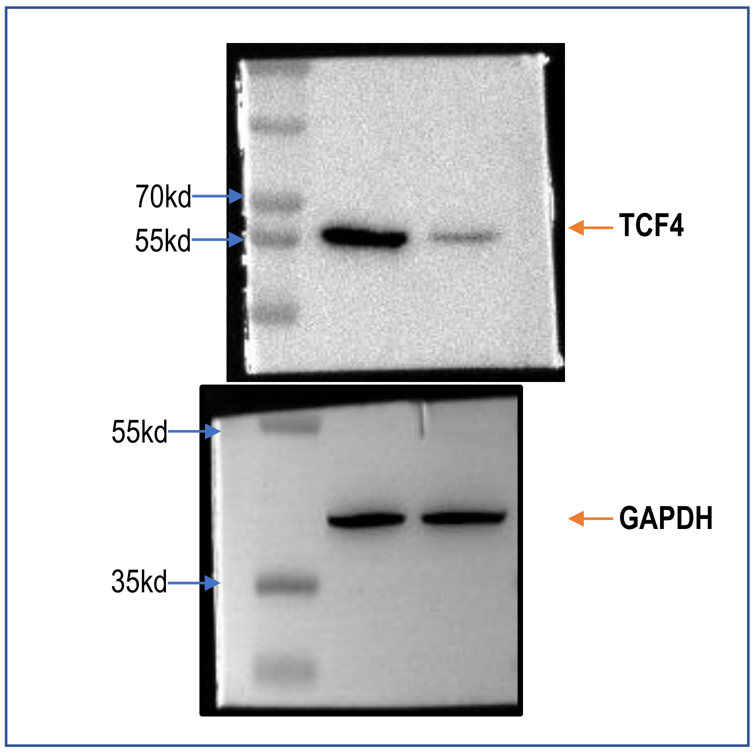

Figure S5C

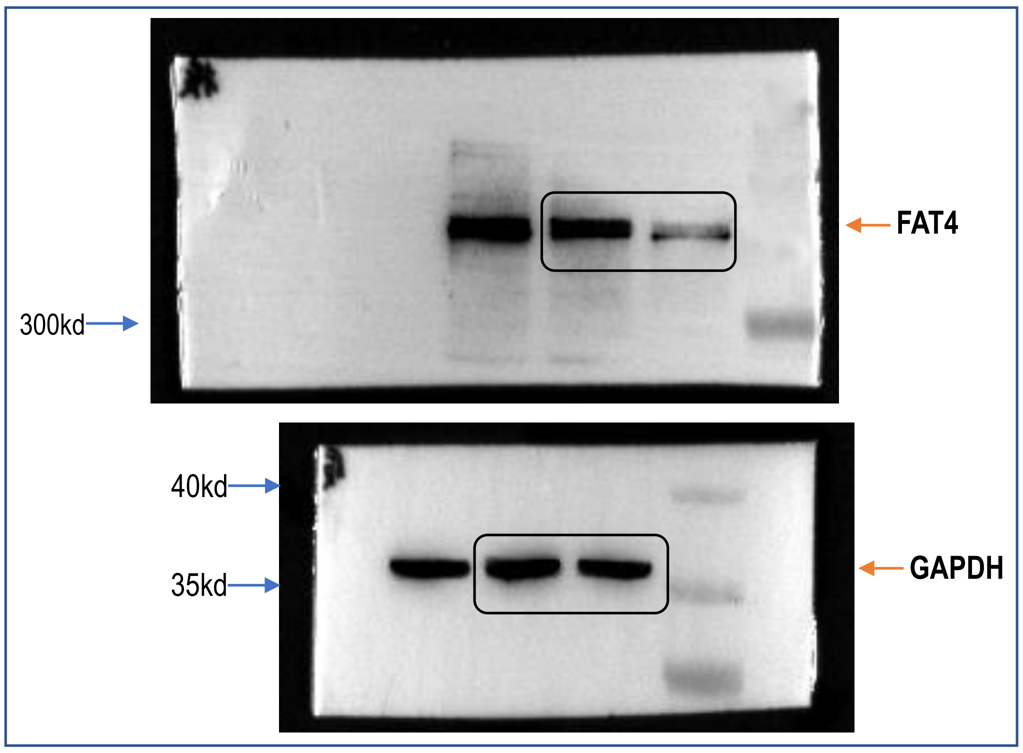

Figure S5D

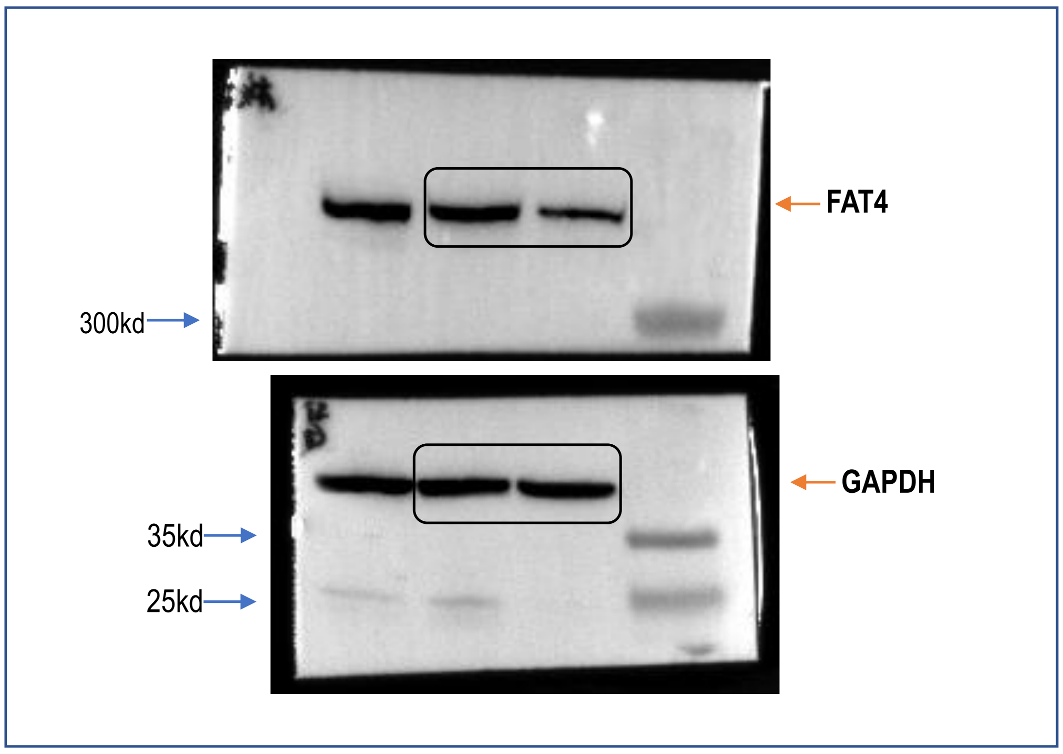

Figure S6B

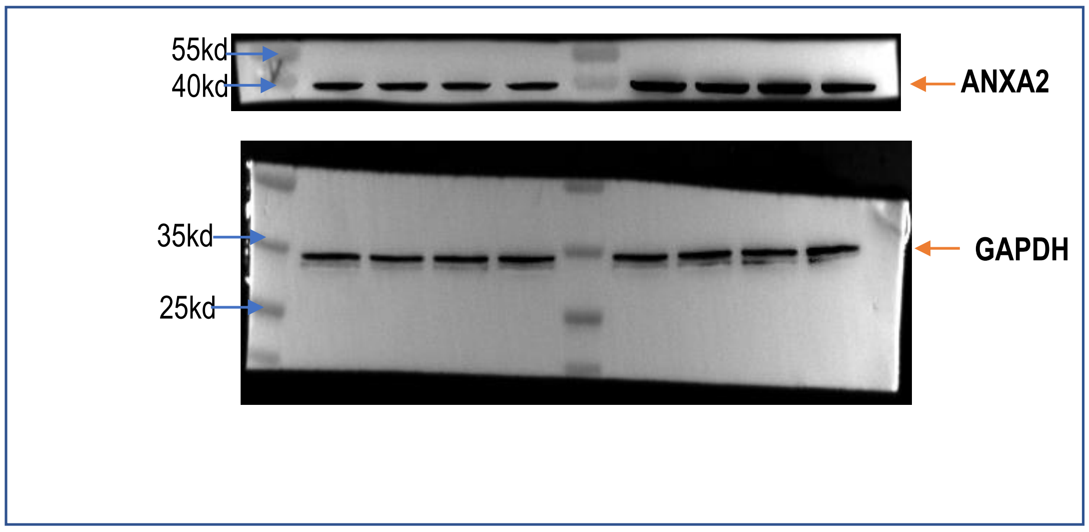

Figure S6C

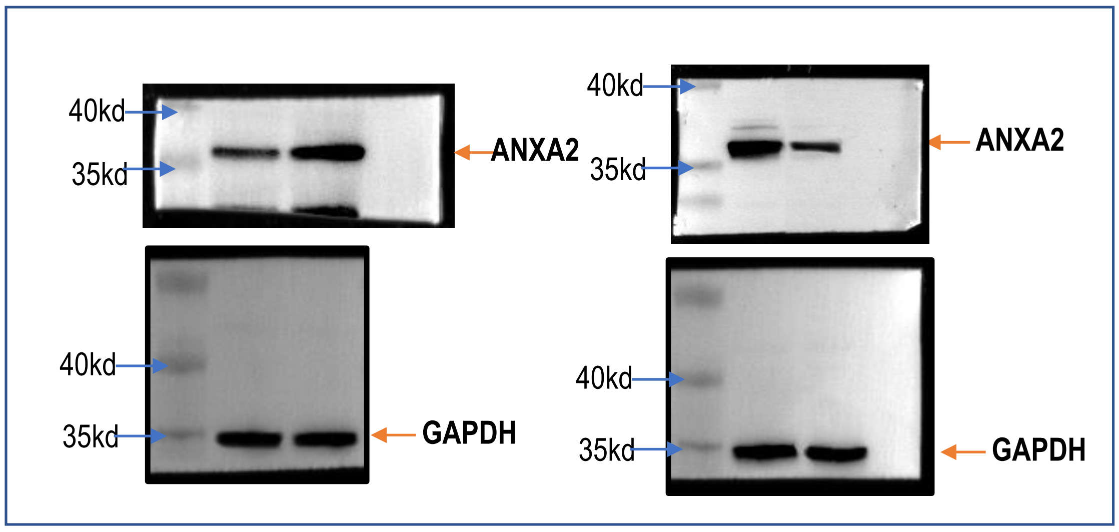

Figure S6G

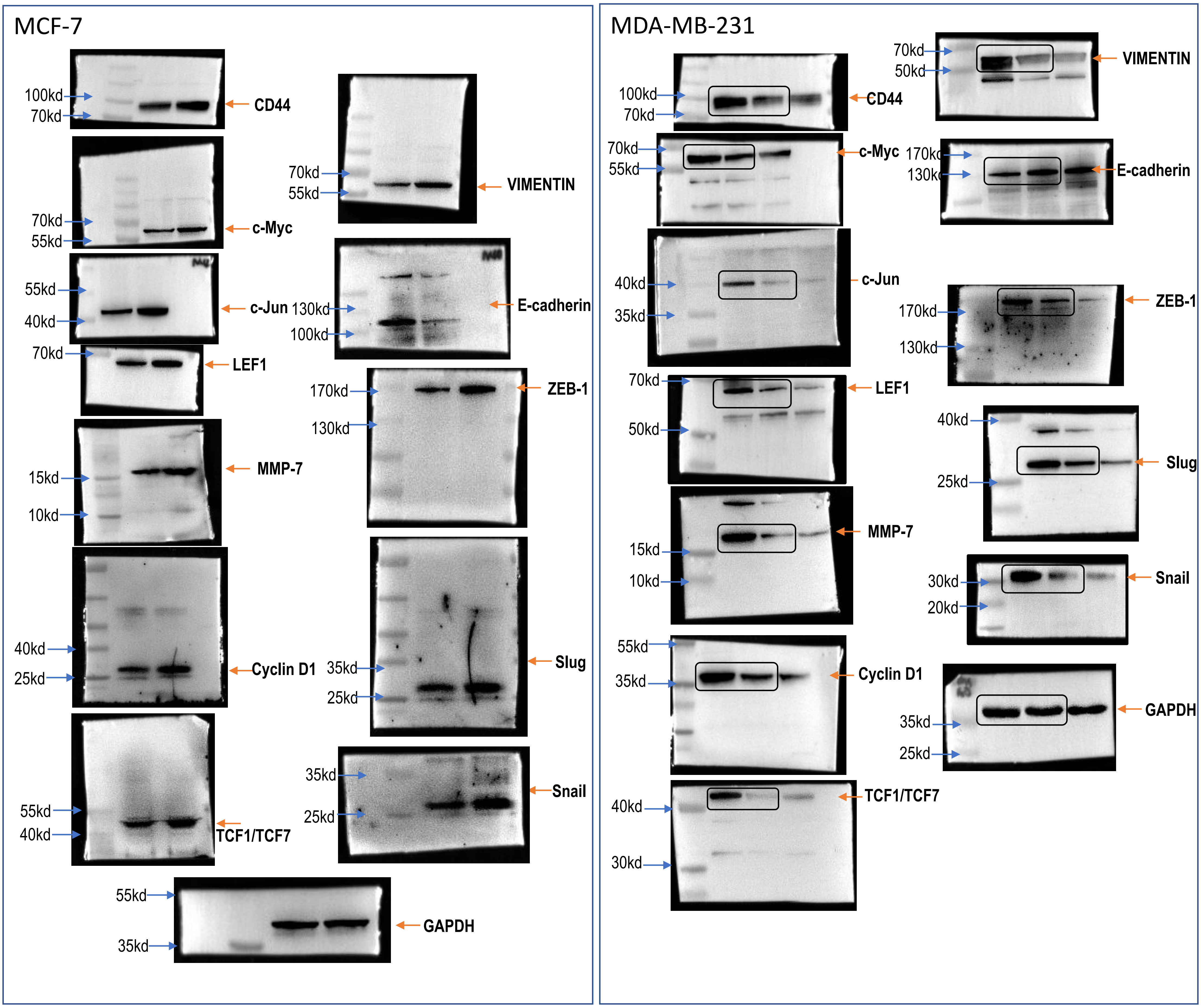

Molecular weight of all the protein：

GAPDH: 37 KD; **Sox2:** 34KD; **4-Oct:** 52KD; **Nanog:** 40KD; **ALDH1A1:** 56KD; **DNMT1:** 183KD; **TCF4:** 58KD; **FAT4:** 543KD; **CD44:** 80KD; **c-Myc:** 57-65KD; **c-Jun:** 43KD; **LEF1:** 55KD; **MMP-7:** 20-22KD; **Cyclin D1:** 36KD; **TCF1/TCF7:** 48,50KD; **β-catenin:** 92KD; **Histone H3:** 17KD; **E-cadherin:** 135KD; **Vimentin:** 54KD; **ZEB-1:** 200KD; **Slug:** 30KD; **Snail:** 29KD; **ANXA2:** 38KD; **GSK-3β:** 46KD.

Cell Line Authentication Report

Institution：CPU

Customer：Tingting Tang

Tel：15295783878

Email：1095477598@qq.com

Vendor：Cobioer Biosciences CO.,LTD

Attention：Taohua Jiang

Tel：025-86880024

Email：jiangtaohua@cobioer.com

1. Sample information:

Cell pellets，No.:“MCF-7”

2. Methods:

a. Genomic DNA was extracted from the cell pellets provided by the customer.
b. Samples, together with positive and negative control were amplified using GenePrint System (Promega).
c. Amplified products were processed using the ABI3730xl Genetic Analyzer.
d. Data was analyzed by GeneMapper4.0 software and then compared with the ATCC, DSMZ or JCRB databases for reference matching.

3. Results:

STR profile

Marker	Sample				Cobioer Database		
	Allele1	Allele2	Allele3	Allele4	Allele1	Allele2	Allele3
D5S818	11	12			11	12	
D13S317	11	11			11	11	
D7S820	8	9			8	9	
D16S539	11	12			11	12	
VWA	14	15			14	15	
TH01	6	6			6	6	
AMEL	X	X			X	X	
TPOX	9	12			9	12	
CSF1PO	10	10			10	10	
D12S391	18	20					
FGA	23	24	25				
D2S1338	21	23					
D21S11	30	30					
D18S51	14	14					
D8S1179	10	14					
D3S1358	16	16					
D6S1043	12	18					
PENTAE	7	12					
D19S433	13	14					
PENTAD	12	12					

4. Conclusion

A. The STR results show that there are no four alleles on the main nine locus, there is certainly no cross contamination of human cells in this cell line.

B. The match percent between the sample and the STR database profile is **100%**, the cell name is **MCF-7.**

EV	Cell name	Locus names								
		D5S818	D13S317	D7S820	D16S539	VWA	TH01	AM	TPOX	CSF1PO
Query	(Your Cell)	11, 12	11, 11	8, 9	11, 12	14, 15	6, 6	X, X	9, 12	10, 10
1.00 (36/36)	MCF-7	11, 12	11, 11	8, 9	11, 12	14, 15	6, 6	X, X	9, 12	10, 10

Database Link: http://139.196.13.241:8081/index

Note:

1. The STR profile data was compared with the ATCC, DSMZ or JCRB databases, if the cell line is not included in the three institutions, the results are not correct. More information you provided would be useful for Cell Line Authentication.
2. Based on the ANSI Standard, cell lines with 100% match are considered to be "identical"; cell lines with ≥80% but less than 100% match are considered to be "related".

Operator: Xiaomei Liu, Qin Wang

Leader: Wei Zhou

Date: 2018-05-09

Cell Line Authentication Report

Institution：CPU

Customer：Tingting Tang

Tel：15295783878

Email：1095477598@qq.com

Vendor：Cobioer Biosciences CO.,LTD

Attention：Taohua Jiang

Tel：025-86880024

Email：jiangtaohua@cobioer.com

1. Sample information:

Cell pellets，No.:“MDA-MB-231”

2. Methods:

a. Genomic DNA was extracted from the cell pellets provided by the customer.
b. Samples, together with positive and negative control were amplified using GenePrint System (Promega).
c. Amplified products were processed using the ABI3730xl Genetic Analyzer.
d. Data was analyzed by GeneMapper4.0 software and then compared with the ATCC, DSMZ or JCRB databases for reference matching.

3. Results:

STR profile

Marker	Sample				Cobioer Database		
	Allele1	Allele2	Allele3	Allele4	Allele1	Allele2	Allele3
D5S818	12	12			12	12	
D13S317	13	13			13	13	
D7S820	8	9			8	9	
D16S539	12	12			12	12	
VWA	15	18			15	18	
TH01	7	9.3			7	9.3	
AMEL	X	X			X	X	
TPOX	8	9			8	9	
CSF1PO	12	13			12	13	
D12S391	17	18					
FGA	22	23					
D2S1338	20	21					
D21S11	33.2	33.2					
D18S51	11	16					
D8S1179	13	13					
D3S1358	16	16					
D6S1043	18	18					
PENTAE	11	11					
D19S433	11	14					
PENTAD	11	14					

4. Conclusion

A． The STR results show that there are no four alleles on the main nine locus, there is certainly no cross contamination of human cells in this cell line.

B． The match percent between the sample and the STR database profile is **100%**, the cell name is **MDA-MB-231.**

EV	Cell name	Locus names								
		D5S818	D13S317	D7S820	D16S539	VWA	TH01	AM	TPOX	CSF1PO
Query	(Your Cell)	12,12	13,13	8,9	12,12	15,18	7,9.3	X,X	8,9	12,13
1.00(36/36)	MDA-MB-231	12,12	13,13	8,9	12,12	15,18	7,9.3	X,X	8,9	12,13

Database Link: http://139.196.13.241:8081/index

Note:

1. The STR profile data was compared with the ATCC, DSMZ or JCRB databases, if the cell line is not included in the three institutions, the results are not correct. More information you provided would be useful for Cell Line Authentication.
2. Based on the ANSI Standard, cell lines with 100% match are considered to be "identical"; cell lines with ≥80% but less than 100% match are considered to be "related".

Operator：Xiaomei Liu, Qin Wang

Leader：Wei Zhou

Date：2018-05-15
